# Thermodynamic equilibrium between locally excited and charge transfer states in perylene–phenothiazine dyads

**DOI:** 10.3762/bjoc.21.121

**Published:** 2025-08-05

**Authors:** Issei Fukunaga, Shunsuke Kobashi, Yuki Nagai, Hiroki Horita, Hiromitsu Maeda, Yoichi Kobayashi

**Affiliations:** 1 Department of Applied Chemistry, College of Life Sciences, Ritsumeikan University, 1-1-1 Nojihigashi, Kusatsu, Shiga, Japan

**Keywords:** charge transfer, electron transfer, molecular dyad, transient absorption

## Abstract

We report the excited-state dynamics of π-orthogonal donor–acceptor dyads based on perylene (Pe) and phenothiazine (PTZ), in which triphenylamine (TPA) units and a phenyl spacer were introduced to modulate donor strength and spatial separation. Among the series, Pe–PTZ(TPA)_2_ exhibits a distinct thermal equilibrium between the locally excited (LE) state of the PTZ moiety and the photoinduced charge-transfer (CT) state. Femtosecond to microsecond transient absorption spectroscopy reveals that this equilibrium is facilitated not simply by enhanced donor ability, but presumably by excited-state planarization of the PTZ moiety, which lowers the energy of the LE state of the PTZ moiety. In contrast, Pe-Ph–PTZ(TPA)_2_, in which the donor–acceptor distance is increased by a phenyl spacer, does not show clear equilibrium behavior. These results underscore the crucial role of excited-state structural relaxation in tuning photoinduced charge separation, and demonstrate that precise electronic and geometric design can enable controllable excited-state behavior in orthogonal molecular systems.

## Introduction

Photoinduced electron transfer and charge separation are fundamental processes underlying a wide range of applications, including artificial photosynthesis, solar energy conversion, and photocatalysis [1–3]. In particular, the formation of long-lived charge-separated states is crucial for efficient energy conversion and advanced photofunctions driven by light. Among various donor–acceptor (D–A) architectures, π-orthogonal molecules, where the donor and acceptor moieties are spatially decoupled, have attracted considerable attention [4–7]. This orthogonal arrangement can minimize ground-state electronic interactions while facilitating efficient photoinduced charge separation and spin–orbit charge-transfer intersystem crossing (SOCT-ISC), leading to unique excited-state behavior.

An important aspect of D–A systems is the interplay between the locally excited (LE) and the charge-transfer (CT) states (denoted as diabatic states). A thermal equilibrium between local excited (LE) and charge-transfer (CT) states can be experimentally observed as dual fluorescence: a structured, higher-energy emission from the LE state and a broad, red-shifted emission from the CT state [8–9]. Classical systems such as 4-(*N*,*N*-dimethylamino)benzonitrile (DMABN) and pyrene-*o*-carborane exhibit such behavior, where LE and CT states dynamically interconvert depending on solvent polarity and temperature [10–12]. When the interconversion is faster than radiative decay, the two states can reach thermodynamic equilibrium, and their relative populations are determined by the energy gap between them.

Phenothiazine (PTZ) and perylene (Pe) are well-known molecular components for designing π-orthogonal D–A systems [13–15]. PTZ is an excellent electron donor due to its strong electron-donating ability and redox tunability, while Pe is often used as a robust acceptor featuring high photostability and strong visible absorption. Perylene–phenothiazine (Pe–PTZ) derivatives have been previously investigated for their excited-state dynamics [15].

Incorporating additional electron-donating groups such as triphenylamine (TPA) onto the PTZ core is expected not only to enhance the donor property but also to lower the energy of the LE state of the PTZ moiety. Notably, while PTZ adopts a non-planar conformation in the ground state, it undergoes planarization in the excited state [16–17]. This conformational change is expected to further enhance the stabilization effect imparted by TPA. Such stabilization could bring the energy levels of the LE state of the PTZ moiety and the CT state into closer proximity, which may create conditions favorable for establishing a transient thermal equilibrium between the LE and CT states.

**Figure 1:**
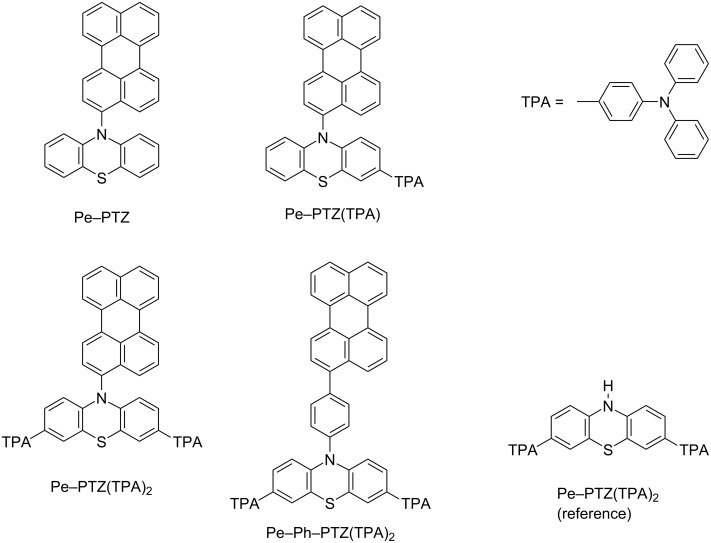
Molecular structures of Pe–PTZ, Pe–PTZ(TPA), Pe–PTZ(TPA)_2_, and Pe–Ph–PTZ(TPA)_2_.

In this study, we designed and synthesized a series of novel Pe–PTZ derivatives by incorporating electron-donating triphenylamine (TPA) units and phenyl spacers to systematically modulate the donor strength and the spatial distance between the donor and acceptor. Four compounds – Pe–PTZ, Pe–PTZ(TPA), Pe–PTZ(TPA)_2_, and Pe–Ph–PTZ(TPA)_2_ – were synthesized and characterized (Figure 1). Their photophysical properties were comprehensively examined by steady-state and time-resolved emission and absorption spectroscopy. Furthermore, time-dependent density functional theory (TD-DFT) calculations were performed to support the experimental findings. Through these investigations, we aim to deepen the understanding of how molecular design parameters such as donor strength, spacer introduction, and π-orthogonality influence excited-state equilibria and charge-transfer processes in π-orthogonal donor–acceptor systems.

## Results and Discussion

### Synthesis and structural characterization

A series of π-orthogonal D–A molecules based on the Pe–PTZ framework were synthesized to systematically modulate the electronic properties and spatial arrangement between the donor and acceptor units. Structural features were designed to ensure orthogonality between the Pe and PTZ moieties, minimizing ground-state electronic interactions. In particular, the introduction of TPA groups aimed to enhance the electron-donating ability of the donor unit, while a phenyl spacer was introduced to modulate the distance between the donor and acceptor. The synthetic routes involved palladium-catalyzed cross-coupling reactions between bromo-substituted perylene or phenothiazine precursors and appropriate donor or linker units, followed by purification via column chromatography and gel permeation chromatography (Figures S18–S21 in Supporting Information File 1). The details of the syntheses are shown in Supporting Information File 1.

### Energy levels and steady-state optical properties

**Figure 2:**
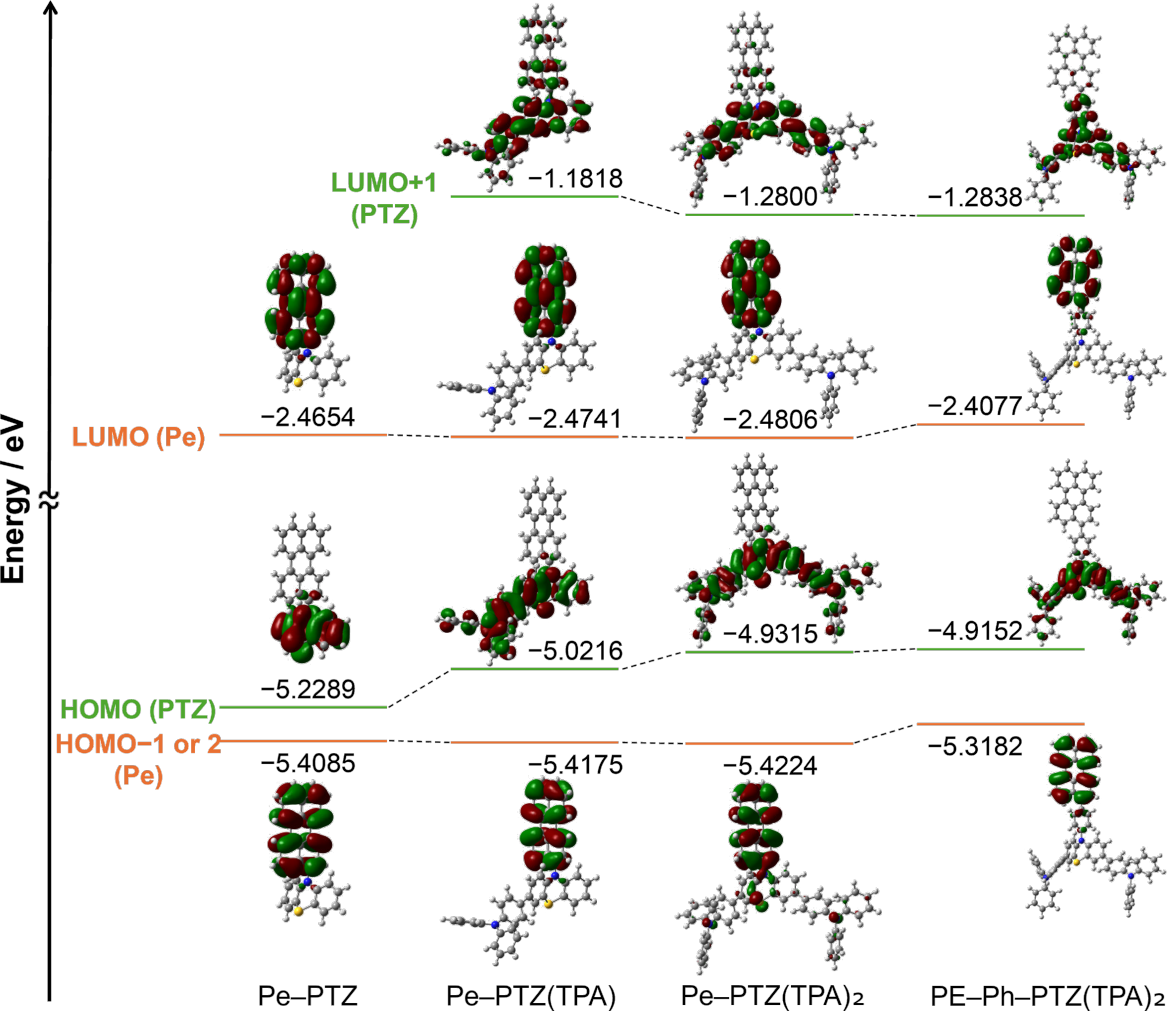
Energy diagrams around the frontier orbitals of Pe–PTZ, Pe–PTZ(TPA), Pe–PTZ(TPA)_2_, and Pe–Ph–PTZ(TPA)_2_ at the B3LYP/6-31+G(d,p)//B3LYP/6-31+G(d,p) level of theory.

Time-dependent density functional theory (TD-DFT) calculations were performed to gain insights into the electronic structures of Pe–PTZ derivatives at B3LYP/6-31+G(d,p) level of theory (Figure 2) [18]. The calculations revealed that the highest occupied molecular orbitals (HOMOs) are localized at the PTZ or PTZ–TPA moieties in all compounds. In contrast, the lowest unoccupied molecular orbitals (LUMOs) are localized at the Pe moiety. This spatial separation of frontier orbitals suggests a weak electronic coupling in the ground state, consistent with the π-orthogonal molecular design. The introduction of electron-donating TPA groups raises the HOMO energy levels, while the LUMO levels remain relatively unaffected, thereby reducing the HOMO–LUMO gaps. The presence of phenyl spacers in Pe–Ph–PTZ(TPA)_2_ lowers the degree of electronic interaction between the donor PTZ and acceptor Pe moieties, as indicated by the decreased overlap between HOMO and LUMO.

**Figure 3:**
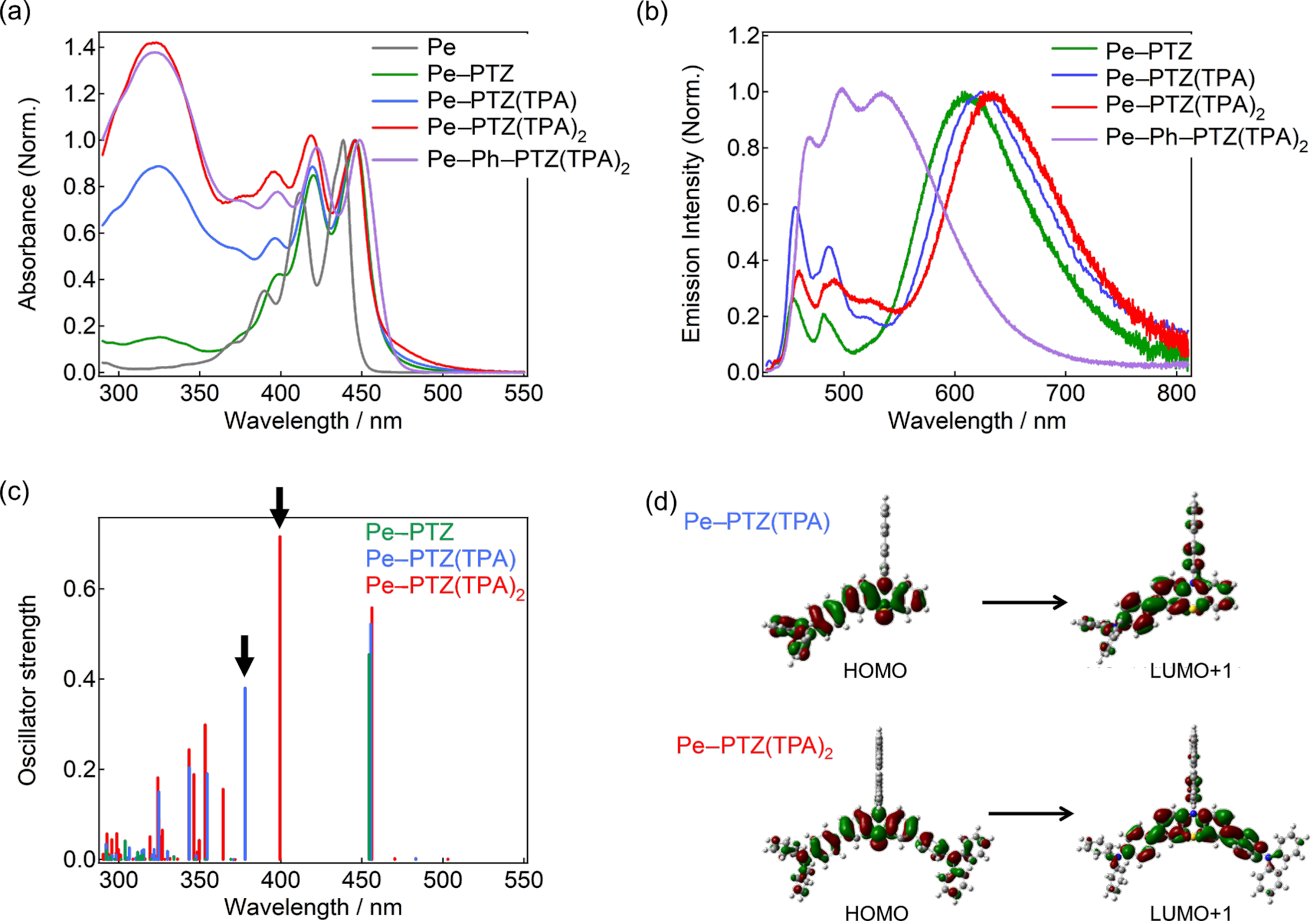
Steady-state (a) absorption and (b) emission spectra of Pe–PTZ, Pe–PTZ(TPA), Pe–PTZ(TPA)_2_, and Pe–Ph–PTZ(TPA)_2_ in benzene at room temperature. The excitation wavelength was 420 nm for Pe–PTZ, Pe–PTZ(TPA)_2_, and Pe–Ph–PTZ(TPA)_2_, and 415 nm for Pe–PTZ(TPA). (c) Simulated absorption spectra of these compounds (B3LYP/6-31+G(d,p)//B3LYP/6-31+G(d,p) level of theory), and (d) molecular orbitals mainly contributing to the optical transitions indicated by arrows.

UV–vis absorption spectra of the Pe–PTZ derivatives were recorded in benzene at room temperature (Figure 3a). All compounds exhibited characteristic absorption bands derived from the Pe moiety between 390 and 450 nm. A broad absorption feature around 320 nm, whose intensity increased with the number of TPA groups, was also observed. The increase in the absorption with the increase in the number of TPA groups is reproduced by TD-DFT calculations (Figure 3c, indicated by arrows), although the observed wavelength was slightly shifted to a longer wavelength. The calculations suggest that the absorption around this wavelength is explained by the optical transitions between the molecular orbitals, which are mainly distributed around the PTZ and TPA moieties (Figure 3d, details are described in Figures S35–S38 in Supporting Information File 1). Absorption peaks associated with the Pe moiety are almost identical irrespective of the number of TPA groups, whereas they were slightly shifted to a longer wavelength in Pe–Ph–PTZ(TPA)_2_. This indicates that the absorption originated from the Pe moiety does not depend on the PTZ moieties, most probably due to the nearly orthogonal alignment. On the other hand, the absorption tail at 460–500 nm extended further into the lower energy region following the order Pe–PTZ < Pe–PTZ(TPA) < Pe–PTZ(TPA)_2_. These absorption tails were ascribed to the CT transitions between the PTZ to Pe moieties. Pe–Ph–PTZ(TPA)_2_ exhibited a less pronounced tailing, likely due to the increased spatial separation between the donor and the acceptor by a phenyl spacer, reducing electronic coupling. TD-DFT calculations indicated that, in TPA-substituted compounds, several weak absorption bands corresponding to CT transitions (primarily HOMO→LUMO) appear in the longer-wavelength region (Figure 3c), in good agreement with the experimental observations. Steady-state emission spectra of the Pe–PTZ derivatives displayed dual emission bands: a structured band in the 450–500 nm region associated with the LE state of the Pe moiety, and a broad, red-shifted band in the 550–750 nm region attributed to the CT emission (Figure 3b). The CT emission showed strong solvent dependence, i.e., shifting to longer wavelengths and decreasing in intensity as the solvent polarity increased, which are typical features of CT states (Figure S22 in Supporting Information File 1). The emission intensity of the CT band was almost comparable to that of the LE band of the Pe moiety in Pe–Ph–PTZ(TPA)_2_, whereas the emission intensity of the CT band is larger than that of the LE band of the Pe moiety in other compounds. This suggests that the phenyl spacer effectively suppressed the CT interaction. It is noted that the substitution of the TPA group increases the relative amplitude of the CT emission band. On the other hand, it was found that the intensity does not necessarily depend on the number of TPA groups. The relative emission quantum yields (Φ_f_) in benzene solution were 2.4, 3.1, 2.4, and 20% for Pe–PTZ, Pe–PTZ(TPA), Pe–PTZ(TPA)_2_, and Pe–Ph–PTZ(TPA)_2_, respectively. This order corresponds to the relative intensities of the CT band compared to the LE band of the Pe moiety. This result suggests that a stronger CT character leads to a lower overall fluorescence quantum yield. The Φ_f_ in other solvents are shown in Table S1 (Supporting Information File 1). In addition, a gradual increase in the emission intensity around 500–520 nm is observed with an increasing number of TPA substituents. This observation suggests the presence of an additional emission band originating from the PTA-substituted PTZ unit, in addition to the emissions from the LE state of the Pe moiety and the CT state. The assignment of this band will be discussed in detail based on fluorescence lifetime measurements in the following section.

**Figure 4:**
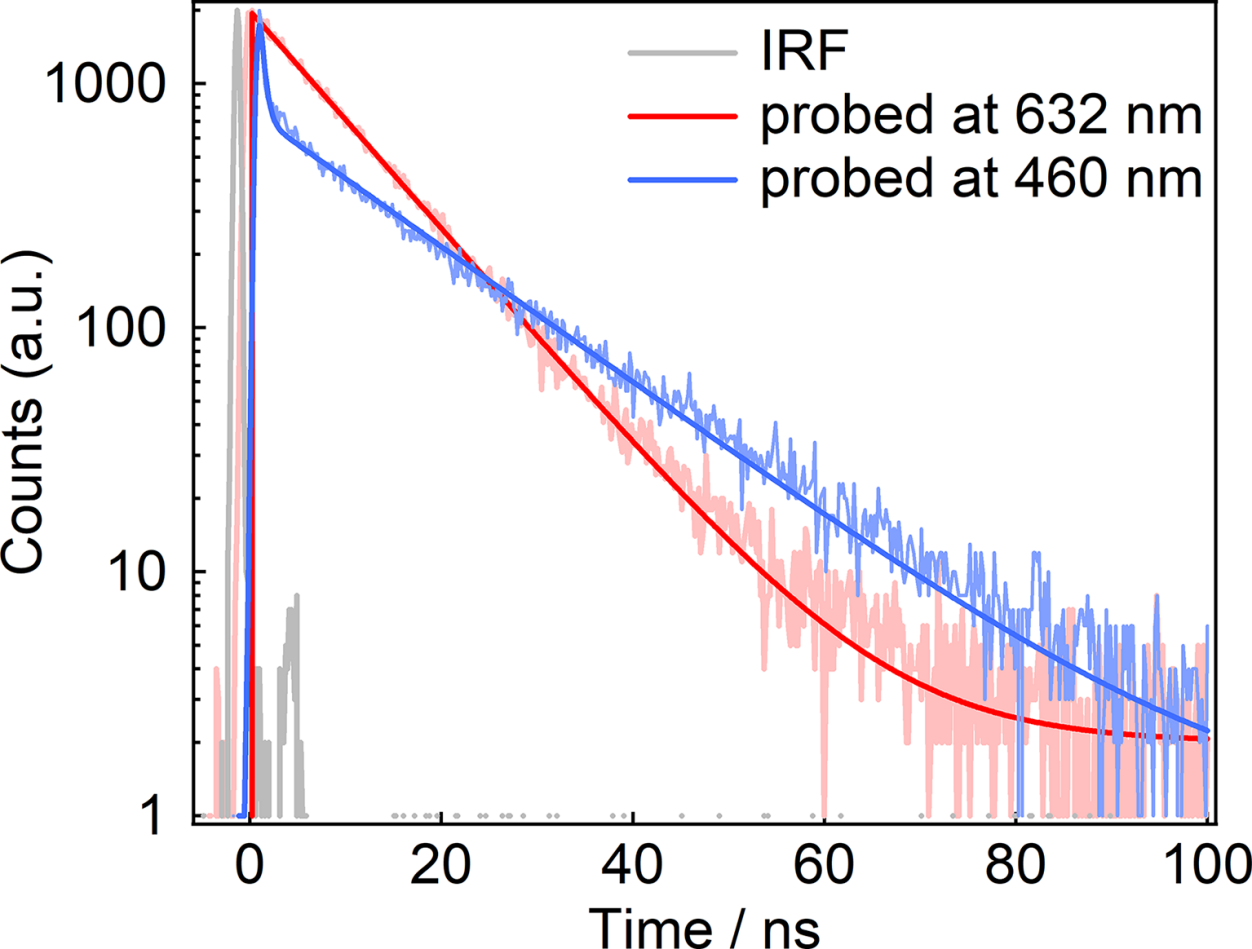
Emission decay curves of Pe–PTZ(TPA)_2_ in benzene excited at 403 nm and probed at 460 and 632 nm.

Since the CT states of these molecules exhibit emissive properties, their excited-state lifetimes were determined from emission decay measurements. Time-resolved fluorescence measurements revealed that the decays of the CT emission band follow a single-exponential decay function in all compounds (Supporting Information File 1, Figure S23 and Figure S24). As a representative example, Figure 4 presents the emission decay profiles of Pe–PTZ(TPA)_2_, with overlaid decay curves monitored at 600 nm and 460 nm. The lifetimes of the CT states were 10.7, 10.3, 9.7, and 10.8 ns for Pe–PTZ, Pe–PTZ(PTA), Pe–PTZ(PTA)_2_, and Pe–Ph–PTZ(PTA)_2_, respectively. At the probe wavelength corresponding to the LE band of the Pe moiety (460 nm), a subnanosecond decay attributable to the LE state of the Pe moiety (within the instrumental response function, IRF) was observed, along with a relatively slower decay component on tens of nanosecond timescales. Notably the lifetime of the slower component (15 ns) was clearly different from that of the CT emission (10 ns). This implies that the slower decay component at 460 nm is different from the CT state observed at the decay at 632 nm. Taking into account the broad emission feature observed at 500–520 nm only in the TPA-substituted PTZ derivatives, the emission component may be assigned to another state derived from TPA moieties.

### Microsecond transient absorption spectroscopy

**Figure 5:**
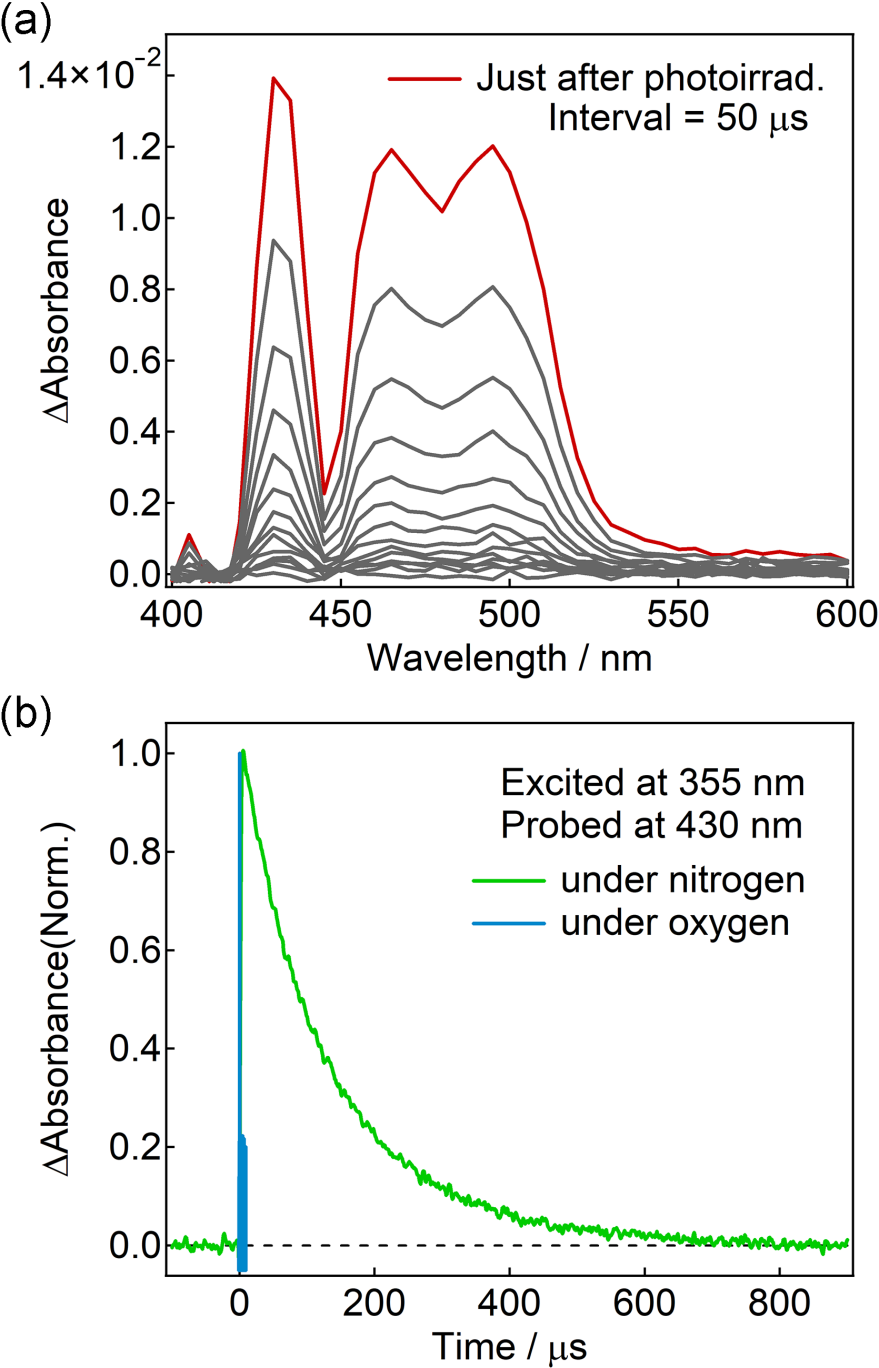
Microsecond transient absorption (a) spectra and (b) dynamics of Pe–PTZ(TPA)_2_ in benzene excited at 355 nm (5 μJ/pulse) and probed at 430 nm under nitrogen and oxygen conditions.

To elucidate the excited-state dynamics of these molecules, transient absorption spectroscopy measurements spanning the femtosecond to microsecond timescales were conducted. When electron transfer occurs between the donor and acceptor, the spin–spin interaction becomes weaker, facilitating the formation of triplet excited states upon recombination. This process is known as spin–orbit charge transfer intersystem crossing (SOCT-ISC), as described in the Introduction. Therefore, the dynamics of the triplet excited state were initially investigated using microsecond transient absorption spectroscopy. Upon excitation at 355 nm, all compounds exhibited transient absorption bands centered between 420 and 550 nm, attributed to the triplet LE (^3^LE) state of the Pe moiety (Figure 5a for Pe–PTZ(TPA)_2_ and the spectra of the other compounds are shown in Figures S25–S27 in Supporting Information File 1). Under nitrogen atmosphere, the triplet signals displayed lifetimes in the order of several microseconds. In contrast, under an oxygen atmosphere, the transient absorption decayed significantly faster, confirming the triplet nature of these states (Figure 5b and Figures S25–S27 in Supporting Information File 1). Under a nitrogen atmosphere, the decay time constants were found to be 77, 46, 135, and 63 μs for Pe–PTZ, Pe–PTZ(TPA), Pe–PTZ(TPA)_2_, and Pe–Ph–PTZ(TPA)_2_, respectively. The transient absorption spectral features closely matched those of the triplet state of perylene, indicating that the ^3^LE state of the Pe moiety is generated in all of these molecules. Pe is a rigid aromatic hydrocarbon, and exhibits significant overlap between the HOMO and LUMO, resulting in a large singlet–triplet energy gap and a low-lying ^3^LE state. These characteristics likely cause the excited states of all compounds to relax preferentially to the ^3^LE state of the Pe moiety.

### Femtosecond-to-nanosecond transient absorption spectroscopy

To gain deeper insights into the effects of the electron-donating TPA substituents on the excited-state dynamics, femtosecond-to-nanosecond transient absorption spectroscopy measurements were carried out. While the excited-state dynamics of Pe–PTZ have been studied in a previous study [15], this study focused first on a detailed analysis of Pe–PTZ(TPA)_2_, followed by a comparison with other derivatives. The transient absorption spectra were analyzed using global analysis based on singular value decomposition with the Glotaran program to resolve into the evolution-associated spectra (EAS) [19]. The spectral evolution over time was tentatively analyzed using a three- or four-component sequential decay model (for example, the four-state kinetic model is described as EAS1 → EAS2 → EAS3 → EAS4 → ground state).

**Figure 6:**
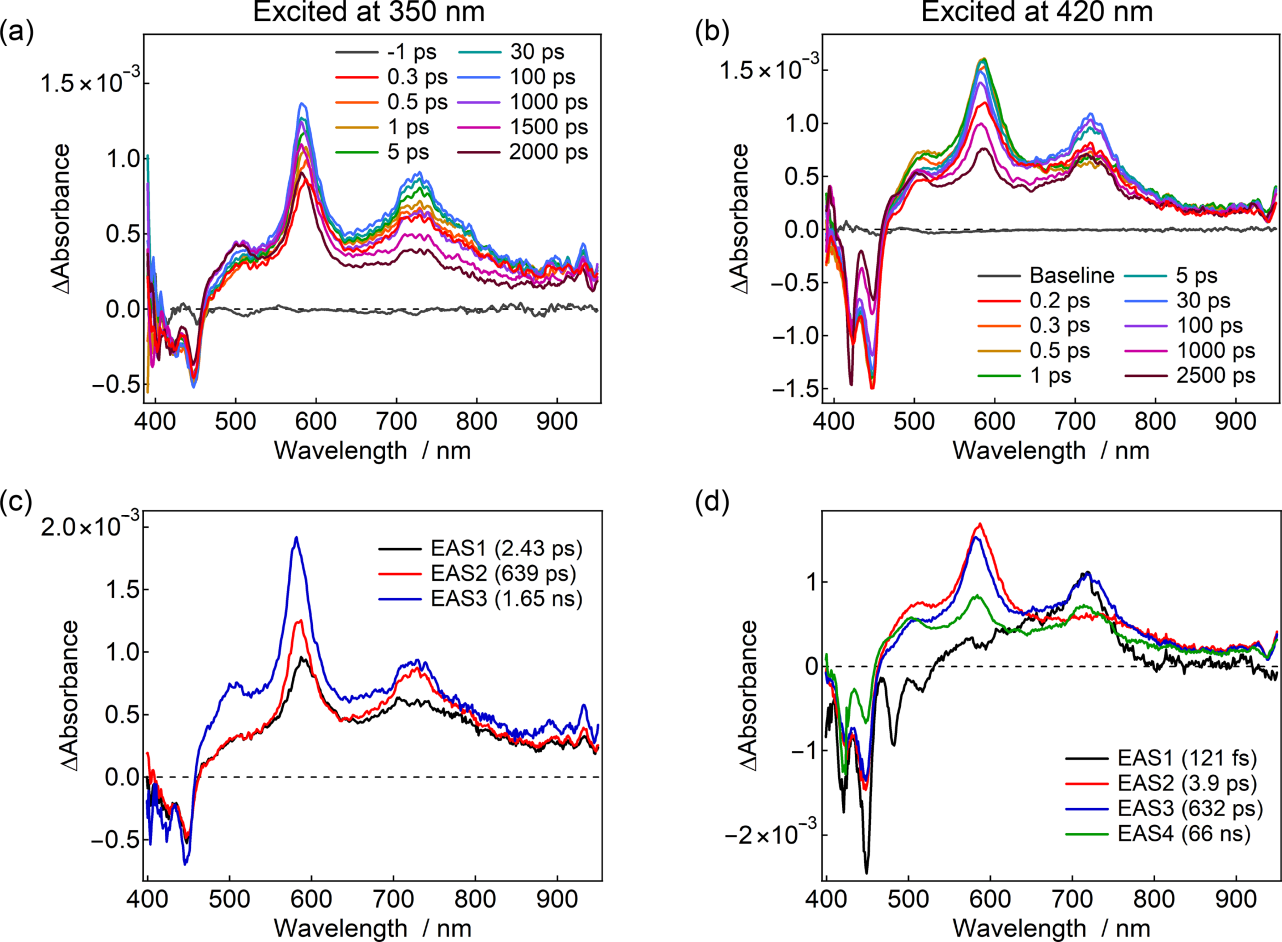
Femtosecond-to-nanosecond transient absorption spectra and evolution-associated spectra (EAS) of Pe–PTZ(TPA)_2_ in benzene excited at (a, c) 350 nm (20 nJ/pulse) and (b, d) 420 nm (6 nJ/pulse) at room temperature.

Figure 6a and 6c show the transient absorption spectra and EAS of Pe–PTZ(TPA)_2_ in benzene excited at 350 nm. The excitation at 350 nm predominantly corresponds to an absorption band enhanced by the introduction of TPA groups, suggesting preferential excitation of the PTZ(TPA)_2_ moiety. Immediately after excitation, ground-state bleach signals at 425 and 445 nm and positive transient absorption bands at 505, 582, and 725 nm were observed (0.3 ps). The overall spectral features were highly similar to those previously reported for Pe–PTZ [15], particularly the characteristic band at 582 nm, which was assigned to the radical anion of the Pe moiety. Additionally, a shoulder signal observed near 500 nm was attributed to the radical cation of the PTZ(TPA) unit. However, although previous studies selectively excited the Pe moiety and attributed the 725 nm band to the LE state of Pe moiety, such an assignment may not be directly applicable to the present case because the PTZ(TPA)_2_ moiety was predominantly excited in the present case.

To clarify the origin of the 725 nm band, a reference compound lacking the Pe moiety (PTZ(TPA)_2_, Figure 1) was synthesized, and its transient absorption spectra were measured. Remarkably, a transient absorption band around 710 nm, similar to the ESA of the LE state of the Pe moiety, was observed (Supporting Information File 1, Figure S29a). This result indicates that the ^1^LE states at both the PTZ(TPA)_2_ and Pe moieties exhibit transient absorption near 710–720 nm, necessitating careful interpretation. Notably, as will be discussed later in Figure 6d, the ^1^LE state of the Pe moiety can be identified by its characteristic stimulated emission in the 490–510 nm region. Based on these observations, it is concluded that selective excitation of the PTZ(TPA)_2_ moiety at 350 nm generates both the ^1^LE state of the PTZ(TPA)_2_ moiety and the Pe radical anion almost instantaneously. The electron transfer occurs extremely rapidly (within the instrumental response function, <100 fs), likely via a direct CT transition. Subsequently, both signals showed growth components with time constants of 2.4 and 639 ps. Although the PTZ unit adopts a bent structure in the ground state, it is known to undergo a structural transformation to a planar conformation upon excitation, as the planar geometry becomes more stable in the excited state. For instance, the planarization time constant of a phenyl-substituted phenothiazine derivative, 12-phenyl-12*H*-benzo[*a*]phenothiazine, in toluene at room temperature has been estimated to be 28 ps (with an additional minor 0.7 ps component attributed to small-scale structural changes) [17]. It is also noted that the relaxation dynamics depend on the molecular structure and the surrounding environment. Considering that Pe–PTZ(TPA)_2_ is significantly larger than that in the aforementioned study, and that benzene has a slightly higher viscosity than toluene (0.60 and 0.56 cP at 297 K, respectively), the time constant of 2.43 ps of the first EAS (EAS1) can be attributed to reorganization of solvent molecules associated with excited-state charge redistribution and minor conformational changes. Meanwhile, the time constant of 639 ps of the second EAS (EAS2) may be ascribed to the planarization of the PTZ(TPA)_2_ moiety.

Several hundred picoseconds after excitation, a new transient absorption band at ≈505 nm emerged, which was assigned to the ^3^LE state of the Pe moiety, as confirmed by microsecond transient absorption measurements. Interestingly, even after the formation of the Pe radical anion via electron transfer, the component corresponding to the ^1^LE state of the PTZ(TPA)_2_ moiety remained observable for nanoseconds or longer. This behavior, not seen in the parent Pe–PTZ, is attributed to the introduction of TPA groups.

Furthermore, when the same measurements were performed in acetonitrile, the signals derived from the LE state disappeared, leaving only the CT-state component (Supporting Information File 1, Figure S29b). Given that the CT states are relatively unstable (i.e., energetically higher-lying) in benzene, and that the LE emission from the PTZ(TPA)_2_ moiety was concurrently observed with the CT emission in the emission decay measurements, it is inferred that a transient equilibrium exists between the ^1^LE state of the PTZ(TPA)_2_ moiety and CT states (see Figure 8 for summarized energy diagrams). The introduction of TPA groups not only stabilizes the CT state but also stabilizes the LE state, bringing their energy levels into proximity and thus creating the observed equilibrium in nonpolar environments.

Subsequently, transient absorption measurements of Pe-PTZ(TPA)_2_ in benzene were performed using a 420 nm excitation pulse, where the Pe moiety is predominantly excited (Figure 6b and 6d). Immediately after excitation, ground-state bleach, stimulated emission, and excited-state absorption signals corresponding to the ^1^LE state of the Pe moiety were observed for a very short duration. Based on the characteristic stimulated emission signals of the Pe moiety at 480–510 nm as shown in Figure 6d, EAS1 can be safely assigned to the ^1^LE state of the Pe moiety. With a time constant of ≈120 fs, a band at 582 nm, attributed to the Pe radical anion, was generated, clearly demonstrating that the time constant of the reductive electron transfer from the PTZ(TPA)_2_ to the Pe moieties is ≈120 fs. Afterward, at 3.9 ps, a broader transient absorption band near 710 nm reappeared, assigned to the thermal equilibrium between the CT state and the LE S_1_ state of the PTZ(TPA)_2_ moiety. These observations indicate that after initial electron transfer, the LE S_1_ state of PTZ(TPA)_2_ moiety and the CT state coexist transiently in a thermal equilibrium. Subsequently, the 500 nm band gradually increased, reflecting the formation of the ^3^LE state of the Pe moiety.

The recombination process after electron transfer was measured using the randomly-interleaved pulse train (RIPT) method (Supporting Information File 1, Figure S28) [20]. The Pe radical anion signal decayed with a time constant of 10 ns, while the 500 nm band attributed to the ^3^LE state of the Pe moiety persisted. The CT-state formation thus facilitated subsequent triplet generation via SOCT-ISC, enabled by the orthogonal arrangement of the donor and acceptor moieties.

When Pe–PTZ(TPA) was excited at 350 nm in benzene, the transient absorption spectra and dynamics observed were generally similar to those of Pe–PTZ(TPA)_2_ (Supporting Information File 1, Figure S29c). However, the broad band between 700–800 nm is split into two peaks at 720 and 790 nm, likely due to asymmetric substitution of a single TPA group. The time constants of photoinduced structural planarization of the PTZ(TPA) moiety were determined to be 6.2 and 470 ps. The acceleration of the planarization of the PTZ(TPA) moiety (470 ps vs 639 ps for PTZ(TPA)_2_) can be attributed to the presence of only one TPA group, which facilitates structural reorganization of the PTZ moiety. Conversely, the deceleration of the early component (6.2 ps vs 2.4 ps for PTZ(TPA)_2_), likely associated with solvent reorientation, may be explained by the larger dipole rearrangement induced by excitation in this asymmetric molecular framework, which in turn requires more time for the reorganization of the surrounding solvent molecules.

**Figure 7:**
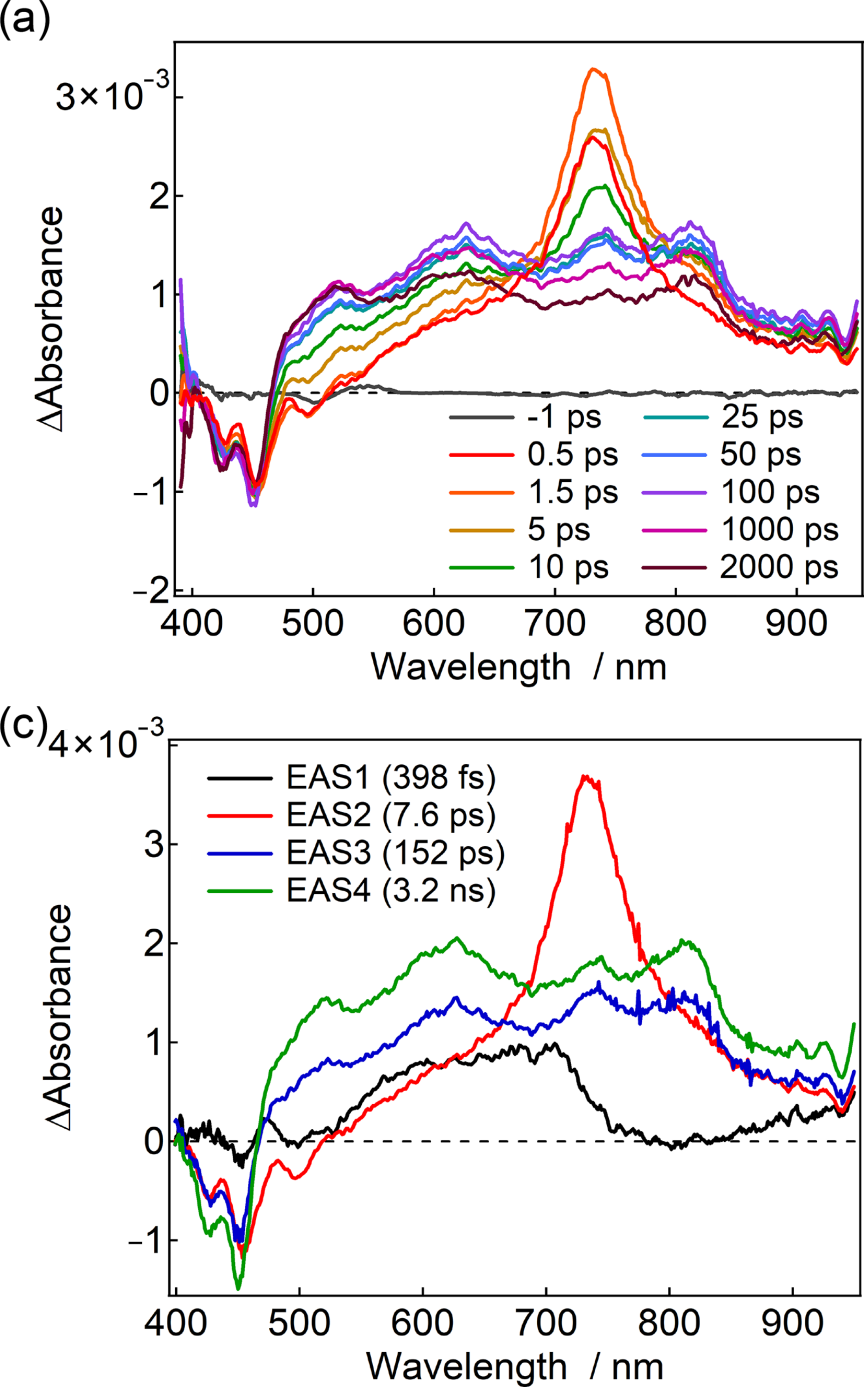
(a) Femtosecond-to-nanosecond transient absorption spectra and (b) evolution-associated spectra (EAS) of Pe–Ph–PTZ(TPA)_2_ in benzene excited at 350 nm (20 nJ/pulse) at room temperature.

For Pe–Ph–PTZ(TPA)_2_, the transient equilibrium behavior, like Pe–PTZ(TPA)_2_, was obscured. Upon excitation at 390 nm, where both PE and PTZ(TPA)_2_ moieties are expected to be excited, the transient absorption band at 720 nm and negative signals at 420–500 nm were instantaneously observed. These negative signals are ascribed to the bleach and stimulated emission of the PE moiety, suggesting that the transient absorption band at 720 nm is ascribed to the excited-state absorption of ^1^LE state of the Pe moiety. These signals gradually decayed, and broad transient absorption features attributable to the Pe radical anion and the PTZ(TPA) radical cation appeared at ≈600 and ≈500 nm, respectively (Figure 7). The electron transfer time constant was determined to be 7.6 ps, significantly slower than that of Pe–PTZ(TPA)_2_ (120 fs or faster), reflecting the effect of the phenyl spacer that increases the D–A distance and thus reduces the electronic coupling.

**Figure 8:**
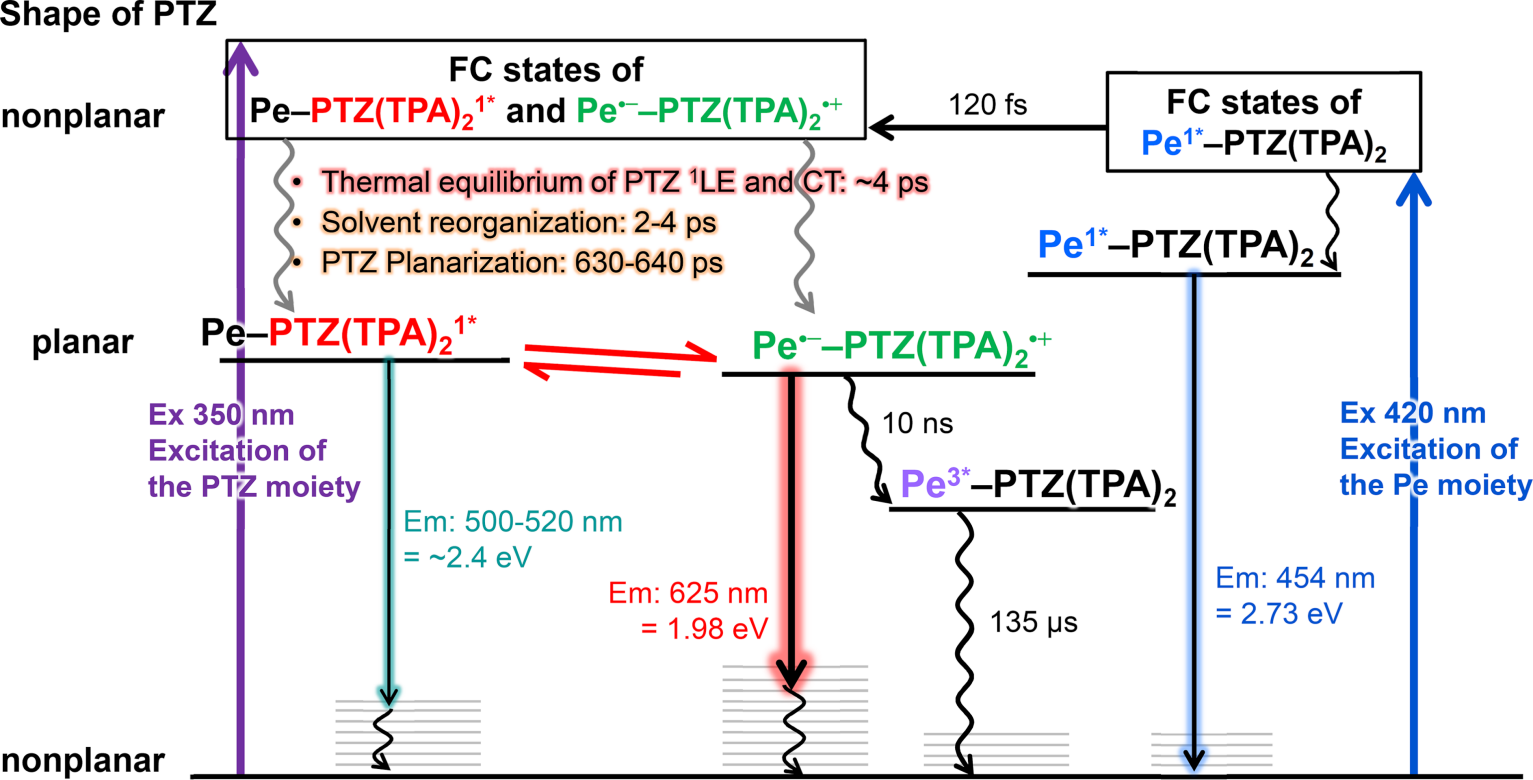
Summarized photophysical process of Pe–PTZ(TPA)_2_ in benzene at room temperature. The left side describes the brief shape of the PTZ moiety upon excitation. Abbreviations of Ex, Em, FC, ISC are excitation, emission, Franck–Condon, and intersystem crossing, respectively.

The photophysical processes of Pe–PTZ(TPA)_2_ are summarized in Figure 8. Upon excitation of the PTZ moiety with a 350 nm pulse, transient absorption signals originating from both the excited PTZ moiety and the perylene radical anion were observed almost instantaneously. As the PTZ moiety relaxes from a nonplanar to a planar structure in the excited state, the transient absorption spectra evolve with time constants of several picoseconds and approximately 630–640 ps. During this process, the signal of the Pe radical anion gradually increases, while the signal attributed to the ^1^LE state of the PTZ moiety remains. This result suggests that the ^1^LE state of the PTZ moiety and the CT state coexist in a transient equilibrium. This equilibrium eventually leads to charge recombination, forming the ^3^LE state of the Pe moiety with a time constant of 10 ns, which subsequently returns to the ground state with a lifetime of 135 μs.

In contrast, when the Pe moiety is predominantly excited using a 420 nm pulse, the ground-state bleach and stimulated emission associated with the ^1^LE state of the Pe moiety are initially observed. However, this state rapidly decays with a time constant of 120 fs, followed by the immediate formation of the CT state. Subsequently, a transient signal attributed to the ^1^LE state of the PTZ moiety appears at 3.9 ps, indicating that the thermal equilibrium between the ^1^LE state of the PTZ moiety and the CT state is established within approximately 4 ps. The later-stage dynamics follow a similar pathway as those observed upon 350 nm excitation, involving planarization of the PTZ moiety and triplet formation via charge recombination.

## Conclusion

This study reveals the unique excited-state dynamics of π-orthogonal donor–acceptor systems, TPA-substituted Pe–PTZ derivatives. Femtosecond-to-microsecond transient absorption spectroscopy demonstrated that Pe–PTZ(TPA) and Pe–PTZ(TPA)_2_ exhibit a transient equilibrium between the ^1^LE state of the PTZ moiety and the photoinduced CT state. Notably, this equilibrium is not primarily driven by the enhanced electron-donating ability of the TPA units, but rather by the stabilization of the PTZ ^1^LE state, which is facilitated by planarization of the PTZ moiety in the excited state and the resulting increase in π-conjugation. This behavior led to the persistent presence of ^1^LE-state signals coexisting with those of the CT state, and ultimately to the formation of the Pe ^3^LE state via charge recombination. Such a dynamic interplay between the LE and CT states was obscured in Pe–Ph–PTZ(TPA)_2_, where the spatial separation between the donor and acceptor units is large, highlighting the critical role of the electronic D–A interaction and the stabilization of the ^1^LE state of the PTZ moiety by excited-state induced planarization. These results highlight the importance of structural relaxation in the excited state for controlling photophysical properties, and demonstrate that even within a simple molecular framework, subtle intramolecular interactions – such as increased conjugation induced by planarization – can have a significant impact on the nature of photoinduced charge separation. Such molecules with finely tunable excited-state dynamics are expected to play an important role in the development of next-generation photoresponsive materials exhibiting complex and multifunctional responses [21].

## Supporting Information

File 1 Experimental and computational details, synthesis and characterization of compounds, additional spectroscopic results, and theoretical calculations.

## Data Availability

Data generated and analyzed during this study is available from the corresponding author upon reasonable request.
