# Supplementary material for: Thermodynamic equilibrium between locally excited and charge transfer states in perylene–phenothiazine dyads
Source: Beilstein J Org Chem. 2025 Aug 5;21:1577–86. doi: 10.3762/bjoc.21.121 (PMC12337995; doi:10.3762/bjoc.21.121)
Supplement: File 1 — Experimental and computational details, synthesis and characterization of compounds, additional spectroscopic results, and theoretical calculations. [file Beilstein_J_Org_Chem-21-1577-s001.pdf]

BEILSTEIN JOURNAL OF ORGANIC CHEMISTRY

Supporting Information

for

Thermodynamic equilibrium between locally excited and charge transfer states in perylene–phenothiazine dyads

Issei Fukunaga, Shunsuke Kobashi, Yuki Nagai, Hiroki Horita, Hiromitsu Maeda and Yoichi Kobayashi

Beilstein J. Org. Chem. **2025,** *21,* 1577–1586. doi:10.3762/bjoc.21.121

Experimental and computational details, synthesis and characterization of compounds, additional spectroscopic results, and theoretical calculations

License and Terms: This is a supporting information file under the terms of the Creative Commons Attribution License (https://creativecommons.org/licenses/by/4.0). Please note that the reuse, redistribution and reproduction in particular requires that the author(s) and source are credited and that individual graphics may be subject to special legal provisions.
The license is subject to the *Beilstein Journal of Organic Chemistry* terms and conditions: (https://www.beilstein-journals.org/bjoc/terms)

Table of contents

1. Experimental setup S2
2. Syntheses of materials S4
3. ^{1}H and ^{13}C NMR spectra S11
4. HR-ESI-TOF-MS spectra S17
5. HPLC chromatograms S19
6. Fluorescence spectra S21
7. Fluorescence lifetimes measurements S23
8. Nanosecond-to-microsecond transient absorption measurements S24
9. Femtosecond-to-picosecond transient absorption measurements S26
10. DFT calculations S31
11. References S90

1. Experimental setup

Materials

All reagents were purchased from Tokyo Chemical Industry (TCI), Wako Co. Ltd., Sigma-Aldrich Co. LLC, and Kanto Chemical Co., Inc. and were used without further purification.

Setups for material synthesis, characterization, and steady-state optical measurements

All reactions were monitored by thin-layer chromatography carried out on 0.2 mm E. Merck silica gel plates (60F-254). Column chromatography was performed on silica gel (silica gel 60N, Kanto Chemical Co., Inc.). MALDI-TOFMS measurements were recorded at AXIMA-CFR+ (Shimadzu). Proton nuclear magnetic resonance (^{1}H NMR) spectra were recorded at 400 MHz by JNM-ECS-400 (JEOL). Proton-decoupled carbon-13 nuclear magnetic resonance (^{13}C NMR) spectra were recorded at 125 MHz by JNM-ECZ-500R (JEOL). High performance liquid chromatography (HPLC) was conducted with a Chromaster (Hitachi High-Technologies) equipped with a normal-phase analytical column (Mightysil RP-18GP II, 25 cm × 4.6 mm, 5 μm particle, Kanto Chemical Co., Inc.) and a linear photodiode array (PDA) detector. Gel permeation chromatography (GPC) was conducted with a recycling preparative HPLC series (Japan Analytical Industry Co., Ltd.) equipped with two GPC columns (JAIGEL-2HR Plus) and a UV detector. $CHCl_3$ was used as an eluent with the flow rate of 10 mL/min. Steady-state absorption spectra were measured with a UV-3600 (Shimadzu). The steady-state fluorescence spectra were measured using an RF-6000 (SHIMADZU) and using a 10-mm quartz cuvette. The relative fluorescence quantum yields were estimated using coumarin-153 in ethanol[1] as a reference. Although the emission spectral ranges of the standard and the sample differ significantly, it was roughly assumed that the detector sensitivity is uniform across different wavelengths. The emission quantum yields in different solvents were tentatively calculated as the sum of both LE and CT emission components.

Nanosecond-to-microsecond transient absorption measurements

Microsecond transient absorption measurements were conducted using a TSP–2000 time-resolved spectrophotometer (Unisoku). The third harmonic (355 nm) of a 10 Hz Q-switched Nd:YAG laser (≈5 ns pulse, Minilite II, Amplitude Japan) was used as the excitation light and the laser pulse was focused to the sample placed in a 10-mm quartz cuvette without a defocusing lens under nitrogen atmosphere. The measurements were performed in benzene solutions placed in a 10-mm quartz cuvette under nitrogen or oxygen conditions at room temperature.

Nanosecond transient absorption measurements were conducted by the randomly-interleaved-pulse-train (RIPT) method. A picosecond laser, PL2210A (EKSPLA, 1 kHz, 25 ps, 3.6 μJ/pulse for 355 nm), and a supercontinuum (SC) radiation source (SC-450, Fianium, 20 MHz, pulse width: 50–100 ps depending on the wavelength, 450–2000 nm), were employed as the pump–pulse and probe sources,

respectively. The wavelength of the excitation pulse was set to 355 nm. The measurements were performed in a benzene solution placed in a 2-mm quartz cell under argon with stirring at room temperature.

Femtosecond-to-nanosecond transient absorption measurements

Transient absorption measurements on the femtosecond-to-nanosecond time scale were conducted by a homemade pump-probe system. An amplified femtosecond laser, Spirit One 1040-8 (Spectra-Physics, 1040 nm, the pulse width: ≈270 fs), was split into two beams with a ratio of 1:9. The stronger beam was directed to a noncollinear optical parametric amplifier (NOPA), Spirit-NOPA-3H (Spectra-Physics) to generate the 350, 390, and 420 nm femtosecond laser pulse for the pump beam. The pump beam was chopped prior to the sample at 500 Hz for signal differencing. The other weaker beam was focused to deuterated water placed in a 10-mm quartz cuvette to generate the white light continuum for the probe beam. Both pump and probe beams were focused to the sample solution placed in the 2-mm quartz cuvette. The polarization between the pump and probe pulses was set at magic angle. The transmitted probe beam was detected with a multichannel detection system, PK120-C-RK (UNISOKU), composed of a CMOS linear image sensor and a polychromator. The obtained spectra were calibrated for group velocity dispersion using the data obtained by the optical Kerr signal of CH_2Cl_2 between the pump pulse and the white-light continuum. The instrumental response function was shorter than approximately 100 fs. The measurements were performed at room temperature.

Fluorescence lifetime measurements

Emission lifetimes were measured using a C7990S system (Hamamatsu Photonics) equipped with a 403-nm excitation laser with a repetition rate of 100 kHz. The full width at half maximum (FWHM) of the instrumental response function (IRF) was 73 ps measured by silica nanoparticles dispersed in water.

2. Syntheses

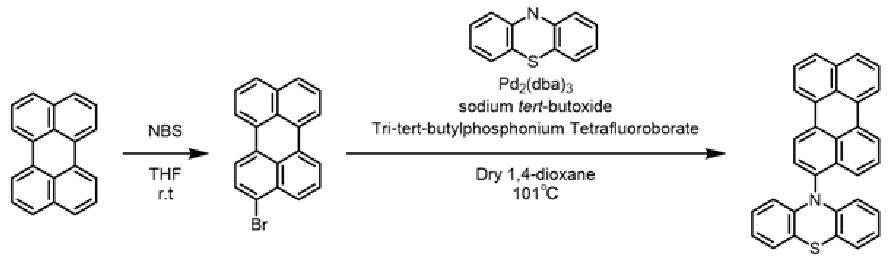

1 Pe-PTZ

3-Bromoperylene (1) in a manner similar to [2]

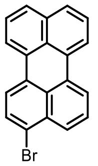

1

A Schlenk flask was charged with perylene (1501.8 mg, 5.957 mmol) in 30 mL THF. *N*-Bromosuccinimide (1064.8 mg, 5.983 mmol) was dissolved in 30 mL THF and dropped to the Schlenk flask over 1 hour. The solution was stirred at room temperature. After stirring for 20 h, the reaction mixture was diluted with H_2O. The mixture was transferred to a separation funnel and extracted with dichloromethane. The organic layers were washed with water and brine and passed through a phase separator paper. After removal of the solvent in vacuo, it was used in the next reaction without purification. MALDI-TOFMS *m/z*: $[M + H]^+$ Calcd for $C_{20}H_{12}Br$ 331; Found 331.

10-(Perylene-3-yl)-10*H*-phenothiazine (Pe–PTZ) in a manner similar to [3]

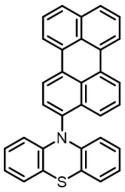

Pe–PTZ

A Schlenk flask was charged with **1** (333.1 mg, 1.00 mmol), phenothiazine (230.0 mg, 1.15 mmol), Pd(dba)$_3$ (19.3 mg, 0.021 mmol), sodium *tert*-butoxide (486.6 mg, 5.06 mmol) and tri-*tert*-butylphosphonium tetrafluoroborate (20.0 mg, 0.069 mmol) in 16 mL of dry 1,4-dioxane. The reaction mixture was stirred at 101 °C for 11 h under reflux. After cooling to room temperature, the reaction mixture was diluted with H_2O. After Celite filtration, the filtrate was transferred to a separation funnel and extracted with dichloromethane. The organic layers were washed with water and brine and passed through a phase separator paper. After removal of the solvent in vacuo, the crude mixture was purified by silica gel column chromatography (dichloromethane/hexane = 1/4) and GPC. ^{1}H NMR (400 MHz, DMSO-d_6): δ 8.62 (d, J = 8.2 Hz, 1H), 8.51-8.44 (m, 3H), 7.91-7.77 (m, 4H), 7.66-7.57 (m, 3H), 7.11-7.08 (m, 2H), 6.87-6.82 (m, 4H), 6.19-6.15 (m, 2H). HRMS (ESI-TOF) *m/z*: $[M]^+$ Calcd for $C_{32}H_{19}NS$ 449.1233; Found 449.1231.

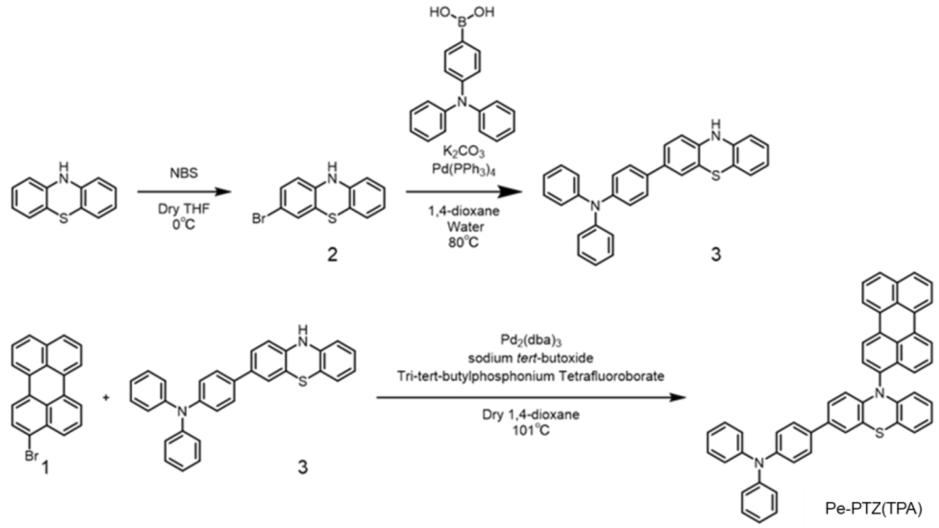

3-Bromo-10*H*-phenothiazine (2) in a manner similar to [4]

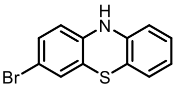

2

A Schlenk flask was charged with phenothiazine (2006.7 mg, 10.0 mmol) in 25 mL THF. *N*-Bromosuccinimide (1789.4 mg, 10.1 mol) was dissolved in 25 mL THF and dropped to the Schlenk flask over 1 hour. The solution was stirred in an ice bath at 0 °C for 1 hour and the mixture was warmed to room temperature. After stirring for 1 h, the reaction mixture was diluted with ethyl acetate and washed with aq. Na_2SO_3. After removal of the solvent in vacuo, the crude mixture was purified by silica gel column chromatography (ethyl acetate/hexane = 1/8). ^{1}H NMR (400 MHz, DMSO-d_6): δ 8.73 (s, 1H), 7.15-7.11 (m, 2H), 7.00 (td, *J* = 7.7, 1.4 Hz, 1H), 6.91 (d, *J* = 6.0 Hz, 1H), 6.77 (t, *J* = 7.0 Hz, 1H), 6.66 (d, *J* = 7.9 Hz, 1H), 6.60 (d, *J* = 8.2 Hz, 1H). MALDI-TOFMS *m/z*: $[M + H]^+$ Calcd for $C_{12}H_8BrNS$ 279; Found 279.

4-(10*H*-Phenothiazine-3-yl)-*N*,*N*-diphenylaniline (3) in a manner similar to [5]

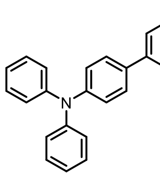

3

A Schlenk flask was charged with **2** (44.7 mg, 0.161 mmol), 4-(diphenylamino)phenylboronic acid (57.8 mg, 0.200 mmol), $Pd(PPh_3)_4$ (39.4 mg, 0.0341 mmol) and potassium carbonate (101.6 mg, 0.736 mmol) in the solvent pair (2 mL of H_2O and 10 mL of 1,4-dioxane). The reaction mixture was stirred at 80 °C for 16 h under reflux. After Celite filtration, the filtrate was transferred to a separation funnel and extracted with ethyl acetate. The organic layers were washed with water and brine and passed through a phase separator paper. After removal of the solvent in vacuo, the crude mixture was purified by silica gel column chromatography (dichloromethane/hexane = 1/1). ^{1}H NMR (400 MHz, DMSO-d_6): δ 8.69 (s, 1H), 7.50 (d, *J* = 8.5 Hz, 2H), 7.33-7.26 (m, 5H), 7.19 (s, 1H), 7.06-6.97 (m, 9H), 6.92 (d, *J* = 7.9 Hz, 1H), 6.79-6.65 (m, 3H). MALDI-TOFMS *m/z*: $[M + H]^+$ Calcd for $C_{30}H_{23}N_2S$ 443; Found 443.

4-(10-(Perylene-3-yl)-10*H*-phenothiazine-3-yl)-*N*,*N*-diphenylaniline (Pe–PTZ(TPA))

A Schlenk flask was charged with **1** (30.3 mg, 0.0918 mmol), **3** (30.2 mg, 0.0683 mmol), Pd(dba)$_3$ (16.8 mg, 0.0183 mmol), sodium *tert*-butoxide (32.4 mg, 0.337 mmol) and tri-*tert*-butylphosphonium tetrafluoroborate (4.0 mg, 0.0138 mmol) in 5 mL of dry 1,4-dioxane. The reaction mixture was stirred at 101 °C for 7 h under reflux. After cooling to room temperature, the reaction mixture was diluted with H_2O. After Celite filtration, the filtrate was transferred to a separation funnel and extracted with dichloromethane. The organic layers were washed with water and brine and passed through a phase separator paper. After removal of the solvent in vacuo, the crude mixture was purified by silica gel column chromatography (dichloromethane/hexane = 1/3) and GPC. ^{1}H NMR (400 MHz, DMSO-*d*$_6$): δ 8.64 (d, *J* = 8.2 Hz, 1H), 8.53-8.45 (m, 3H), 7.91-7.79 (m, 4H), 7.66-7.58 (m, 3H), 7.48 (d, *J* = 8.6 Hz, 2H), 7.36 (d, *J* = 2.3 Hz, 1H), 7.30 (t, *J* = 7.9 Hz, 4H), 7.12 (d, *J* = 9.1 Hz, 2H), 7.04 (q, *J* = 7.2 Hz, 6H), 6.97 (d, *J* = 9.1 Hz, 2H), 6.88-6.82 (m, 2H), 6.19-6.14 (m, 2H). ^{13}C NMR (125 MHz, benzene-*d*$_6$): δ 148.30, 147.33, 144.05, 143.08, 129.63, 136.97, 135.73, 135.17, 134.49, 133.23, 132.63, 132.53, 131.31, 131.05, 130.18, 129.63, 129.05, 128.83, 128.52, 127.52, 127.47, 127.09, 127.01, 126.87, 125.57, 125.15, 124.74, 124.69, 123.84, 123.11, 123.01, 121.46, 121.44, 121.42, 121.18, 120.68, 119.95, 116.65, 116.31 (One carbon signal is missing, probably owing to overlapping with the solvent signal). HRMS (ESI-TOF) *m/z*: [M]$^+$ Calcd for $C_{50}H_{32}N_2S$ 692.2281; Found 692.2284.

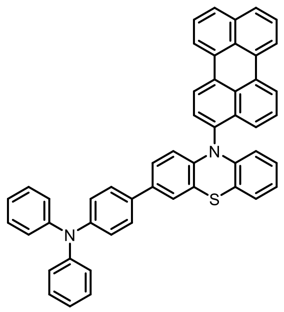

Pe–PTZ(TPA)

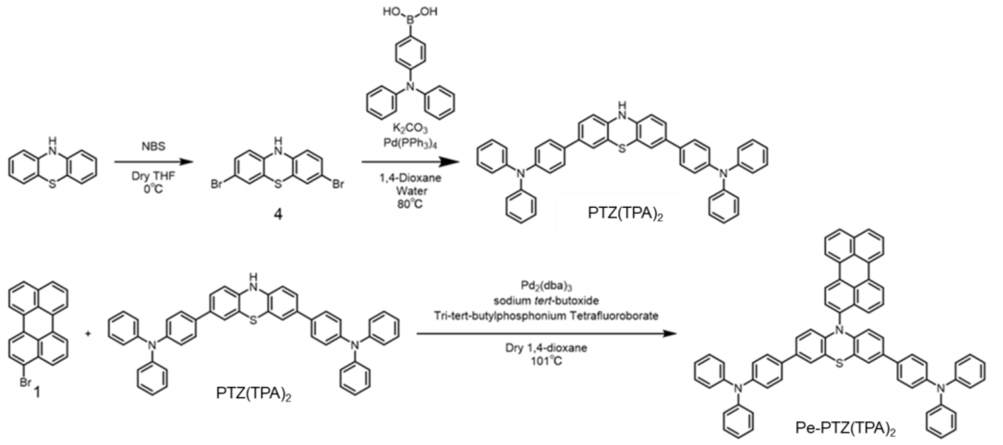

3,7-Dibromo-10*H*-phenothiazine (4) in a manner similar to [6]

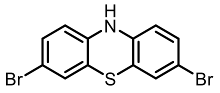

4

A Schlenk flask was charged with phenothiazine (569 mg, 2.82 mmol) in 40 mL THF. *N*-Bromosuccinimide (1136 mg, 6.38 mol) was dissolved in 50 mL THF and dropped to the Schlenk flask over 1 hour. The solution was stirred in an ice bath at 0 °C for 1 hour and the mixture was warmed to room temperature. After stirring for 12 h, the reaction mixture was diluted with ethyl acetate and washed with Na_2SO_3 aq. The solvent was removed by evaporation and was purified by silica gel column chromatography (ethyl acetate/hexane = 1/6). ^{1}H NMR (400 MHz, DMSO-d_6): δ 8.85 (s, 1H), 7.16-7.13 (m, 4H), 6.58 (d, *J* = 8.6 Hz, 2H). MALDI-TOFMS *m/z*: $[M + H]^+$ Calcd for $C_{12}H_7Br_2NS$ 357; Found 357.

4,4'-(10*H*-Phenothiazine-3,7-diyl)bis(*N,N*-diphenylaniline) ($PTZ(TPA)_2$) in a manner similar to [5]

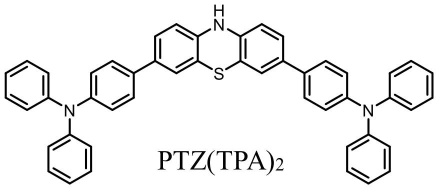

$PTZ(TPA)_2$

A Schlenk flask was charged with **4** (200.0 mg, 0.563 mmol), 4-(diphenylamino)phenylboronic acid (408.5 mg, 1.41 mmol), $Pd(PPh_3)_4$ (236.2 mg, 0.204 mmol) and potassium carbonate (329.0 mg, 2.38 mmol) in the solvent pair (10 mL of H_2O and 40 mL of 1,4-dioxane). The reaction mixture was stirred for 16 h under reflux. After Celite filtration, the filtrate was transferred to a separation funnel and extracted with ethyl acetate. The organic layers were washed with water and brine and passed through a phase separator paper. After removal of the solvent in vacuo, the crude mixture was purified by silica gel column chromatography (ethyl acetate/hexane = 1/5). ^{1}H NMR (400 MHz, DMSO-d_6): δ 8.80 (s, 1H), 7.51 (d, *J* = 9.1 Hz, 4H), 7.30 (q, *J* = 7.9 Hz, 10H), 7.21 (s, 2H), 7.07-6.98 (m, 16H), 6.73 (d, *J* = 8.5 Hz, 2H). HRMS (ESI-TOF) *m/z*: $[M]^+$ Calcd for $C_{48}H_{35}N_3S$ 685.2546; Found 685.2545.

4,4'-(10-(Perylene-3-yl)-10*H*-phenothiazine-3,7-diyl)bis(*N*,*N*-diphenylaniline) (Pe–PTZ(TPA)$_2$)

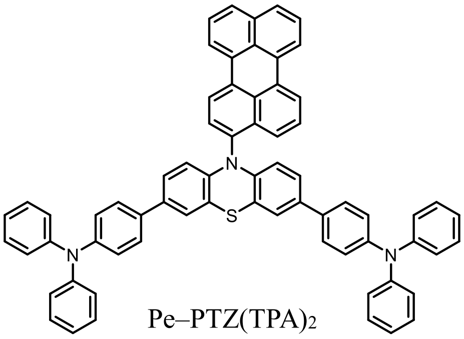

Pe–PTZ(TPA)$_2$

A Schlenk flask was charged with **1** (25.6 mg, 0.0780 mmol), **5** (52.3 mg, 0.0763 mmol), Pd(dba)$_3$ (10.9 mg, 0.011 mmol), sodium *tert*-butoxide (34.7 mg, 0.360 mmol) and tri-*tert*-butylphosphonium tetrafluoroborate (1.2 mg, 0.004 mmol) in 5 mL of dry 1,4-dioxane. The reaction mixture was stirred at 101 °C for 7 h under reflux. After cooling to room temperature, the reaction mixture was diluted with H_2O. After Celite filtration, the filtrate was transferred to a separation funnel and extracted with dichloromethane. The organic layers were washed with water and brine and passed through a phase separator paper. After removal of the solvent in vacuo, the crude mixture was purified by silica gel column chromatography (dichloromethane/hexane = 1/3) and by GPC. ^{1}H NMR (400 MHz, DMSO-d_6): δ 8.65 (d, *J* = 8.5 Hz, 1H), 8.54-8.46 (m, 3H), 7.92-7.81 (m, 4H), 7.66-7.60 (m, 3H), 7.49 (d, *J* = 9.1 Hz, 4H), 7.39 (s, 2H), 7.30 (t, *J* = 7.9 Hz, 8H), 7.12 (dd, *J* = 8.8, 2.1 Hz, 2H), 7.06-6.96 (m, 16H), 6.16 (d, *J* = 8.5 Hz, 2H). ^{13}C NMR (125 MHz, benzene-d_6): δ 148.29, 147.36, 142.85, 136.94, 135.73, 135.17, 134.44, 133.21, 132.70, 132.63, 131.28, 131.08, 131.02, 130.17, 129.64, 129.05, 128.90, 128.58, 127.48, 127.04, 126.91, 125.62, 125.17, 124.74, 124.71, 123.82, 123.14, 121.49 (probably three overlapping aromatic carbon signals), 121.24, 120.41, 116.67 (One carbon signal is missing, probably owing to overlapping with the solvent signal). HRMS (ESI-TOF) *m/z*: [M]$^+$ Calcd for $C_{68}H_{45}N_3S$ 935.3329; Found 935.3331.

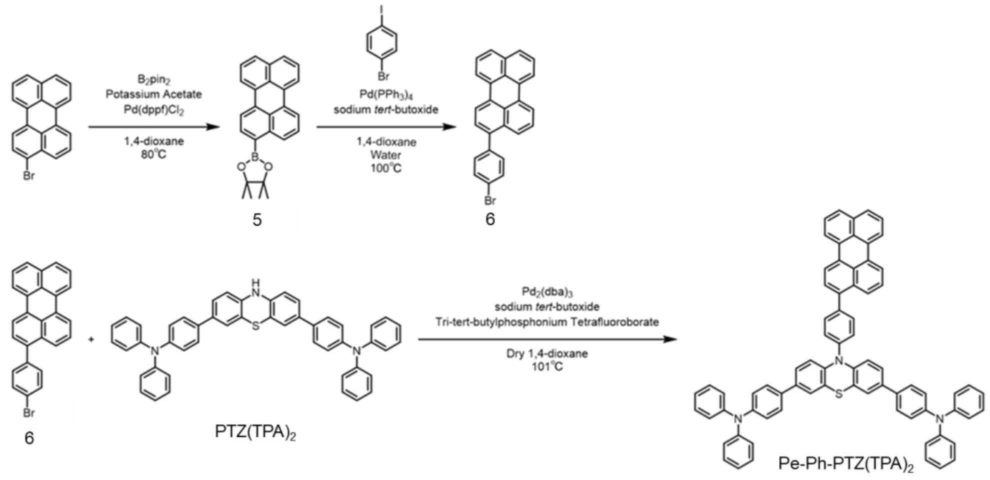

4,4,5,5-Tetramethyl-2-(perylene-3-yl)-1,3,2-dioxaborolane (5) in a manner similar to [7]

A Schlenk flask was charged with **1** (200.6 mg, 0.607 mmol), bis(pinacolato)diboron (240.8 mg, 0.948 mmol), Pd(dppf)Cl_2 (73.1 mg, 0.090 mmol) and potassium acetate (183.7 mg, 1.87 mmol) in 30 mL of 1,4-dioxane. The reaction mixture was stirred at 101 °C for 16 h under reflux. After Celite filtration, the filtrate was transferred to a separation funnel and extracted with dichloromethane. The organic layers were washed with water and brine and passed through a phase separator paper. After removal of the solvent in vacuo, the crude mixture was purified by silica gel column chromatography (dichloromethane /hexane = 1/1). ^{1}H NMR (400 MHz, DMSO-d_6): δ 8.55 (d, J = 8.5 Hz, 1H), 8.42-8.36 (m, 4H), 7.98 (d, J = 7.3 Hz, 1H), 7.83 (dd, J = 13.0, 8.2 Hz, 2H), 7.63-7.55 (m, 3H), 1.39 (s, 12H). MALDI-TOFMS *m/z*: $[M]^+$ Calcd for $C_{26}H_{23}BO_2$ 378; Found 378.

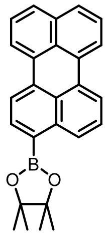

5

3-(4-Bromophenyl)perylene (6) in a manner similar to [8]

A Schlenk flask was charged with **5** (70.3 mg, 0.186 mmol), 1-bromo-4-iodobenzene (81.4 mg, 0.288 mmol), $Pd(PPh_3)_4$ (30.9 mg, 0.0267 mmol) and sodium *tert*-butoxide (61.9 mg, 0.644 mmol) in the solvent pair (5 mL of H_2O and 20 mL of 1,4-dioxane). The reaction mixture was stirred for 22 h under reflux. After Celite filtration, the filtrate was transferred to a separation funnel and extracted with dichloromethane. The organic layers were washed with water and brine and passed through a phase separator paper. After removal of the solvent in vacuo, the crude mixture was purified by silica gel column chromatography (dichloromethane/hexane = 1/5) and by GPC. ^{1}H NMR (400 MHz, DMSO-d_6): δ 8.43 (t, J = 7.9 Hz, 4H), 7.83 (d, J = 7.9 Hz, 2H), 7.75 (d, J = 8.5 Hz, 2H), 7.65 (d, J = 7.9 Hz, 1H), 7.59-7.46 (m, 6H). MALDI-TOFMS *m/z*: $[M + H]^+$ Calcd for $C_{26}H_{16}Br$ 407; Found 407.

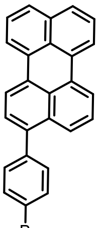

6

4,4'-(10-(4-(Perylene-3-yl)phenyl)-10*H*-phenothiazine-3,7-diyl)bis(*N*,*N*-diphenylaniline) (Pe–Ph–PTZ(TPA)$_2$)

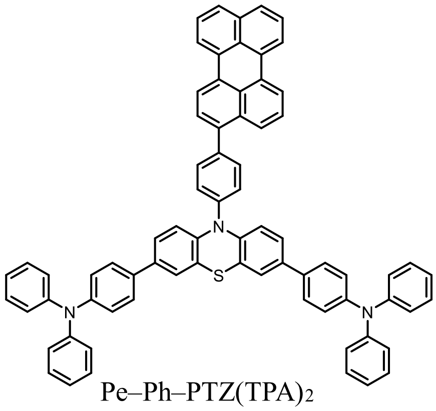

Pe–Ph–PTZ(TPA)$_2$

A Schlenk flask was charged with **6** (25.0 mg, 0.0613 mmol), **PTZ(TPA)$_2$** (43.0 mg, 0.0627 mmol), Pd(dba)$_3$ (15.0 mg, 0.0164 mmol), sodium *tert*-butoxide (30.8 mg, 0.320 mmol) and tri-*tert*-butylphosphonium tetrafluoroborate (11.4 mg, 0.0393 mmol) in 6 mL of dry 1,4-dioxane. The reaction mixture was stirred at 101 °C for 7 h under reflux. After cooling to room temperature, the reaction mixture was diluted with H_2O. After Celite filtration, the filtrate was transferred to a separation funnel and extracted with dichloromethane. The organic layers were washed with water and brine and passed through a phase separator paper. After removal of the solvent in vacuo, the crude mixture was purified by silica gel column chromatography (dichloromethane/hexane = 1/3) and by GPC. ^{1}H NMR (400 MHz, DMSO-d_6): δ 8.51-8.43 (m, 4H), 7.89-7.83 (m, 5H), 7.67-7.54 (m, 10H), 7.42 (d, *J* = 2.4 Hz, 2H), 7.32 (t, *J* = 7.9 Hz, 10H), 7.08-7.00 (m, 16H), 6.41 (d, *J* = 8.5 Hz, 2H). HRMS (ESI-TOF) *m/z*: [M]$^+$ Calcd for $C_{74}H_{49}N_3S$ 1011.3642; Found 1011.3644.

3. ^{1}H and ^{13}C NMR spectra

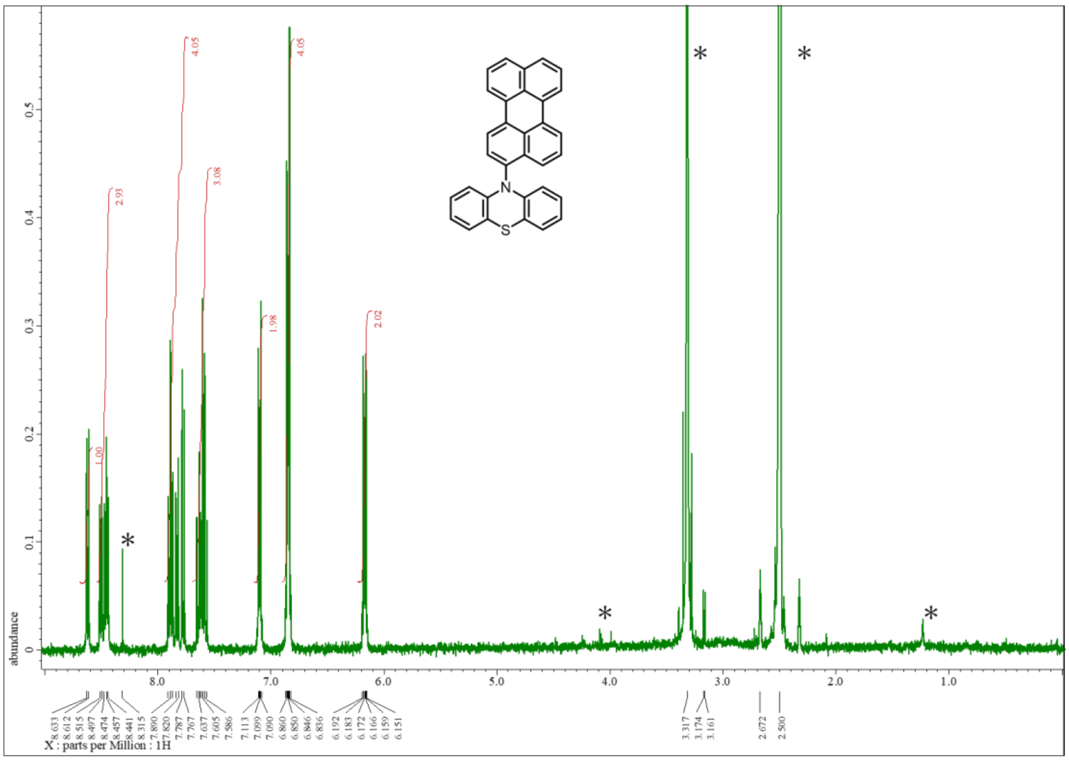

Figure S1. ^{1}H NMR spectrum of Pe–PTZ in DMSO-d_6 (* solvent peaks).

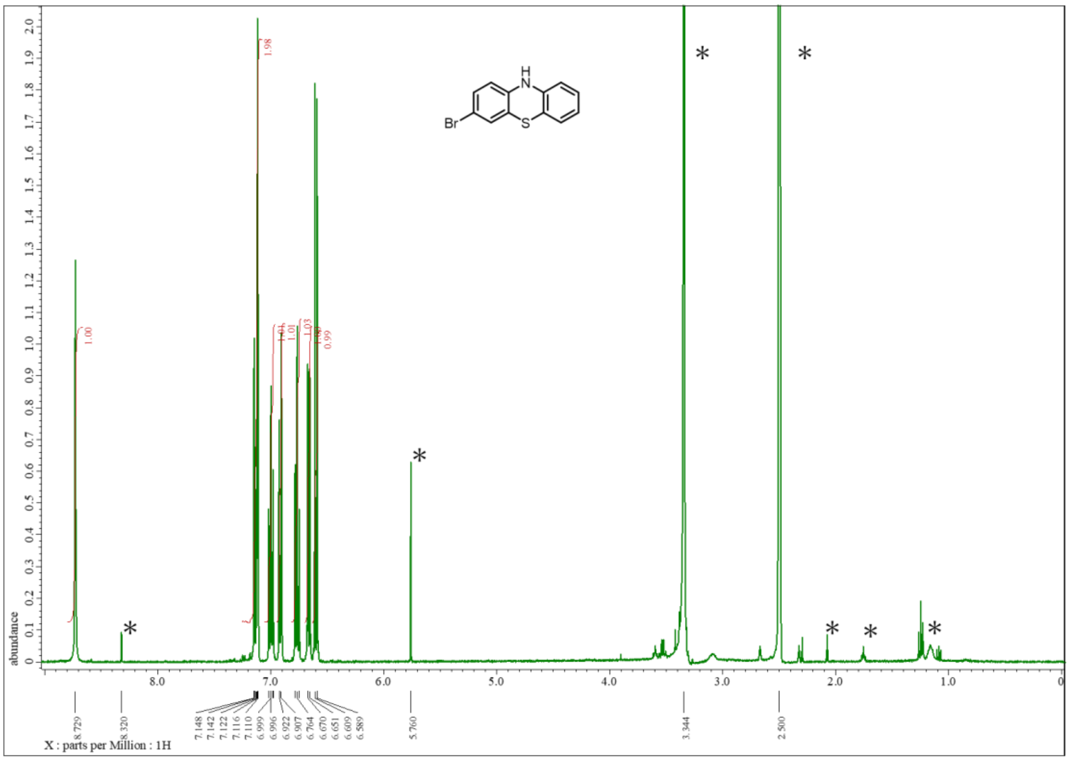

Figure S2. ^{1}H NMR spectrum of **2** in DMSO-d_6 (* solvent peaks).

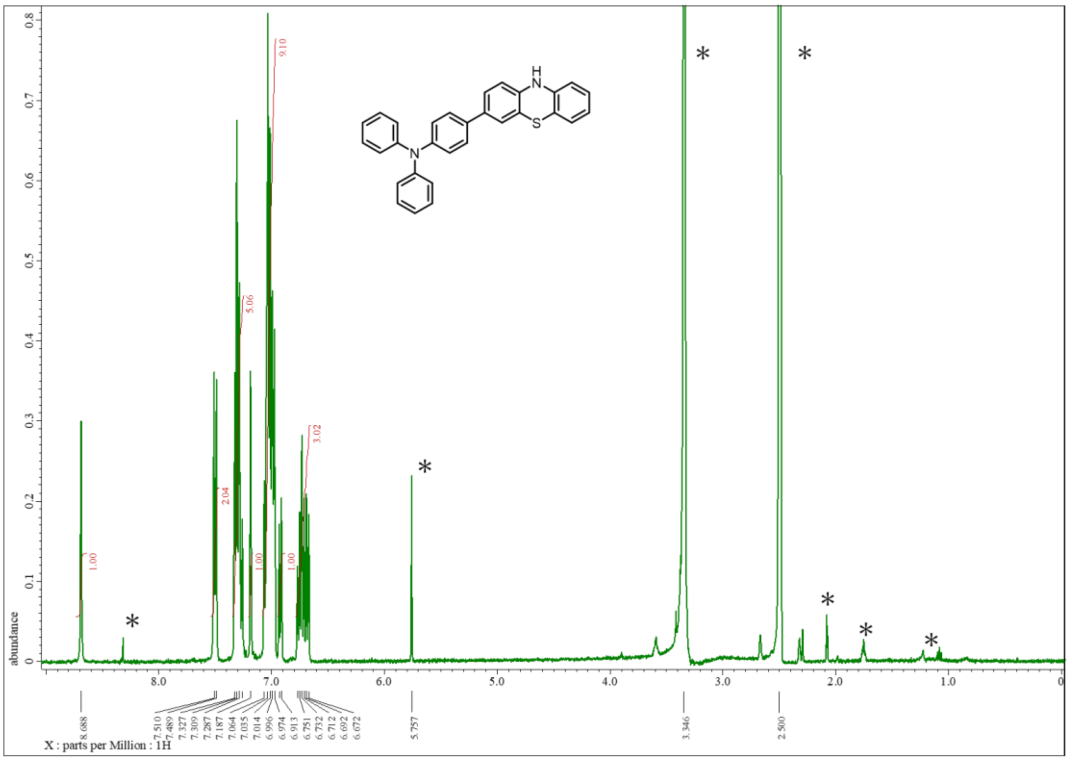

Figure S3. ^{1}H NMR spectrum of **3** in DMSO-d_6 (* solvent peaks).

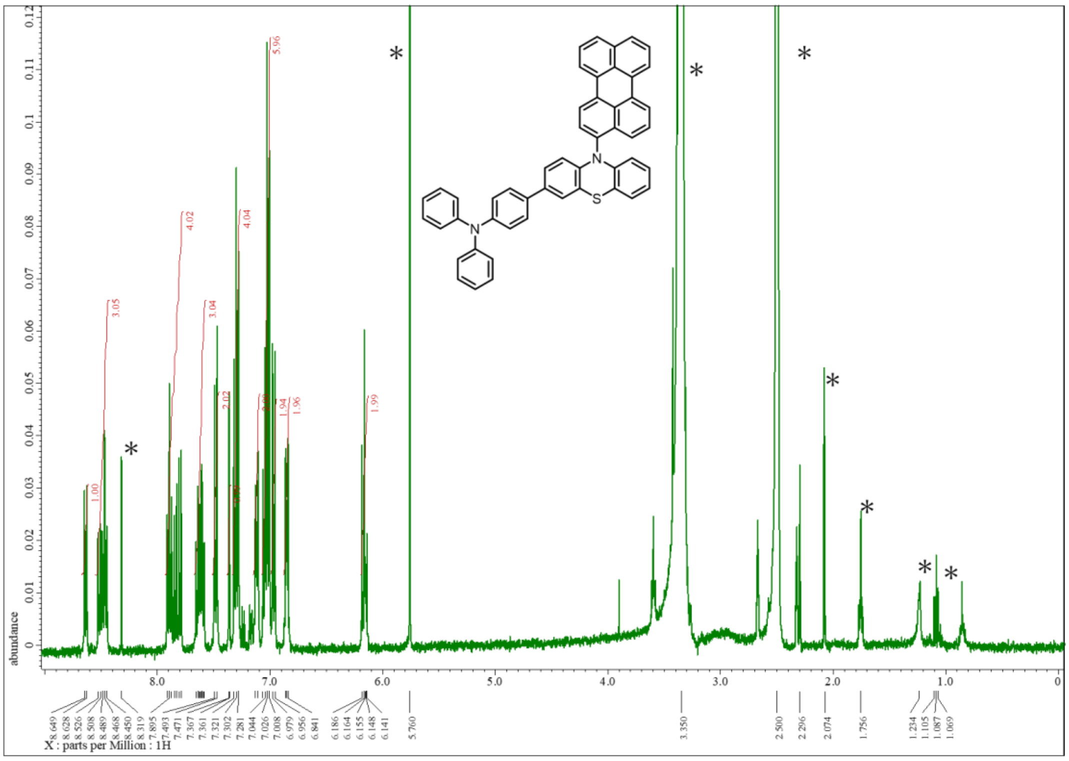

Figure S4. ^{1}H NMR spectrum of Pe–PTZ–TPA in DMSO-d_6 (* solvent peaks).

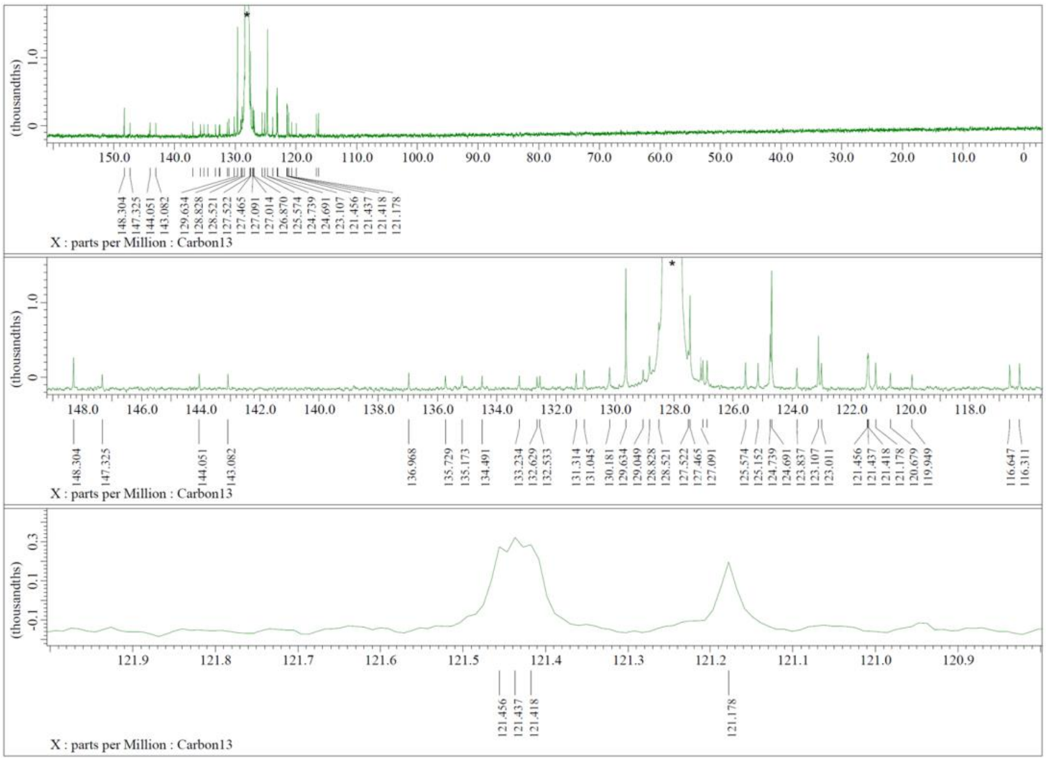

Figure S5. ^{13}C NMR spectrum of Pe–PTZ–TPA in benzene-d_6 (* solvent peaks).

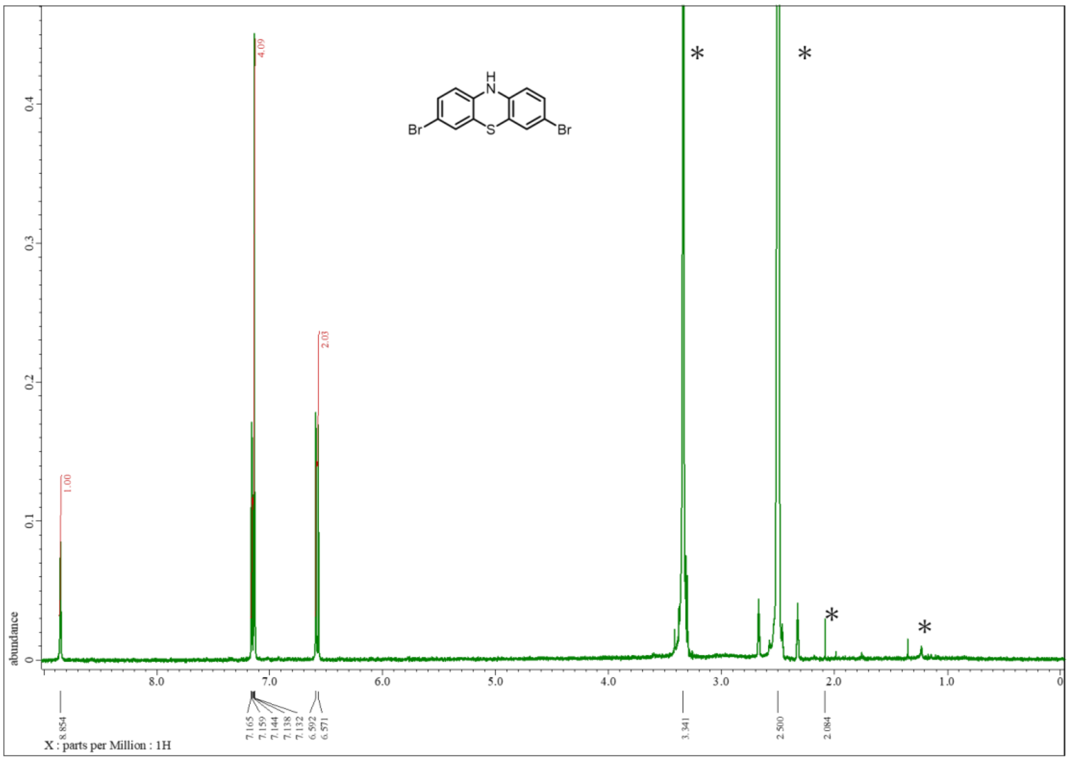

Figure S6. ^{1}H NMR spectrum of **4** in DMSO-d_6 (* solvent peaks).

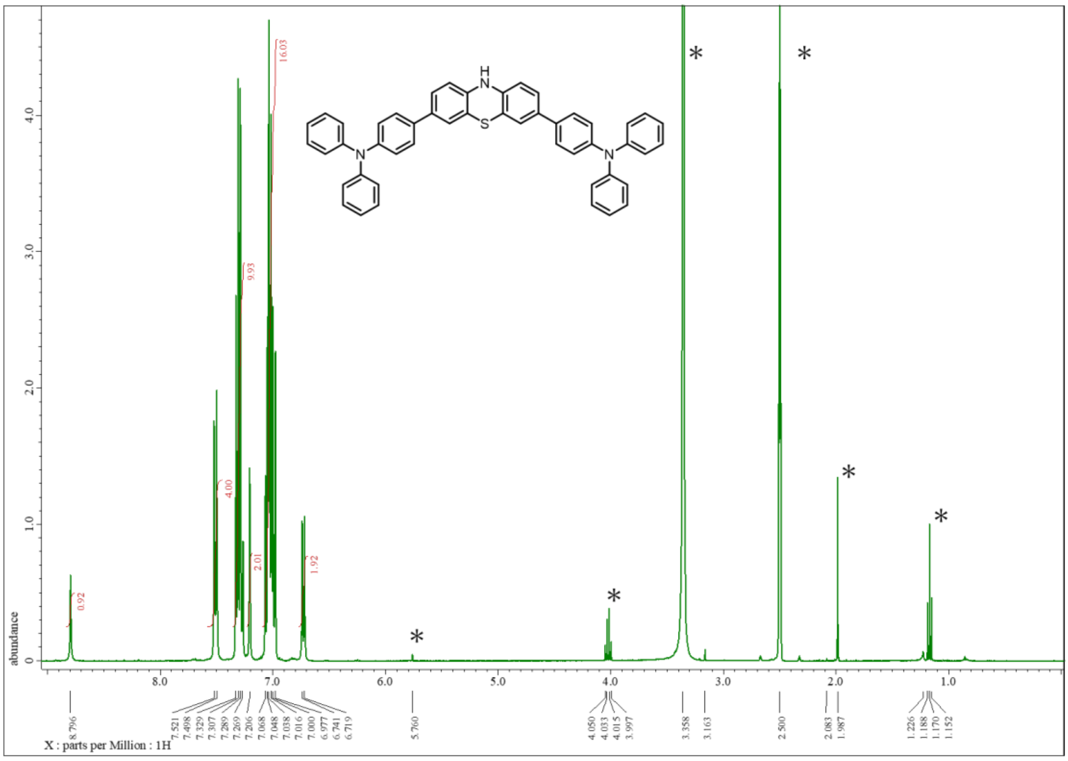

Figure S7. ^{1}H NMR spectrum of $PTZ(TPA)_2$ in DMSO-d_6 (* solvent peaks).

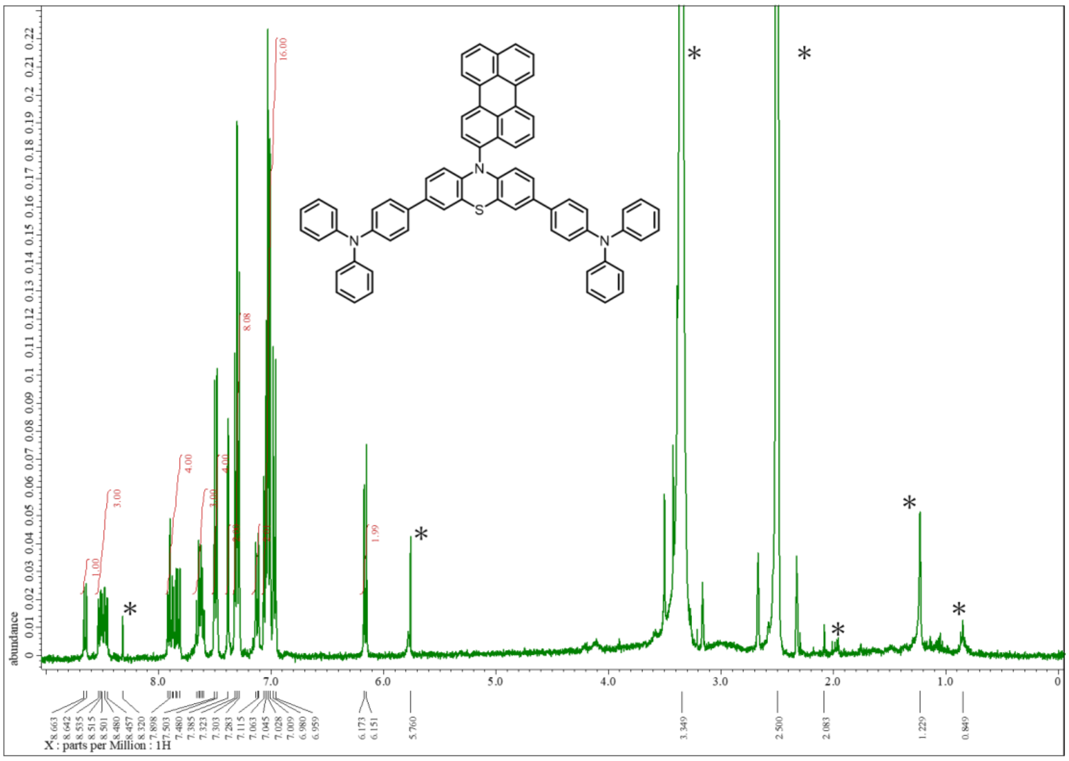

Figure S8. ^{1}H NMR spectrum of Pe–$PTZ(TPA)_2$ in DMSO-d_6 (* solvent peaks).

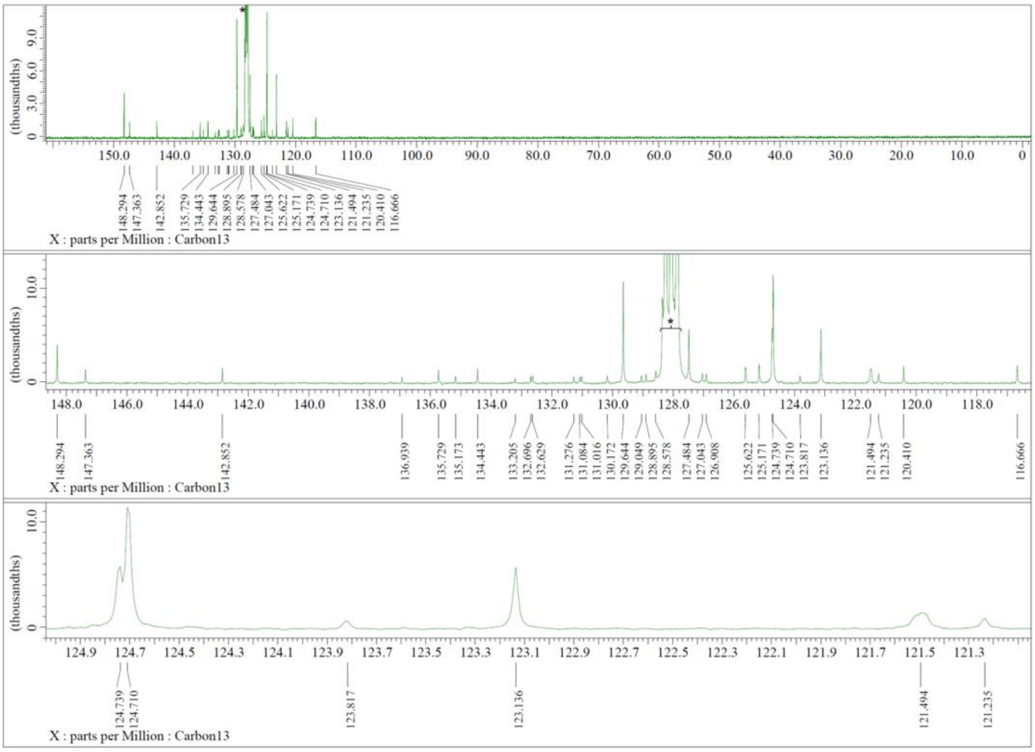

Figure S9. ^{13}C NMR spectrum of Pe–PTZ(TPA)$_2$ in benzene-*d*$_6$ (* solvent peaks).

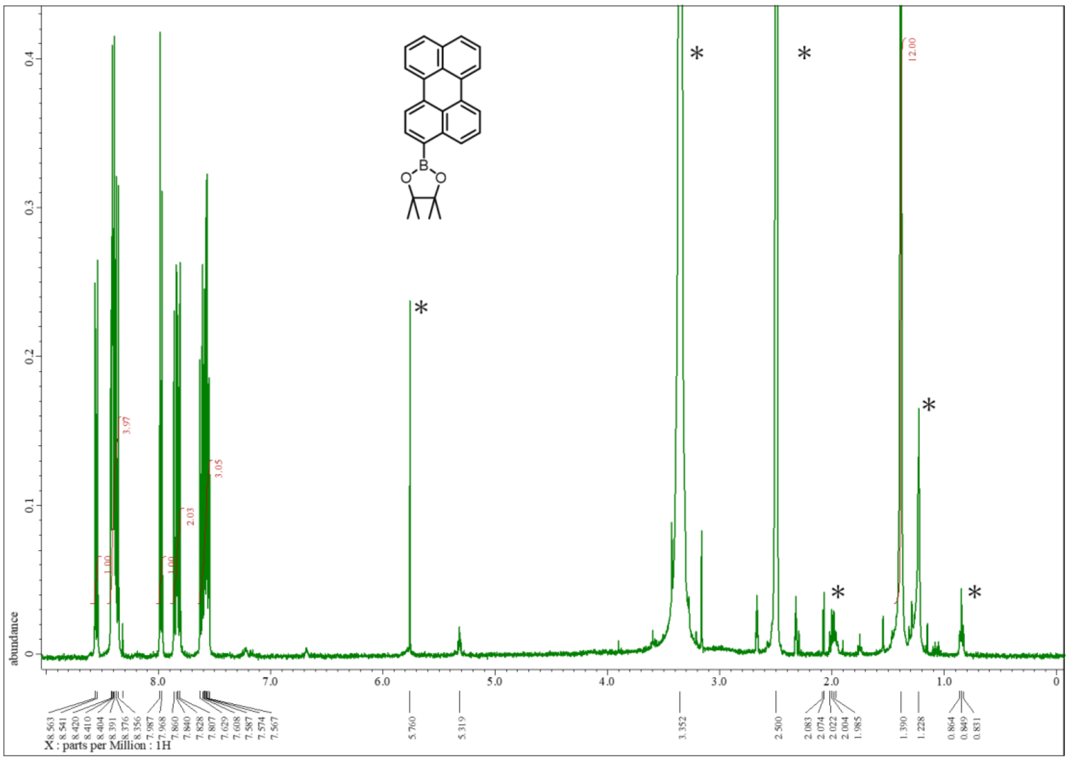

Figure S10. ^{1}H NMR spectrum of **5** in DMSO-*d*$_6$ (* solvent peaks).

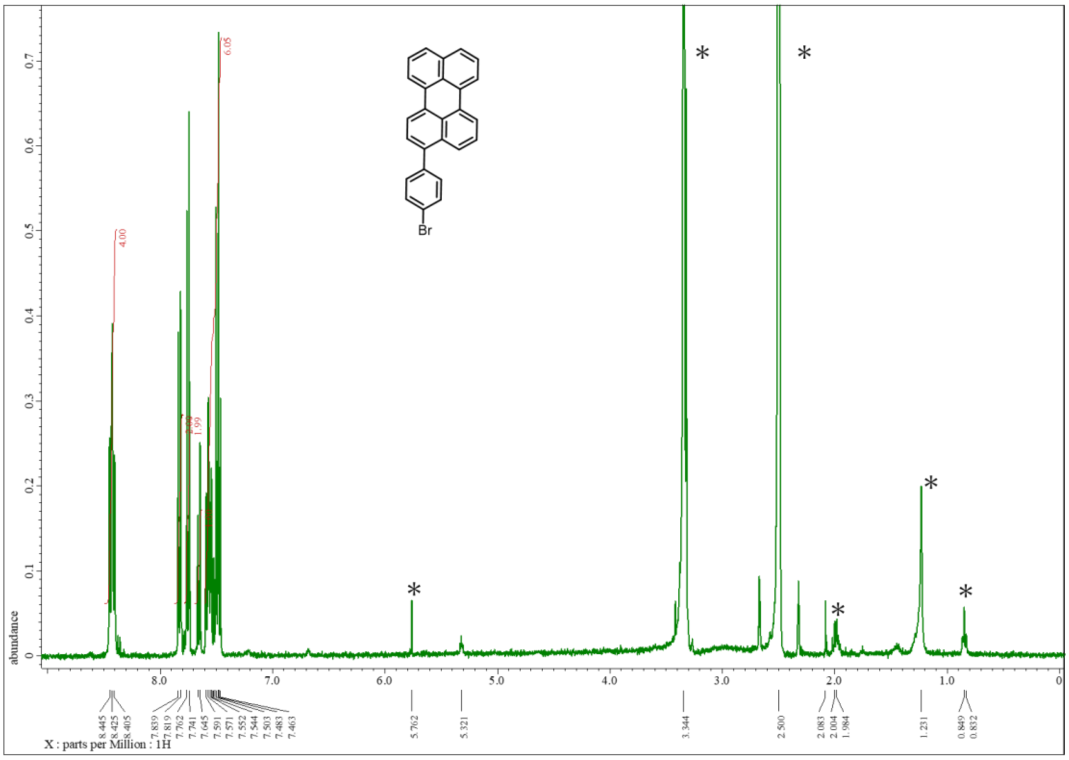

Figure S11. ^{1}H NMR spectrum of **6** in DMSO-d_6 (* solvent peaks).

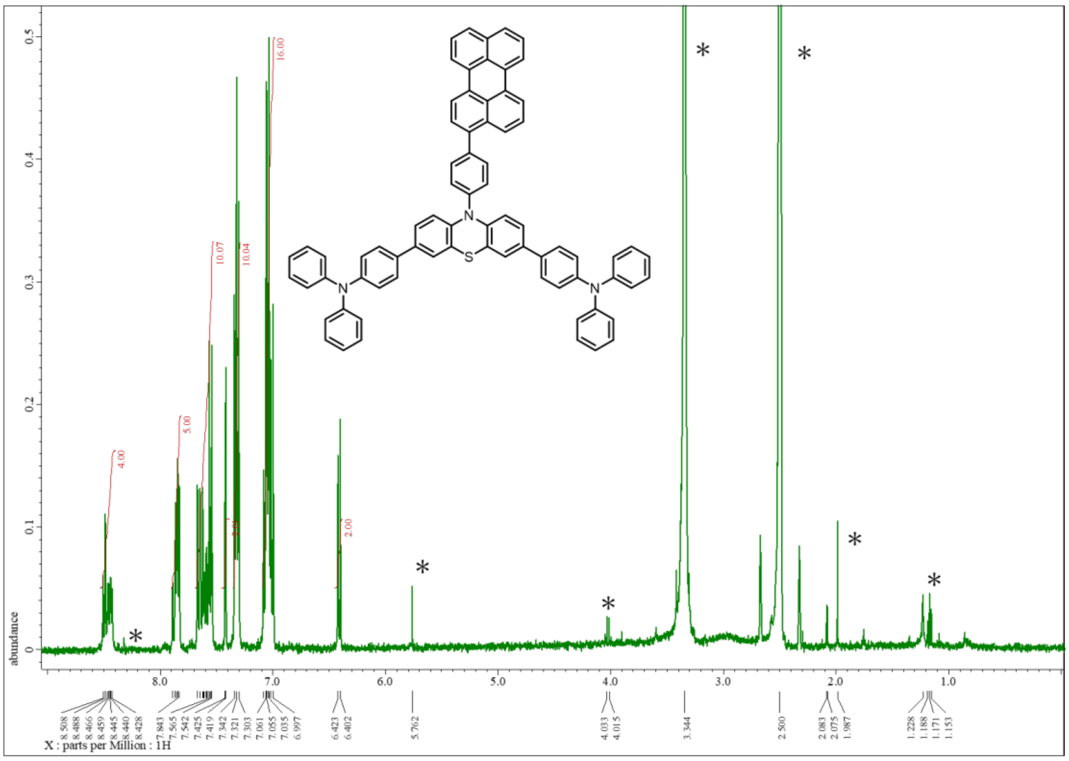

Figure S12. ^{1}H NMR spectrum of Pe–Ph–PTZ(TPA)$_2$ in DMSO-d_6 (* solvent peaks).

4. HR-ESI-TOF-MS Spectra

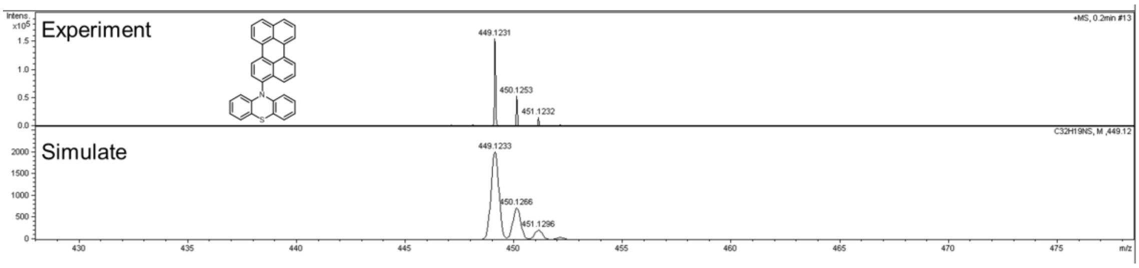

Figure S13. HR-ESI-TOF-MS of Pe–PTZ.

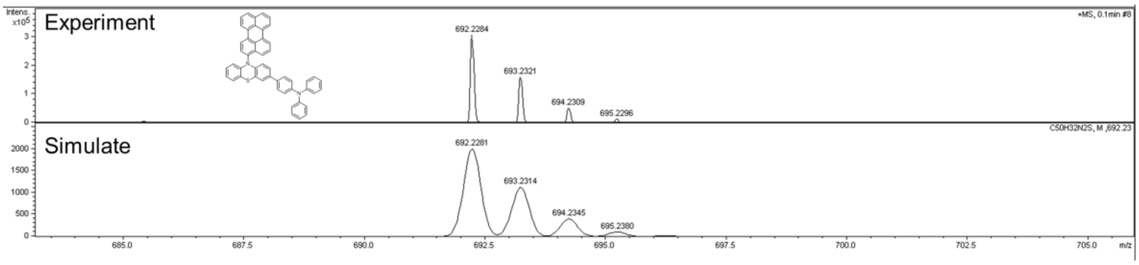

Figure S14. HR-ESI-TOF-MS of Pe–PTZ(TPA).

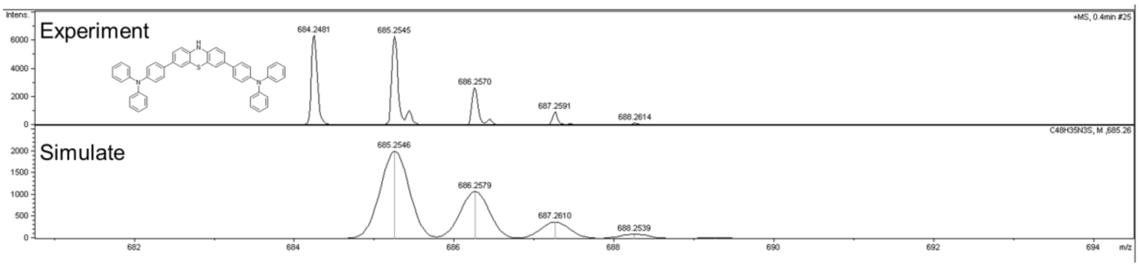

Figure S15. HR-ESI-TOF-MS of PTZ(TPA)$_2$.

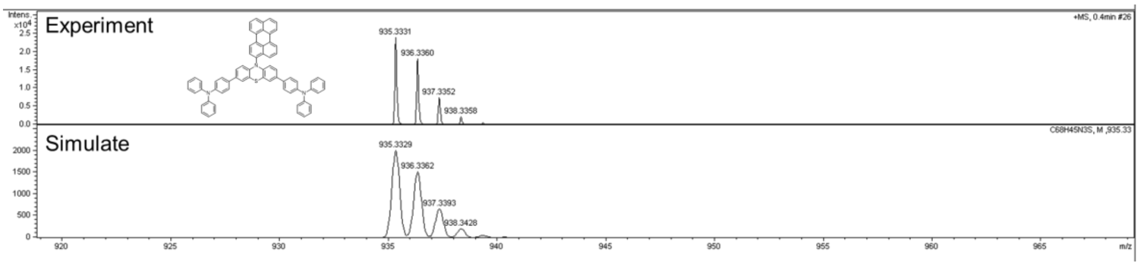

Figure S16. HR-ESI-TOF-MS of Pe–PTZ(TPA)$_2$.

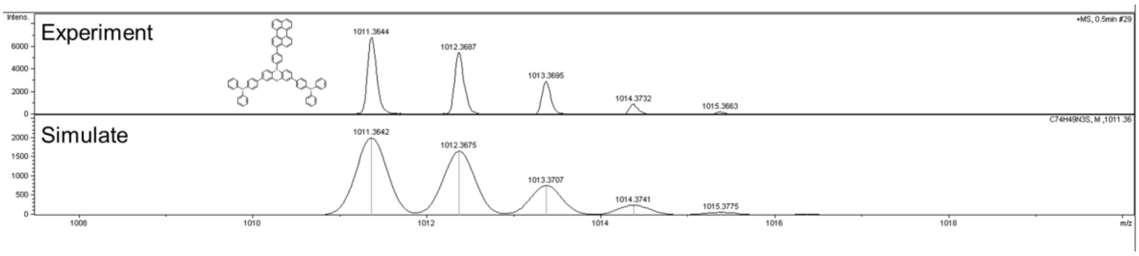

Figure S17. HR-ESI-TOF-MS of Pe−Ph−PTZ(TPA)$_2$.

5. HPLC Chromatograms

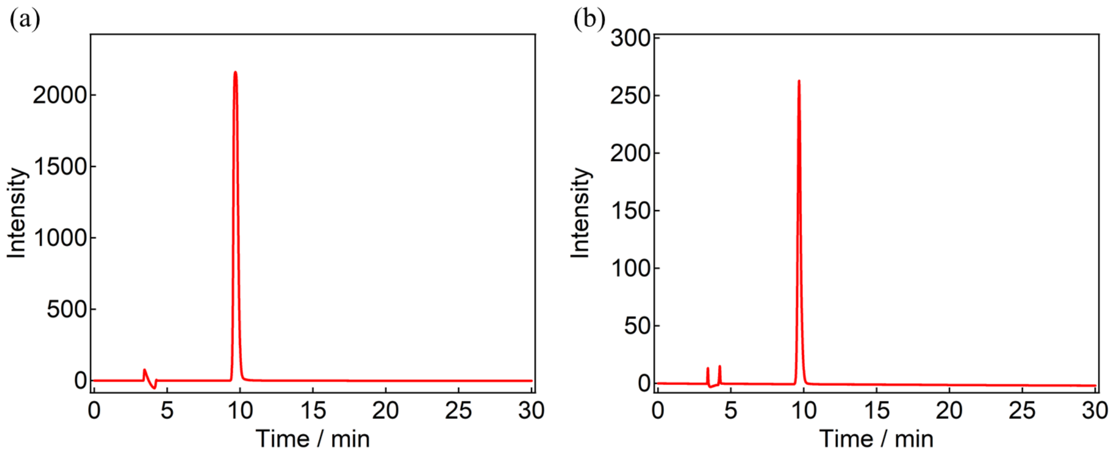

Figure S18. HPLC chromatogram of Pe–PTZ; (a) 99% and (b) 99% purity. HPLC analysis was performed using a normal phase analytical column (Mightysil RP18GP II, 25 cm × 4.6 mm, 5 µm particle) from Kanto Chemical Industries, equipped with a photodiode array (PDA) detector; the mobile phase was ethyl acetate/hexane = 1/10 with a flow rate of 1.0 mL/min (detection wavelength; (a) 254 nm (b) 365 nm). It is noted that peaks below 5 min are due to the injection solvent.

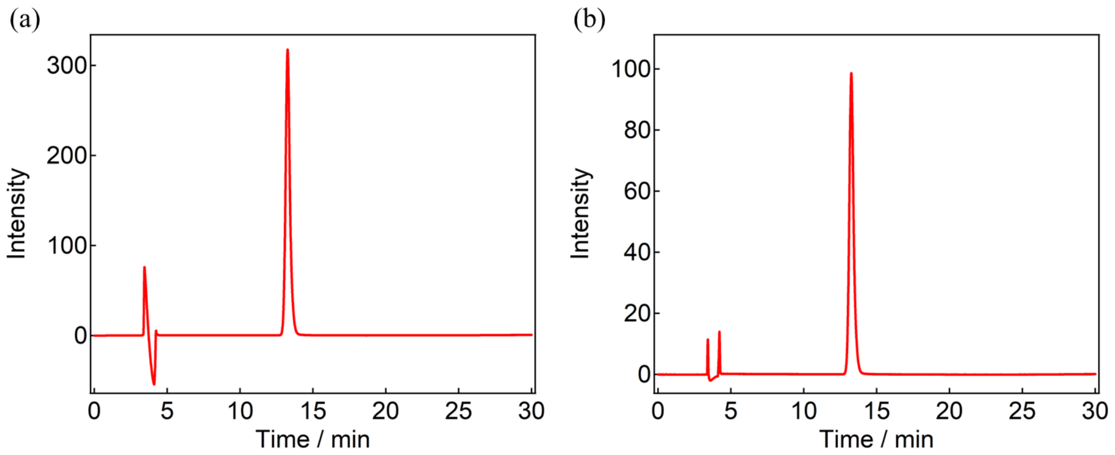

Figure S19. HPLC chromatogram of Pe–PTZ(TPA); (a) 99% and (b) 99% purity. HPLC analysis was performed using a normal phase analytical column (Mightysil RP18GP II, 25 cm × 4.6 mm, 5 µm particle) from Kanto Chemical Industries, equipped with a photodiode array (PDA) detector; the mobile phase was ethyl acetate/hexane = 1/10 with a flow rate of 1.0 mL/min (detection wavelength; (a) 254 nm (b) 365 nm). It is noted that peaks below 5 min are due to the injection solvent.

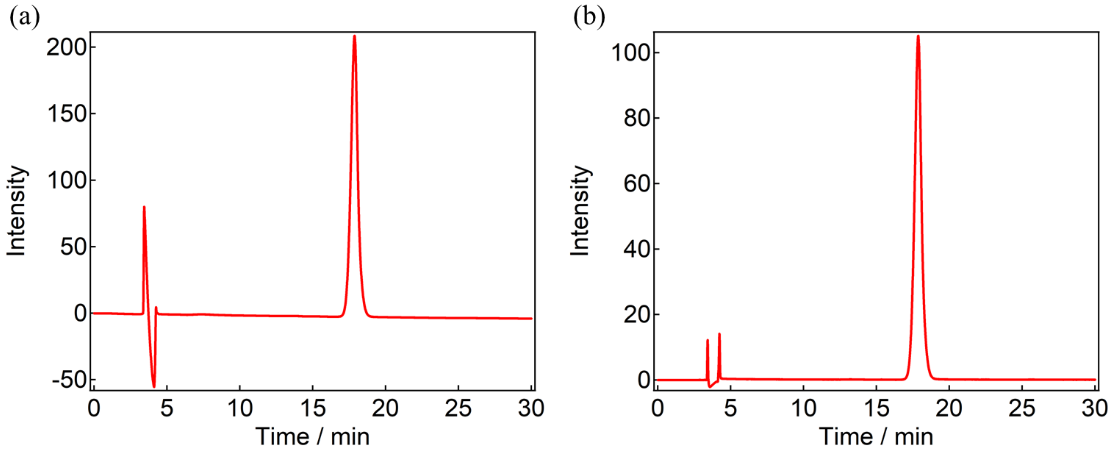

Figure S20. HPLC chromatogram of Pe–PTZ(TPA)$_2$; (a) 99% and (b) 99% purity. HPLC analysis was performed using a normal phase analytical column (Mightysil RP18GP II, 25 cm × 4.6 mm, 5 μm particle) from Kanto Chemical Industries, equipped with a photodiode array (PDA) detector; the mobile phase was ethyl acetate/hexane = 1/10 with a flow rate of 1.0 mL/min (detection wavelength; (a) 254 nm (b) 365 nm). It is noted that peaks below 5 min are due to the injection solvent.

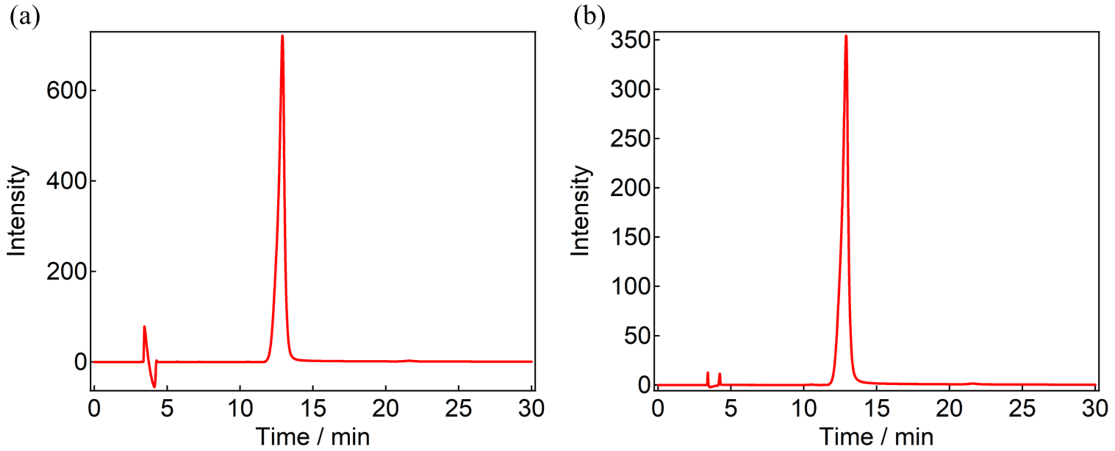

Figure S21. HPLC chromatogram of Pe–Ph–PTZ(TPA)$_2$; (a) 99% and (b) 98% purity. HPLC analysis was performed using a normal phase analytical column (Mightysil RP18GP II, 25 cm × 4.6 mm, 5 μm particle) from Kanto Chemical Industries, equipped with a photodiode array (PDA) detector; the mobile phase was ethyl acetate/hexane = 1/10 with a flow rate of 1.0 mL/min (detection wavelength; (a) 254 nm (b) 365 nm). It is noted that peaks below 5 min are due to the injection solvent.

6. Fluorescence spectra

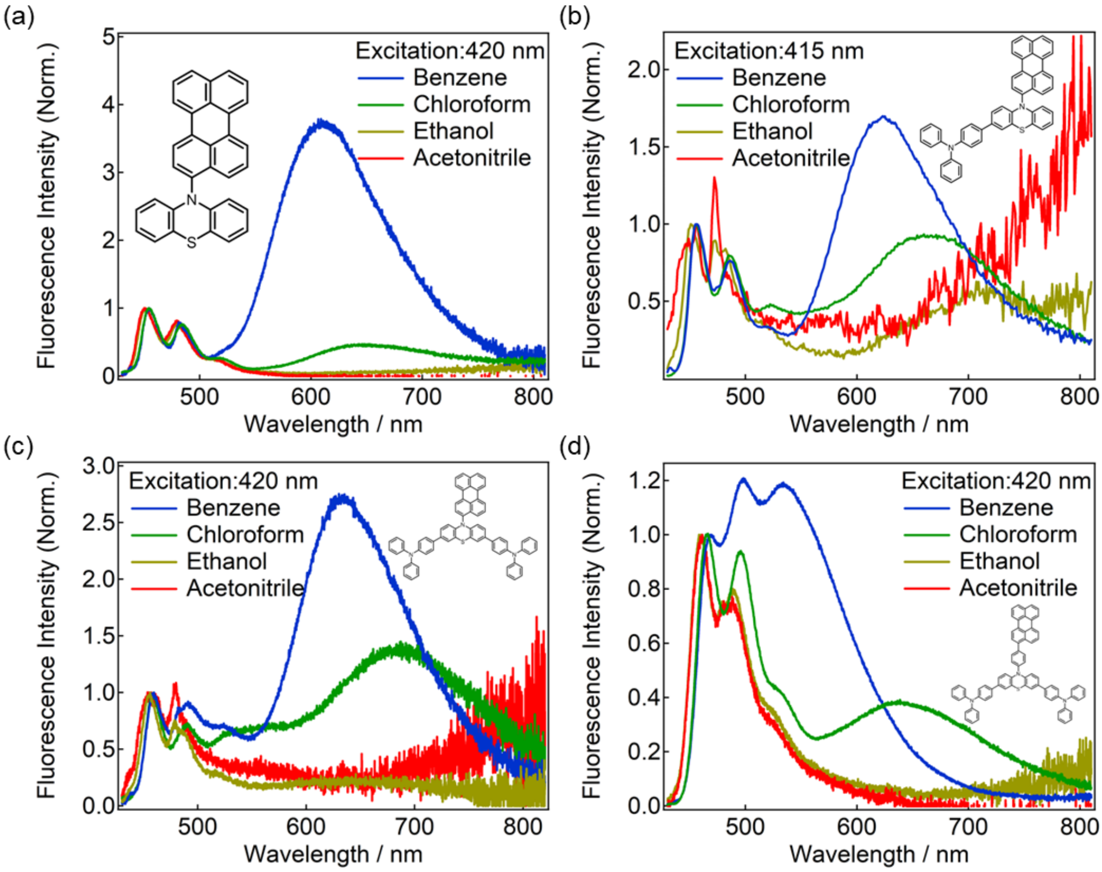

Figure S22. Fluorescence spectra excited at 415 and 420 nm of Pe–PTZ derivatives in benzene, chloroform, ethanol, and acetonitrile at room temperature.

Table S1. Relative fluorescence quantum yields of Pe–PTZ derivatives in each solution.

	benzene [%]	chloroform [%]	ethanol [%]	acetonitrile [%]
Pe–PTZ	2.4	1.6	0.4	0.3
Pe–PTZ(TPA)	3.1	1.2	0.1	0.1
Pe–PTZ(TPA)$_2$	2.4	1.0	0.2	0.1
Pe–Ph–PTZ(TPA)$_2$	20	10	2.3	0.7

7. Fluorescence lifetime measurements

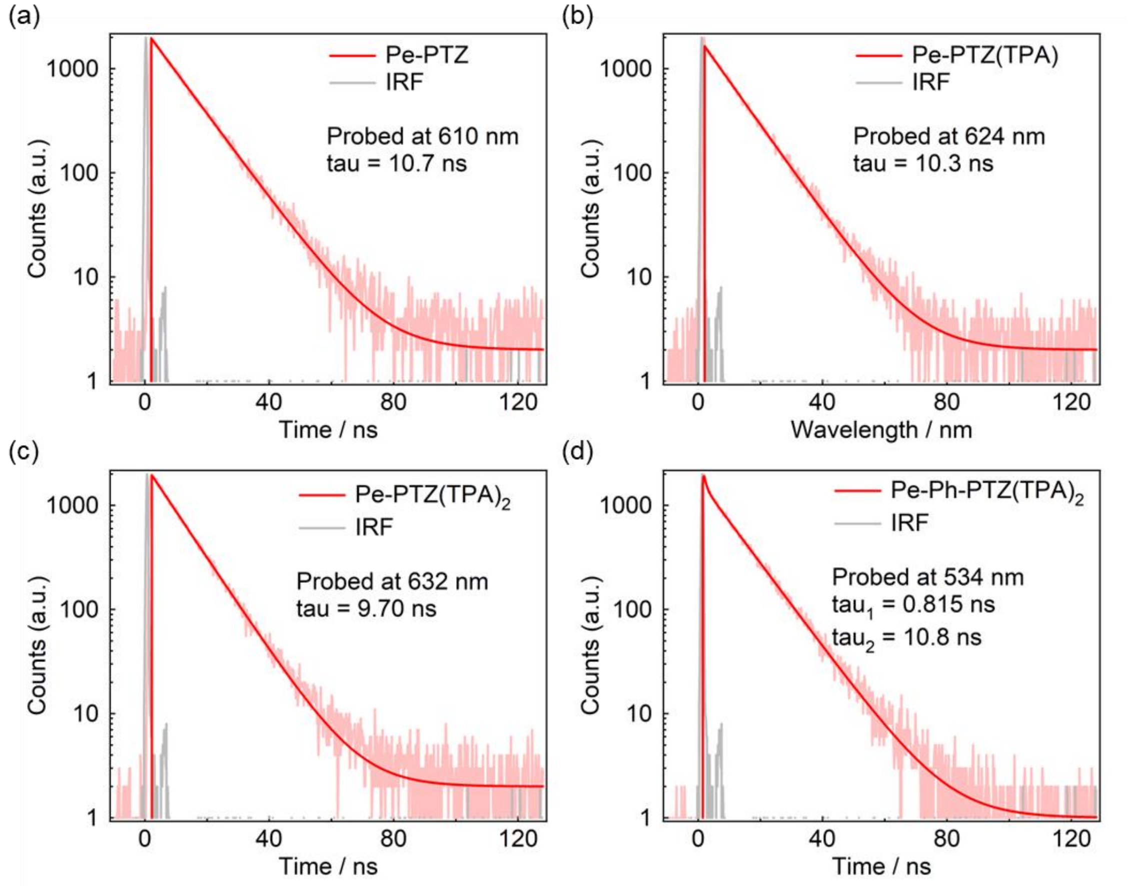

Figure S23. Fluorescence decay curves of Pe–PTZ derivatives in benzene excited at 403 nm. Bold lines indicate the fitting lines using the exponential decay function convolved with the IRF.

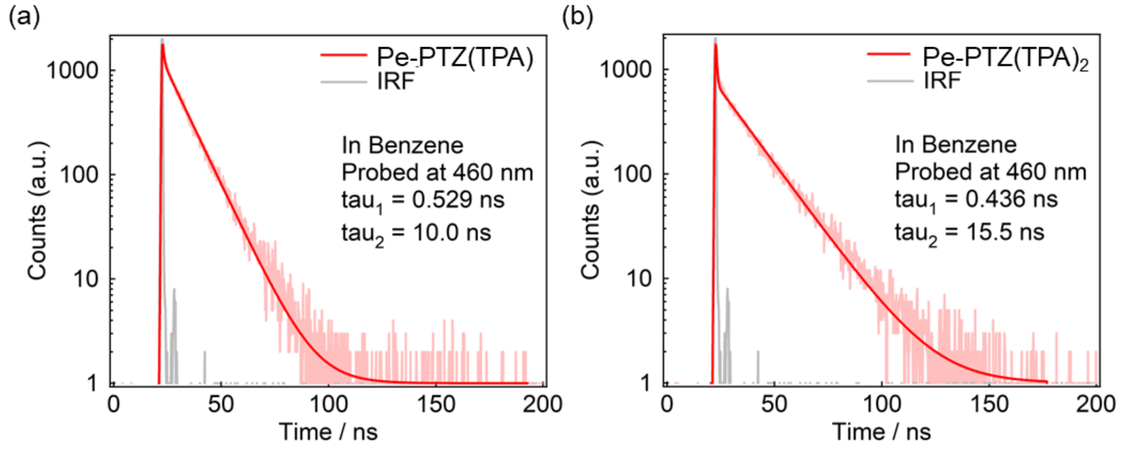

Figure S24. Fluorescence decay curves of Pe–PTZ derivatives in benzene excited at 403 nm probed at 430 nm. Bold lines indicate the fitting lines using the exponential decay function convolved with the IRF.

8. Nanosecond-to-microsecond transient absorption measurements

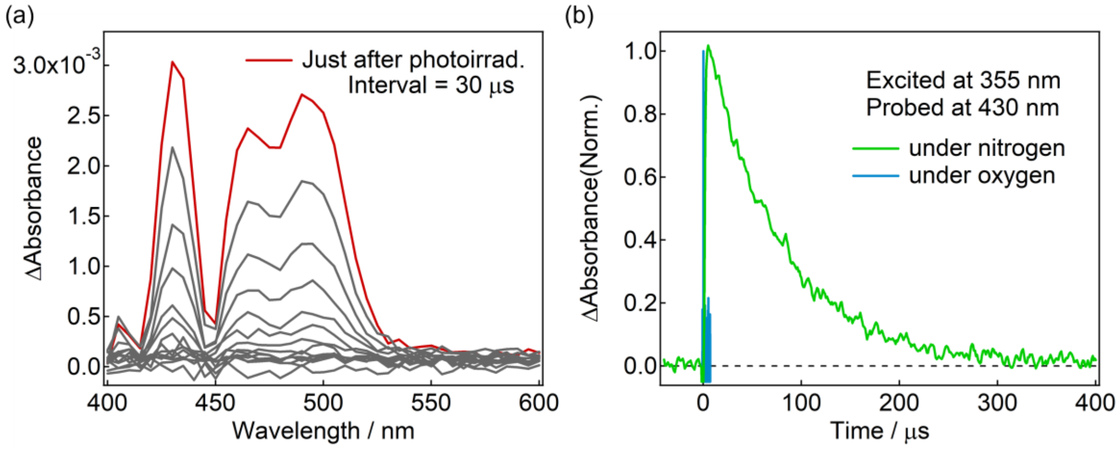

Figure S25. (a) Nanosecond-to-microsecond transient absorption spectra of Pe–PTZ in benzene and (b) nanosecond-to-microsecond transient absorption dynamics of Pe–PTZ in benzene excited with a 355-nm nanosecond laser pulse (0.5 mJ pulse^{-1}) under nitrogen and oxygen atmosphere at room temperature.

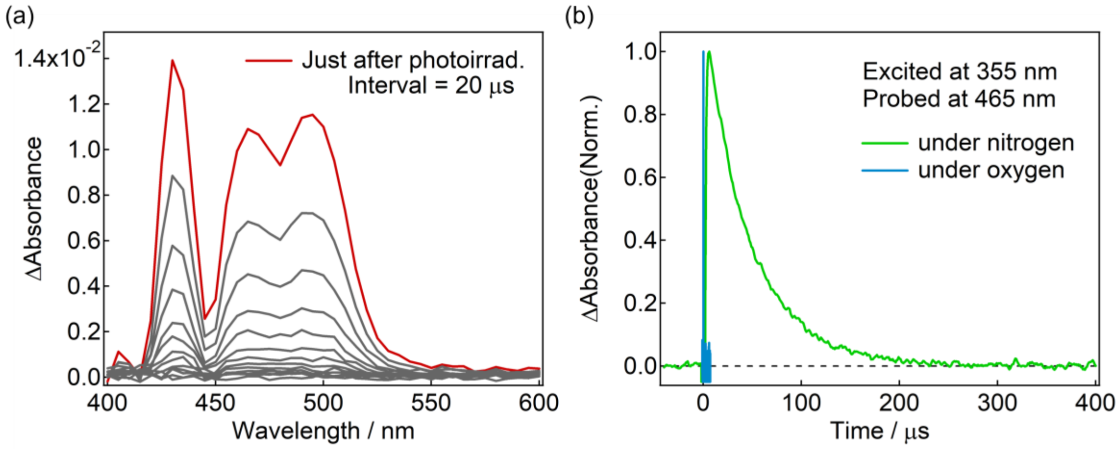

Figure S26. (a) Nanosecond-to-microsecond transient absorption spectra of Pe–PTZ(TPA) in benzene and (b) nanosecond-to-microsecond transient absorption dynamics of Pe–PTZ(TPA) in benzene excited with a 355-nm nanosecond laser pulse (0.5 mJ pulse^{-1}) under nitrogen and oxygen atmosphere at room temperature.

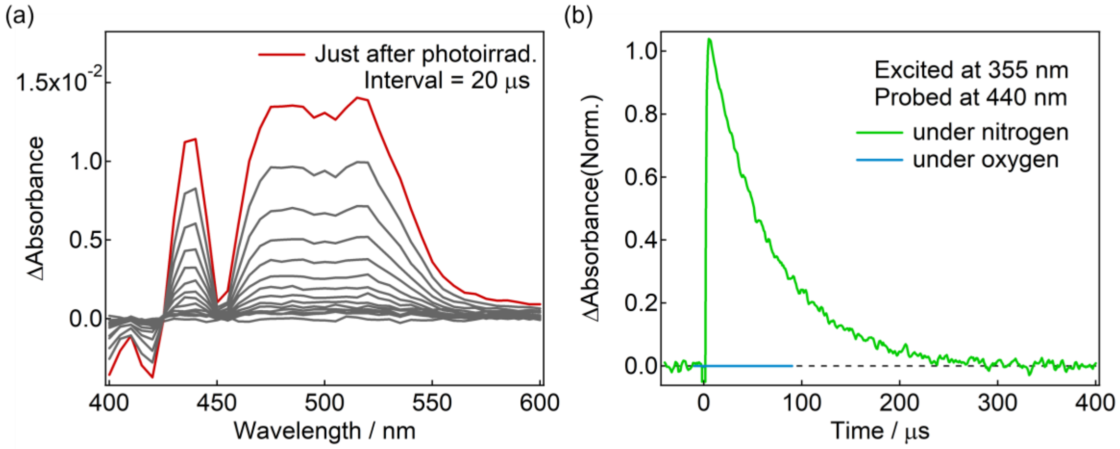

Figure S27. (a) Nanosecond-to-microsecond transient absorption spectra of Pe–Ph–PTZ(TPA)$_2$ in benzene and (b) nanosecond-to-microsecond transient absorption dynamics of Pe–Ph–PTZ(TPA)$_2$ in benzene excited with a 355-nm nanosecond laser pulse (0.5 mJ pulse^{-1}) under nitrogen and oxygen atmosphere at room temperature.

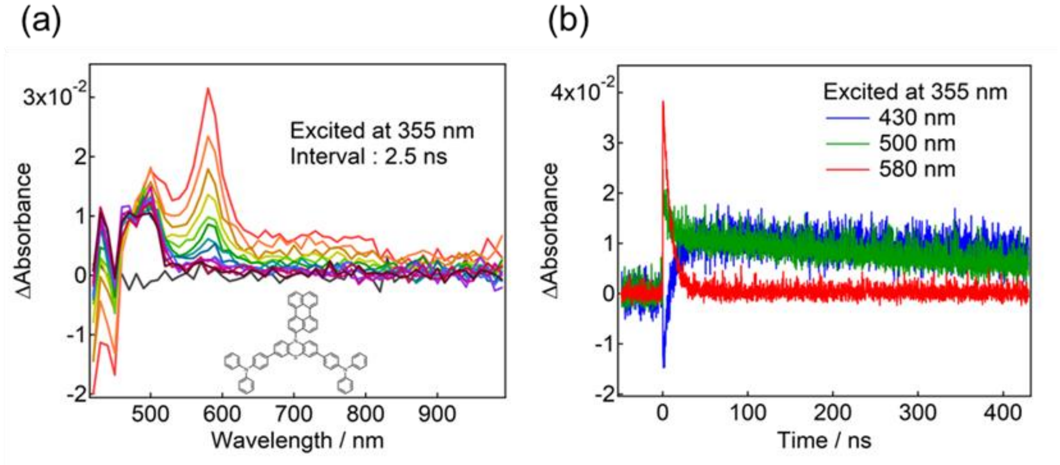

Figure S28. (a) Nanosecond-to-microsecond transient absorption spectra of Pe–PTZ(TPA)$_2$ in benzene and (b) nanosecond-to-microsecond transient absorption dynamics of Pe–PTZ(TPA)$_2$ in benzene excited with a 355-nm picosecond laser pulse (3.6 μJ pulse^{-1}) under nitrogen atmosphere at room temperature.

9. Femtosecond-to-picosecond transient absorption measurements

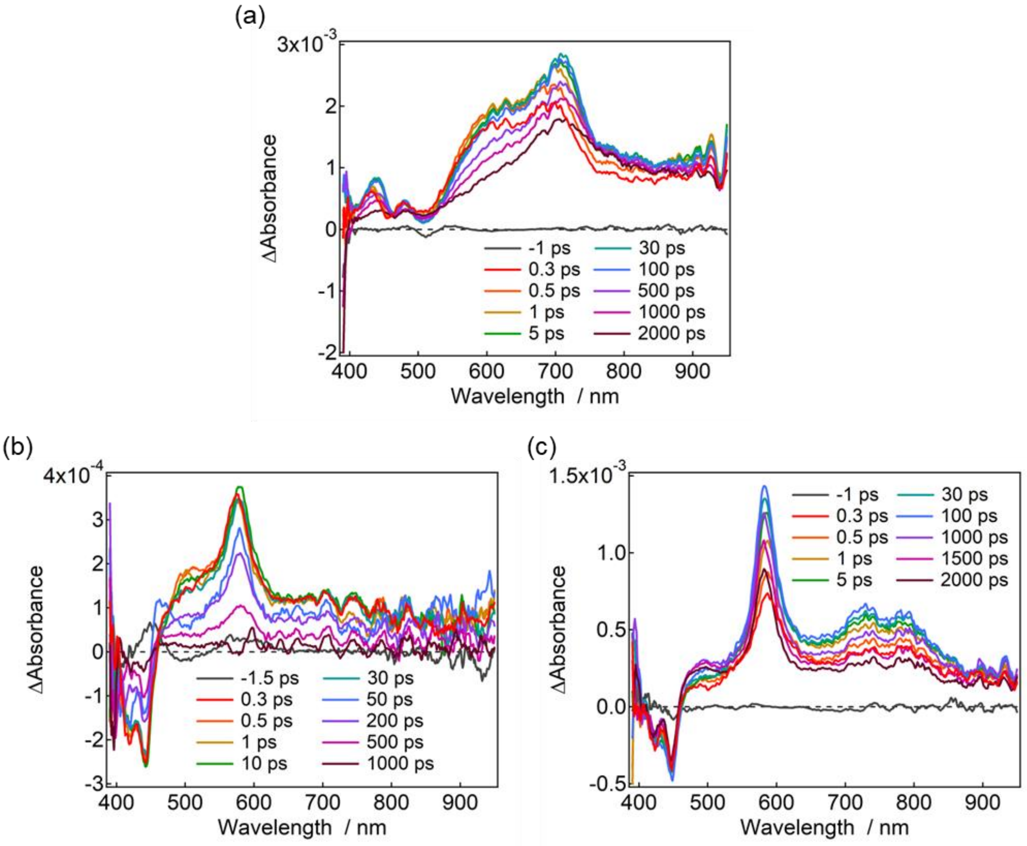

Figure S29. Femtosecond-to-nanosecond transient absorption spectra of (a) PTZ(TPA)$_2$ in benzene excited with a 390 nm femtosecond laser pulse (30 nJ pulse^{-1}), (b) Pe–PTZ(TPA)$_2$ in acetonitrile excited with a 420 nm femtosecond laser pulse (6 nJ pulse^{-1}) and (c) Pe–PTZ(TPA) in benzene excited with a 350 nm femtosecond laser pulse (20 nJ pulse^{-1}).

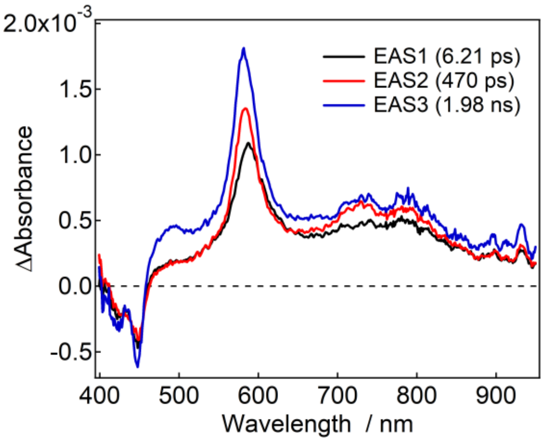

Figure S30. Evolution associated spectra (EAS) of the transient absorption spectra of Pe–PTZ(TPA) in benzene excited with a 350-nm pulse (20 nJ pulse^{-1}).

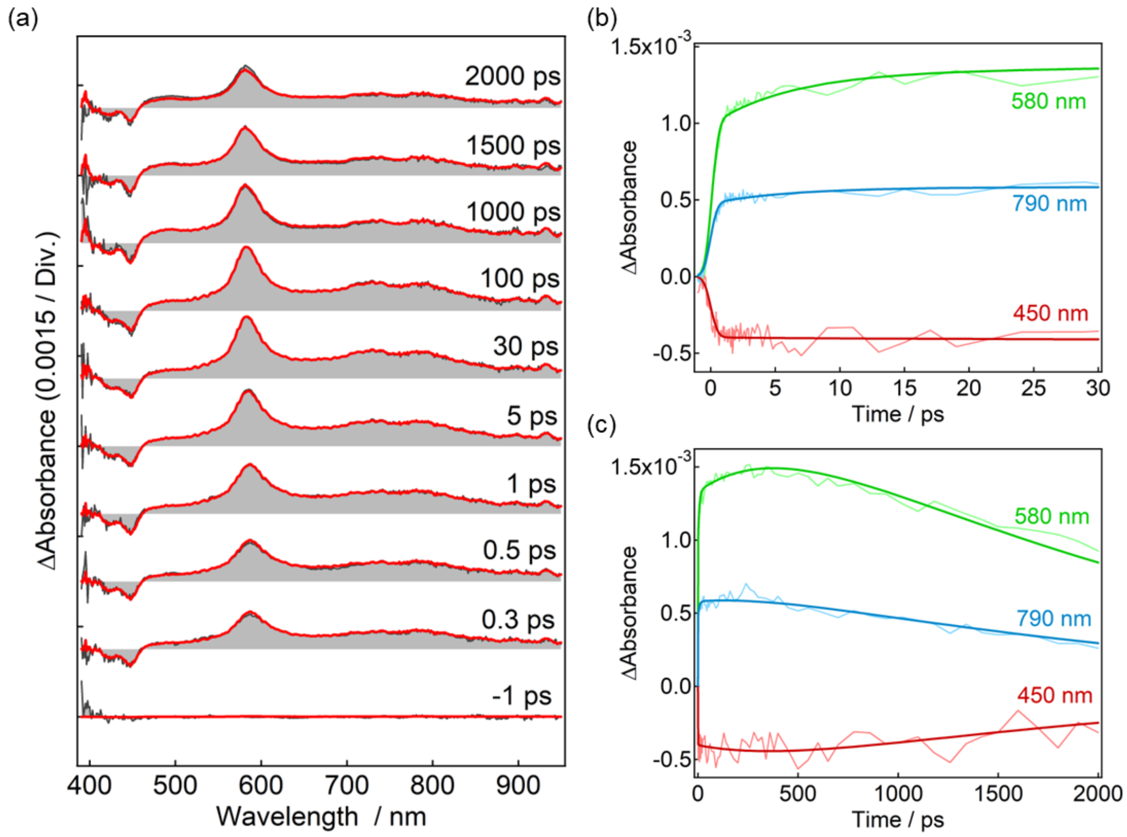

Figure S31. Time evolution of femtosecond-to-nanosecond transient absorption spectra of Pe–PTZ(TPA) in benzene excited with a 350-nm femtosecond laser pulse. Bold red, green, and blue lines show the fitting lines by SVD global analyses using a three-state sequential kinetic model.

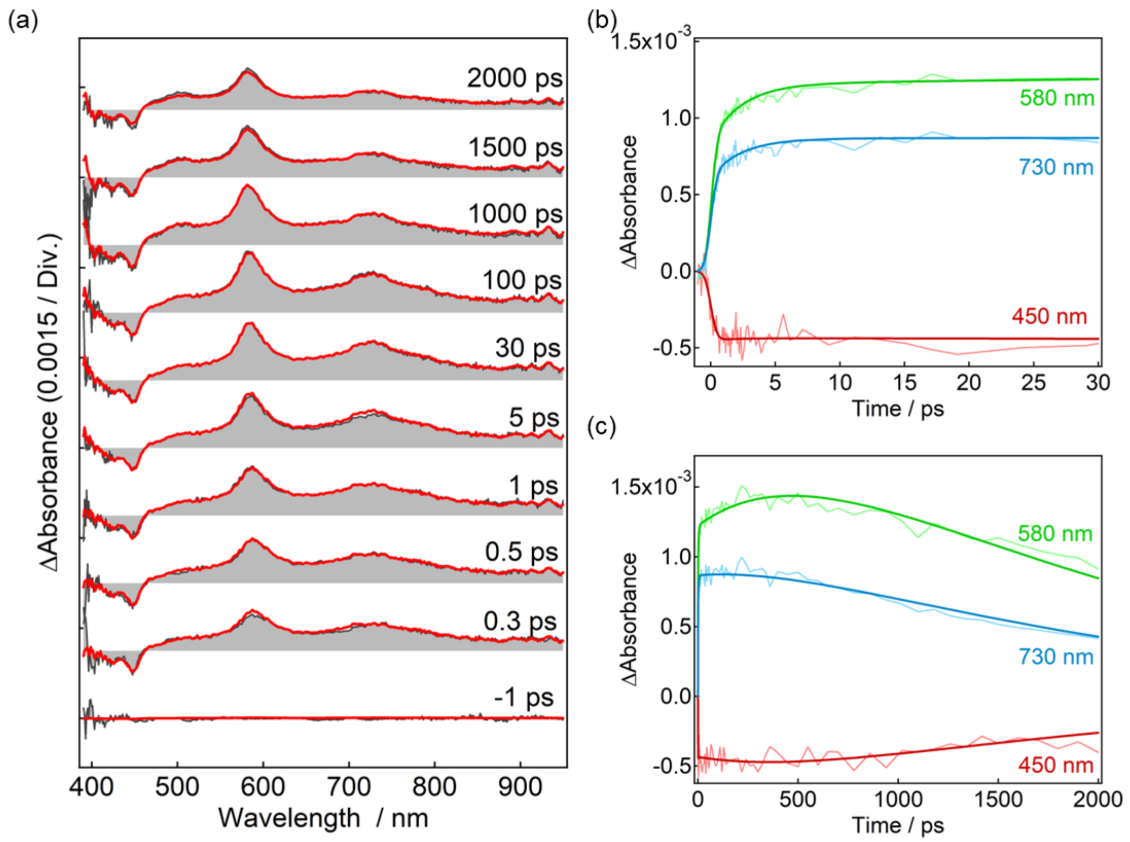

Figure S32. Time evolution of femtosecond-to-nanosecond transient absorption spectra of Pe–PTZ(TPA)$_2$ in benzene excited with a 350-nm femtosecond laser pulse. Bold red, green, and blue lines show the fitting lines by SVD global analyses using a three-state sequential kinetic model.

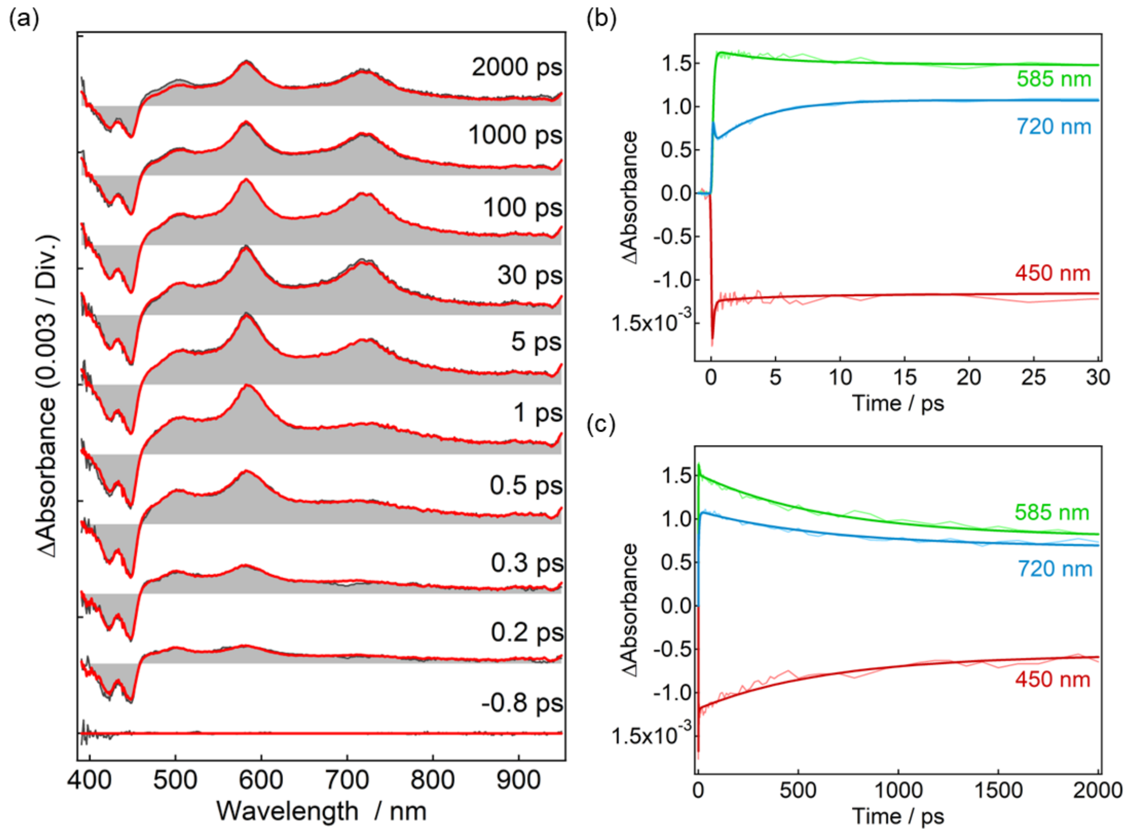

Figure S33. Time evolution of femtosecond-to-nanosecond transient absorption spectra of Pe–PTZ(TPA)$_2$ in benzene excited with a 420-nm femtosecond laser pulse. bold red, green, and blue lines show the fitting lines by SVD global analyses using a three-state sequential kinetic model.

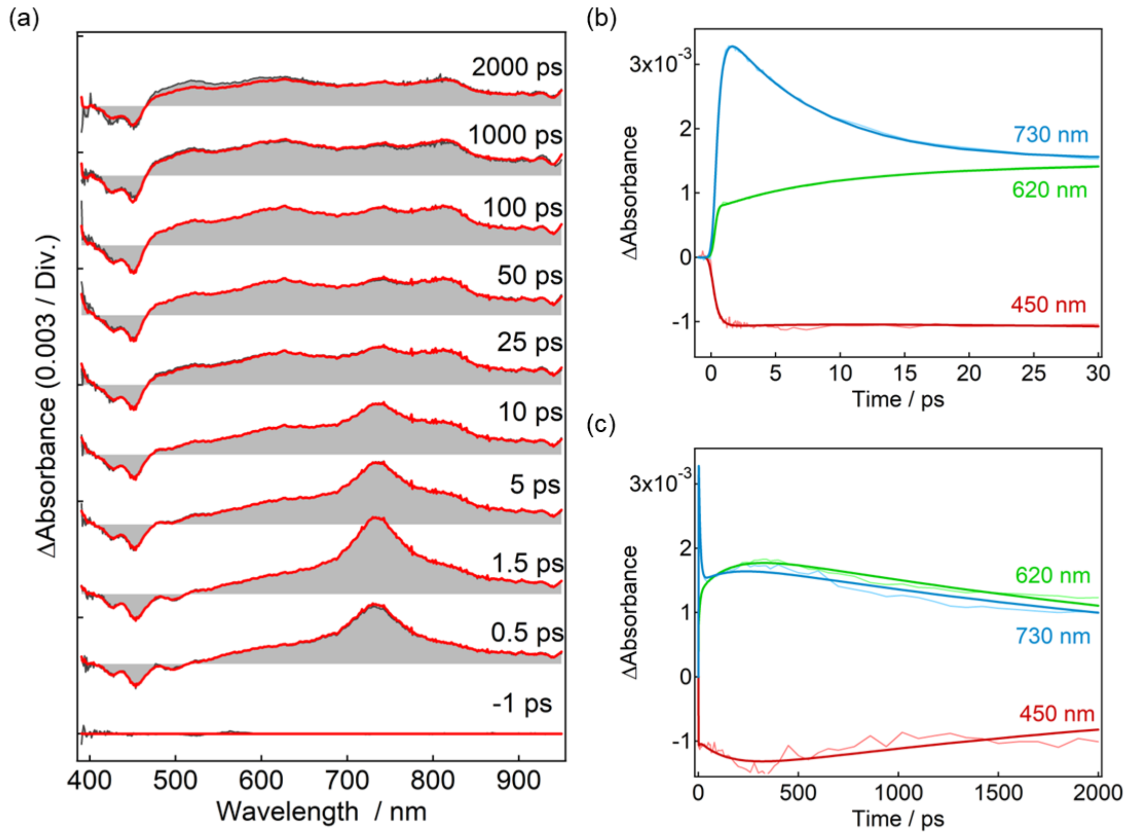

Figure S34. Time evolution of femtosecond-to-nanosecond transient absorption spectra of Pe–Ph–PTZ(TPA)$_2$ in benzene excited with a 390-nm femtosecond laser pulse. Bold red, green, and blue lines show the fitting lines by SVD global analyses using a three-state sequential kinetic model.

10. DFT calculations

All calculations were carried out using the Gaussian 16 program (Revision C.02).[9] The molecular structure was fully optimized at the B3LYP/6-31+G(d,p) level of theory, and analytical second derivative was computed using vibrational analysis to confirm each stationary point to be a minimum. TDDFT calculations were performed at the B3LYP/6-31+G(d,p) level of the theory for the optimized structures.

Table S2. Standard orientation of the optimized geometry for Pe–PTZ.

Tag	Symbol	Coordinates		
		X	Y	Z
1	C	-4.227417	-1.352005	0.211758
2	S	-4.920775	0.00959	1.126089
3	C	-4.227068	1.355582	0.189197
4	C	-2.935263	1.235217	-0.362287
5	N	-2.239008	-0.002635	-0.295798
6	C	-2.93551	-1.241295	-0.341531
7	C	-2.363753	-2.389649	-0.915053
8	C	-3.059605	-3.600206	-0.944062
9	C	-4.351975	-3.689365	-0.427717
10	C	-4.934487	-2.554619	0.140415
11	C	-4.933764	2.557054	0.097796
12	C	-4.350985	3.681918	-0.489402
13	C	-3.058741	3.583687	-1.004427
14	C	-2.363269	2.373571	-0.955181
15	C	-0.431791	0.017307	1.917382
16	C	0.065886	0.005347	0.588808
17	C	1.486275	0.003306	0.360071
18	C	2.377129	0.014159	1.484852
19	C	1.832135	0.025536	2.765595
20	C	0.442388	0.027052	2.981257
21	C	-0.820611	-0.004707	-0.530413
22	C	-0.309683	-0.016146	-1.811856
23	C	1.07753	-0.018561	-2.033108
24	C	1.990457	-0.009501	-0.982017
25	C	3.449425	-0.012782	-1.212804

26	C	4.336795	-0.000978	-0.087747
27	C	3.836917	0.013	1.255116
28	C	5.755259	-0.003233	-0.311672
29	C	6.640283	0.009052	0.797629
30	C	6.142541	0.023056	2.081599
31	C	4.752434	0.024937	2.304594
32	C	3.996216	-0.027027	-2.493623
33	C	5.387361	-0.029532	-2.709191
34	C	6.255175	-0.017713	-1.639791
35	H	-1.365234	-2.342058	-1.33032
36	H	-2.583477	-4.471506	-1.383861
37	H	-4.900902	-4.625111	-0.460947
38	H	-5.93722	-2.600287	0.555212
39	H	-5.936434	2.609995	0.511883
40	H	-4.899628	4.617147	-0.538222
41	H	-2.582436	4.447312	-1.458923
42	H	-1.36484	2.318657	-1.369777
43	H	-1.503977	0.018548	2.075729
44	H	2.479308	0.03349	3.634344
45	H	0.061589	0.036053	3.998254
46	H	-0.989723	-0.023448	-2.65847
47	H	1.421948	-0.027751	-3.060009
48	H	7.711442	0.007283	0.615736
49	H	6.817591	0.032683	2.932251
50	H	4.406205	0.036112	3.331078
51	H	3.350622	-0.0367	-3.363657
52	H	5.768534	-0.040813	-3.726004
53	H	7.330386	-0.019374	-1.796245

SCF Done: E(RB3LYP) = -1683.88936314 A.U.

Zero-point correction = 0.410970 (Hartree/Particle)
Thermal correction to Energy = 0.434972
Thermal correction to Enthalpy = 0.435916
Thermal correction to Gibbs Free Energy = 0.355855

Sum of electronic and zero-point Energies	=	-1683.49674
Sum of electronic and thermal Energies	=	-1683.472745
Sum of electronic and thermal Enthalpies	=	-1683.471801
Sum of electronic and thermal Free Energies	=	-1683.551862

Low frequencies --- -3.8712 -2.8714 -0.0018 -0.0013 0.0010 2.0945
Low frequencies --- 12.4699 24.0865 26.0989

The Result for the TDDFT calculation

Excited State 1: Singlet-A 2.2503 eV 550.97 nm f=0.0000 <S**2>=0.000
117 ->118 0.70247
This state for optimization and/or second-order correction.
Total Energy, E(TD-HF/TD-DFT) = -1683.82502019
Copying the excited state density for this state as the 1-particle RhoCI density.

Excited State 2: Singlet-A 2.7276 eV 454.55 nm f=0.4544 <S**2>=0.000
116 ->118 0.70416

Excited State 3: Singlet-A 3.3592 eV 369.09 nm f=0.0004 <S**2>=0.000
115 ->118 0.70162

Excited State 4: Singlet-A 3.5075 eV 353.48 nm f=0.0002 <S**2>=0.000
117 ->119 0.54978
117 ->120 0.35865
117 ->121 -0.24697

Excited State 5: Singlet-A 3.5739 eV 346.92 nm f=0.0003 <S**2>=0.000
114 ->118 0.33219
116 ->119 0.59423
116 ->121 0.11614

Excited State 6: Singlet-A 3.7119 eV 334.02 nm f=0.0062 <S**2>=0.000
117 ->119 -0.41581
117 ->120 0.44653
117 ->121 -0.25722

117 ->122 0.13812
117 ->124 -0.17732

Excited State 7: Singlet-A 3.8770 eV 319.79 nm f=0.0011 <S**2>=0.000
112 ->118 0.18610
113 ->118 0.19791
114 ->118 -0.33712
116 ->119 0.18623
116 ->120 0.46437
116 ->121 0.17279
116 ->124 0.12380

Excited State 8: Singlet-A 3.9373 eV 314.90 nm f=0.0103 <S**2>=0.000
109 ->118 0.20866
111 ->118 0.12123
112 ->118 0.31504
113 ->118 0.31736
114 ->118 0.10956
116 ->120 -0.21509
116 ->121 -0.28877
116 ->122 -0.12852
116 ->124 0.24996

Excited State 9: Singlet-A 3.9540 eV 313.56 nm f=0.0065 <S**2>=0.000
117 ->120 0.38876
117 ->121 0.51908
117 ->122 -0.23169

Excited State 10: Singlet-A 3.9717 eV 312.17 nm f=0.0046 <S**2>=0.000
109 ->118 -0.12034
110 ->118 -0.12553
111 ->118 0.26854
113 ->118 -0.12830
114 ->118 0.10719
116 ->120 0.14402
116 ->124 0.11067

117 ->123 0.55450

Excited State 11: Singlet-A 3.9788 eV 311.61 nm f=0.0160 <S**2>=0.000
109 ->118 0.14669
110 ->118 0.15183
111 ->118 -0.39011
113 ->118 0.16507
114 ->118 -0.14781
116 ->119 0.14093
116 ->120 -0.15902
116 ->124 -0.16284
117 ->123 0.40712

Excited State 12: Singlet-A 4.0798 eV 303.90 nm f=0.0408 <S**2>=0.000
117 ->121 0.26850
117 ->122 0.54582
117 ->125 0.29052

Excited State 13: Singlet-A 4.1143 eV 301.35 nm f=0.0005 <S**2>=0.000
110 ->118 0.41055
111 ->118 0.23823
116 ->120 -0.11831
116 ->122 0.46489
116 ->124 0.15538

Excited State 14: Singlet-A 4.1671 eV 297.53 nm f=0.0006 <S**2>=0.000
109 ->118 -0.15919
112 ->118 -0.33003
113 ->118 0.53398
114 ->118 0.22203
116 ->120 0.11122

Excited State 15: Singlet-A 4.2068 eV 294.73 nm f=0.0055 <S**2>=0.000
109 ->118 -0.22522
110 ->118 0.13404
111 ->118 -0.23025

112 ->118 0.45387
114 ->118 0.32339
116 ->119 -0.12632
116 ->120 0.11965
116 ->121 0.14071

Excited State 16: Singlet-A 4.2391 eV 292.48 nm f=0.0200 <S**2>=0.000
117 ->122 -0.33205
117 ->124 -0.41380
117 ->125 0.43251

Excited State 17: Singlet-A 4.2423 eV 292.26 nm f=0.0031 <S**2>=0.000
110 ->118 -0.13726
116 ->120 -0.31685
116 ->121 0.55937
116 ->122 -0.13248
116 ->124 0.17031

Excited State 18: Singlet-A 4.3545 eV 284.73 nm f=0.0036 <S**2>=0.000
117 ->119 -0.11231
117 ->120 0.10004
117 ->121 -0.13561
117 ->124 0.52260
117 ->125 0.41468

Excited State 19: Singlet-A 4.3695 eV 283.75 nm f=0.0000 <S**2>=0.000
116 ->123 0.70468

Excited State 20: Singlet-A 4.4096 eV 281.17 nm f=0.0014 <S**2>=0.000
110 ->118 -0.43990
111 ->118 -0.17916
116 ->122 0.46259
116 ->125 0.10693

Excited State 21: Singlet-A 4.4538 eV 278.38 nm f=0.0080 <S**2>=0.000
109 ->118 0.54299

112 ->118 -0.10075
114 ->118 0.23693
116 ->119 -0.20555
116 ->120 0.18263
116 ->121 0.12130

Excited State 22: Singlet-A 4.6002 eV 269.52 nm f=0.0345 <S**2>=0.000
111 ->118 0.16358
116 ->124 -0.39946
116 ->125 0.53181

Excited State 23: Singlet-A 4.6378 eV 267.33 nm f=0.0007 <S**2>=0.000
117 ->126 0.61513
117 ->128 -0.25931
117 ->130 0.19228

Excited State 24: Singlet-A 4.6790 eV 264.98 nm f=0.0167 <S**2>=0.000
117 ->129 0.66107
117 ->134 -0.14984
117 ->137 -0.12174

Excited State 25: Singlet-A 4.7251 eV 262.39 nm f=0.0658 <S**2>=0.000
115 ->119 0.61115
116 ->126 0.30747

Excited State 26: Singlet-A 4.7264 eV 262.32 nm f=0.0118 <S**2>=0.000
115 ->119 -0.30057
116 ->126 0.62496

Excited State 27: Singlet-A 4.7770 eV 259.54 nm f=0.1960 <S**2>=0.000
109 ->118 -0.10255
110 ->118 0.15130
110 ->119 0.10870
111 ->118 -0.19128
112 ->118 -0.12550
116 ->124 0.28752

116 ->125 0.35366
117 ->127 0.34491
117 ->128 -0.10678

Excited State 28: Singlet-A 4.8152 eV 257.48 nm f=0.1279 <S**2>=0.000
109 ->118 0.12763
110 ->119 -0.10496
111 ->118 0.13363
115 ->123 0.11017
116 ->124 -0.21368
116 ->125 -0.19417
117 ->127 0.50565
117 ->128 -0.17392

Excited State 29: Singlet-A 4.9231 eV 251.84 nm f=0.2681 <S**2>=0.000
113 ->123 -0.11794
115 ->120 0.59727
115 ->121 -0.20210
115 ->124 -0.10556
117 ->125 0.16778

Excited State 30: Singlet-A 4.9468 eV 250.63 nm f=0.0129 <S**2>=0.000
117 ->126 -0.27200
117 ->127 -0.12627
117 ->128 -0.21177
117 ->130 0.46702
117 ->131 0.32067
117 ->132 0.16180

Excited State 31: Singlet-A 4.9583 eV 250.05 nm f=0.0002 <S**2>=0.000
116 ->127 0.41774
116 ->128 0.51316
116 ->130 0.22509

Excited State 32: Singlet-A 4.9914 eV 248.40 nm f=0.0081 <S**2>=0.000
117 ->126 0.11769

117 ->127 0.18275
117 ->128 0.52435
117 ->130 0.29450
117 ->132 0.25589

Excited State 33: Singlet-A 5.0540 eV 245.32 nm f=0.0001 <S**2>=0.000
116 ->127 -0.43226
116 ->128 0.45351
116 ->130 -0.27030
116 ->131 0.16338

Excited State 34: Singlet-A 5.0757 eV 244.27 nm f=0.0406 <S**2>=0.000
115 ->120 0.29114
115 ->121 0.56498
115 ->122 -0.19284
116 ->130 -0.12497

Excited State 35: Singlet-A 5.0818 eV 243.97 nm f=0.0023 <S**2>=0.000
115 ->121 0.11814
116 ->127 -0.36026
116 ->130 0.56217
116 ->131 -0.15063

Excited State 36: Singlet-A 5.1187 eV 242.22 nm f=0.0340 <S**2>=0.000
115 ->123 0.65095
117 ->130 0.11281

Excited State 37: Singlet-A 5.1632 eV 240.13 nm f=0.0018 <S**2>=0.000
115 ->123 -0.10583
117 ->127 -0.13543
117 ->128 -0.18582
117 ->130 -0.10500
117 ->131 -0.34809
117 ->132 0.36762
117 ->133 -0.34574
117 ->135 0.16727

Excited State 38: Singlet-A 5.1973 eV 238.56 nm f=0.0089 <S**2>=0.000
117 ->128 0.10673
117 ->130 -0.33621
117 ->131 0.44168
117 ->132 0.29851
117 ->133 -0.17006
117 ->135 -0.16267

Excited State 39: Singlet-A 5.2201 eV 237.51 nm f=0.0009 <S**2>=0.000
115 ->121 0.23664
115 ->122 0.64237

Excited State 40: Singlet-A 5.2296 eV 237.08 nm f=0.0032 <S**2>=0.000
116 ->129 0.69849

Excited State 41: Singlet-A 5.2572 eV 235.84 nm f=0.0048 <S**2>=0.000
115 ->122 -0.11363
117 ->129 0.18891
117 ->134 0.59416
117 ->137 0.24855

Excited State 42: Singlet-A 5.2867 eV 234.52 nm f=0.0008 <S**2>=0.000
108 ->118 0.69884

Excited State 43: Singlet-A 5.2914 eV 234.31 nm f=0.0138 <S**2>=0.000
109 ->119 0.25936
110 ->119 0.14848
111 ->119 -0.25694
112 ->119 0.14915
113 ->119 0.29880
114 ->119 0.18404
114 ->120 0.30872
114 ->121 0.20090

Excited State 44: Singlet-A 5.3235 eV 232.90 nm f=0.0007 <S**2>=0.000

116 ->128 -0.10742
116 ->130 0.13622
116 ->131 0.57475
116 ->132 -0.28842
116 ->135 0.20543

Excited State 45: Singlet-A 5.3893 eV 230.06 nm f=0.0001 <S**2>=0.000
115 ->124 0.62768
115 ->125 -0.24219

Excited State 46: Singlet-A 5.3938 eV 229.87 nm f=0.0024 <S**2>=0.000
116 ->131 0.23003
116 ->132 0.61486
116 ->133 0.15141
116 ->135 0.14954

Excited State 47: Singlet-A 5.4094 eV 229.20 nm f=0.0741 <S**2>=0.000
109 ->119 -0.12752
111 ->119 0.11228
114 ->119 0.44205
114 ->122 -0.10930
117 ->132 -0.20592
117 ->133 -0.32329
117 ->138 0.12944

Excited State 48: Singlet-A 5.4196 eV 228.77 nm f=0.0646 <S**2>=0.000
114 ->119 0.40732
117 ->132 0.26542
117 ->133 0.39621
117 ->136 -0.10081
117 ->138 -0.15621

Excited State 49: Singlet-A 5.4611 eV 227.03 nm f=0.0046 <S**2>=0.000
109 ->119 0.12626
110 ->119 0.17273
112 ->120 -0.15620

113 ->119 -0.20433

113 ->120 -0.11712

113 ->122 0.10310

114 ->121 0.10324

117 ->131 0.17528

117 ->132 -0.11241

117 ->135 0.44826

117 ->140 -0.14325

Excited State 50: Singlet-A 5.4702 eV 226.65 nm f=0.0006 <S**2>=0.000

115 ->124 0.25398

115 ->125 0.62507

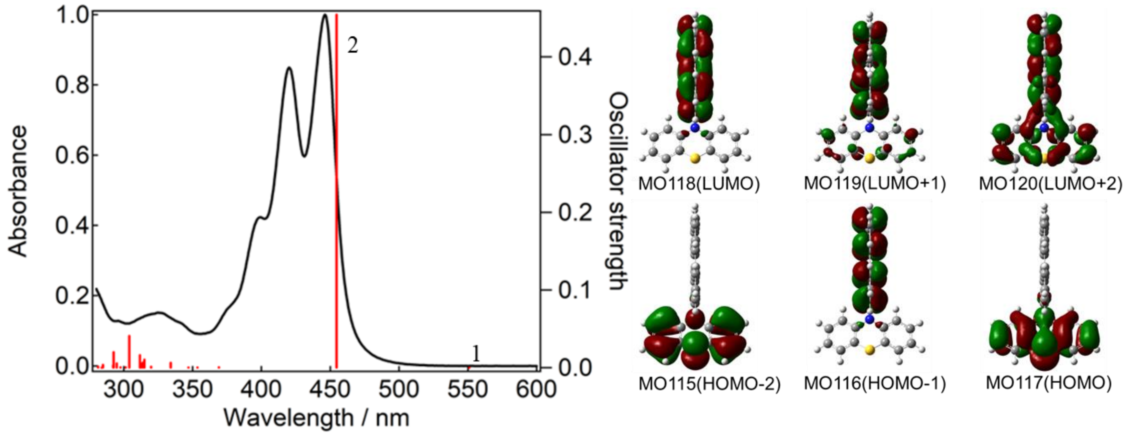

Figure S35. UV–vis absorption spectrum of Pe–PTZ in benzene at room temperature. The calculated absorption spectrum B3LYP/6-31+G(d,p)//B3LYP/6-31G(d) level of the theory) is shown by the red vertical lines. The relevant molecular orbitals of Pe–PTZ were calculated at the B3LYP/6-31+G(d,p) level of the theory.

Table S3. Standard orientation of the optimized geometry for Pe–PTZ(TPA).

Tag	Symbol	Coordinates		
		X	Y	Z
1	C	-0.594139	0.43557	0.656685
2	C	0.7359901	0.018068	0.662117
3	C	-0.9874711	1.6024051	-0.018527
4	C	1.7412531	0.7548601	0.017993
5	H	0.9946971	-0.8818181	1.2116401
6	H	-1.3320201	-0.155421	1.1840891
7	C	0.011906	2.3255672	-0.6996741
8	N	-2.3383282	2.0400301	-0.039995
9	C	1.3459321	1.9232641	-0.655187
10	C	3.1603352	0.324564	0.041498
11	S	-0.44315	3.6889143	-1.7520321
12	C	-2.6636992	3.4215492	-0.118329
13	C	-3.3638192	1.1157471	0.361542
14	H	2.0815752	2.5093892	-1.1978421
15	C	4.2080423	1.2589181	0.131806
16	C	3.5138903	-1.0354381	-0.023842
17	C	-1.8231321	4.3133763	-0.8155241
18	C	-3.8286013	3.9394683	0.472682
19	C	-3.6547373	0.9103701	1.6943231
20	C	-4.0879153	0.405012	-0.642815
21	C	5.5421114	0.8596281	0.147281
22	H	3.9777413	2.3179242	0.203236
23	C	4.8442994	-1.4456011	0.010391
24	H	2.7360182	-1.7878851	-0.114626
25	C	-2.1167672	5.6781224	-0.8671301
26	C	-4.1378893	5.2981844	0.379948
27	H	-4.5040383	3.2765822	0.9982111
28	C	-4.6574713	0.005532	2.0802632
29	H	-3.1008222	1.4556711	2.4526782
30	C	-3.8100723	0.597279	-2.0207871
31	C	-5.1161004	-0.519581	-0.245522
32	C	5.8817654	-0.502167	0.091757

33	H	6.3277844	1.6049971	0.215515
34	H	5.0847784	-2.5025872	-0.03956
35	C	-3.2807182	6.1782955	-0.279901
36	H	-1.4365551	6.3425304	-1.3920301
37	H	-5.0523874	5.6631064	0.8379651
38	C	-5.3960864	-0.7165621	1.1467541
39	H	-4.8429303	-0.115867	3.1404882
40	C	-4.5225403	-0.09871	-2.9716012
41	H	-3.0325472	1.2962001	-2.3065792
42	C	-5.8463404	-1.2314891	-1.2548901
43	N	7.2413215	-0.9152821	0.117009
44	H	-3.5108343	7.2371535	-0.342267
45	C	-6.4494935	-1.6699061	1.5507831
46	C	-5.5300794	-1.0031461	-2.5910552
47	H	-4.3094493	0.047804	-4.0263513
48	C	-6.9058025	-2.1796122	-0.8520041
49	C	8.1599836	-0.284188	1.0003411
50	C	7.6829946	-1.9597211	-0.7413391
51	C	-6.7598575	-1.9072121	2.8875962
52	C	-7.1798115	-2.3766562	0.540636
53	H	-6.0629635	-1.5268371	-3.3755272
54	C	-7.6537636	-2.8950572	-1.7838811
55	C	7.7866985	0.020123	2.3205292
56	C	9.4552157	0.046236	0.566676
57	C	7.2492895	-2.0176371	-2.0767491
58	C	8.5589556	-2.9510802	-0.267236
59	C	-7.7663416	-2.8148242	3.2683422
60	H	-6.2234875	-1.3893451	3.6737093
61	C	-8.2066656	-3.3006962	0.9329021
62	C	-8.6607606	-3.7992563	-1.3959751
63	H	-7.4718286	-2.7670752	-2.8441562
64	C	8.6880156	0.647638	3.1809452
65	H	6.7902085	-0.235338	2.6657722
66	C	10.3556737	0.658399	1.4389921
67	H	9.7507207	-0.17976	-0.452661

68	C	7.6775965	-3.0492712	-2.9126742
69	H	6.5766375	-1.2538501	-2.4530692
70	C	8.9954687	-3.9696463	-1.1148801
71	H	8.8950397	-2.9176002	0.7639721
72	C	-8.4800656	-3.5010713	2.3109922
73	H	-7.9739716	-2.9678382	4.3232343
74	C	-8.9357966	-4.0025073	-0.061964
75	H	-9.2177107	-4.3345053	-2.1593192
76	C	9.9788957	0.9675241	2.7494902
77	H	8.3814716	0.8751981	4.1980923
78	H	11.3526698	0.9066951	1.0858461
79	C	8.5558006	-4.0295673	-2.4407932
80	H	7.3317845	-3.0775412	-3.9422093
81	H	9.6725677	-4.7274663	-0.7306701
82	H	-9.2591957	-4.2030183	2.5952102
83	H	-9.7105737	-4.6987433	0.246971
84	H	10.6798868	1.4501911	3.4235882
85	H	8.8921456	-4.8274083	-3.0958472

SCF Done: E(RB3LYP) = -2432.42902531 A.U.

Zero-point correction = 0.668981 (Hartree/Particle)
Thermal correction to Energy = 0.708530
Thermal correction to Enthalpy = 0.709474
Thermal correction to Gibbs Free Energy = 0.591270
Sum of electronic and zero-point Energies = -2431.788873
Sum of electronic and thermal Energies = -2431.749324
Sum of electronic and thermal Enthalpies = -2431.748380
Sum of electronic and thermal Free Energies = -2431.866584

Low frequencies --- -1.9736 -0.0016 -0.0010 0.0020 0.5642 1.2038
Low frequencies --- 8.0809 8.3337 13.8436

The Result for the TDDFT calculation

Excited State 1: Singlet-A 2.1269 eV 582.92 nm f=0.0001 <S**2>=0.000
180 -> 182 0.23001
181 -> 182 0.66477
This state for optimization and/or second-order correction.
Total Energy, E(TD-HF/TD-DFT) = -2432.37969048
Copying the excited state density for this state as the 1-particle RhoCI density.

Excited State 2: Singlet-A 2.5654 eV 483.29 nm f=0.0019 <S**2>=0.000
180 -> 182 0.66526
181 -> 182 -0.23377

Excited State 3: Singlet-A 2.7219 eV 455.51 nm f=0.5220 <S**2>=0.000
179 -> 182 0.70152

Excited State 4: Singlet-A 3.2811 eV 377.88 nm f=0.3801 <S**2>=0.000
180 -> 183 0.17253
181 -> 183 0.65424

Excited State 5: Singlet-A 3.3459 eV 370.55 nm f=0.0001 <S**2>=0.000
178 -> 182 0.69818

Excited State 6: Singlet-A 3.4975 eV 354.49 nm f=0.1902 <S**2>=0.000
180 -> 183 -0.18189
180 -> 184 0.15471
181 -> 184 0.62462
181 -> 185 -0.11429

Excited State 7: Singlet-A 3.5665 eV 347.63 nm f=0.0095 <S**2>=0.000
180 -> 185 -0.24779
181 -> 185 0.62134
181 -> 186 -0.15457

Excited State 8: Singlet-A 3.5714 eV 347.16 nm f=0.0004 <S**2>=0.000
174 -> 182 -0.31902
179 -> 183 -0.33165
179 -> 184 0.49203

Excited State 9: Singlet-A 3.6098 eV 343.46 nm f=0.2035 <S**2>=0.000

180 -> 183 0.56374

180 -> 184 0.21648

181 -> 183 -0.20322

181 -> 184 0.15326

181 -> 186 0.11984

181 -> 191 -0.14447

Excited State 10: Singlet-A 3.7572 eV 329.99 nm f=0.0177 <S**2>=0.000

180 -> 183 -0.19928

180 -> 185 0.13282

180 -> 186 0.14627

181 -> 185 0.13872

181 -> 186 0.51304

181 -> 187 0.14279

181 -> 188 -0.15262

181 -> 189 0.21088

Excited State 11: Singlet-A 3.8192 eV 324.64 nm f=0.1499 <S**2>=0.000

180 -> 187 -0.22214

181 -> 186 -0.14438

181 -> 187 0.63306

Excited State 12: Singlet-A 3.8364 eV 323.18 nm f=0.0015 <S**2>=0.000

173 -> 182 -0.11354

174 -> 182 -0.27982

179 -> 183 0.48908

179 -> 184 0.17520

179 -> 186 0.30568

Excited State 13: Singlet-A 3.8528 eV 321.81 nm f=0.0221 <S**2>=0.000

177 -> 182 -0.22039

180 -> 186 -0.10218

180 -> 187 0.10748

180 -> 189 0.17080

181 -> 186	-0.25409
181 -> 188	-0.22481
181 -> 189	0.48032

Excited State 14: Singlet-A 3.8675 eV 320.58 nm f=0.0103 <S**2>=0.000

168 -> 182	0.12402
173 -> 182	0.14528
174 -> 182	0.14474
177 -> 182	0.57323
181 -> 186	-0.13812
181 -> 189	0.18138

Excited State 15: Singlet-A 3.9317 eV 315.34 nm f=0.0220 <S**2>=0.000

180 -> 184	0.35523
180 -> 188	0.15781
180 -> 190	0.10492
181 -> 184	-0.16186
181 -> 186	0.12434
181 -> 188	0.37356
181 -> 189	0.23857
181 -> 190	0.19842
181 -> 191	0.10748

Excited State 16: Singlet-A 3.9445 eV 314.33 nm f=0.0193 <S**2>=0.000

180 -> 183	-0.19799
180 -> 184	0.49233
180 -> 189	-0.10932
181 -> 184	-0.14755
181 -> 188	-0.23461
181 -> 189	-0.13723
181 -> 190	-0.15075
181 -> 191	-0.19028

Excited State 17: Singlet-A 3.9518 eV 313.74 nm f=0.0014 <S**2>=0.000

168 -> 182	0.15172
173 -> 182	0.17452

174 -> 182	0.27769
177 -> 182	-0.11410
179 -> 183	0.29319
179 -> 184	0.37485
179 -> 186	-0.25638
179 -> 191	-0.11264

Excited State 18: Singlet-A 3.9650 eV 312.70 nm f=0.0065 <S**2>=0.000

169 -> 182	0.16675
172 -> 182	0.36832
173 -> 182	0.29116
174 -> 182	-0.13255
177 -> 182	-0.19262
179 -> 186	0.11901
179 -> 188	0.23229
179 -> 190	0.23871
179 -> 191	-0.22120

Excited State 19: Singlet-A 3.9949 eV 310.35 nm f=0.0029 <S**2>=0.000

168 -> 182	0.34519
172 -> 182	-0.28752
173 -> 182	0.10914
174 -> 182	0.11525
177 -> 182	-0.17096
179 -> 183	-0.19871
179 -> 185	0.11012
179 -> 186	0.38252
179 -> 188	0.14019

Excited State 20: Singlet-A 4.0459 eV 306.44 nm f=0.0256 <S**2>=0.000

180 -> 185	0.16946
180 -> 190	0.15103
181 -> 188	-0.31799
181 -> 189	-0.13283
181 -> 190	0.47419
181 -> 191	0.22257

Excited State 21: Singlet-A 4.0590 eV 305.46 nm f=0.0039 <S**2>=0.000

180 -> 185 0.57612
180 -> 186 -0.12749
181 -> 185 0.21489
181 -> 186 -0.10157
181 -> 188 0.17623
181 -> 190 -0.12276
181 -> 193 0.14459

Excited State 22: Singlet-A 4.1140 eV 301.37 nm f=0.0005 <S**2>=0.000

168 -> 182 -0.21883
169 -> 182 0.34002
172 -> 182 -0.24076
179 -> 186 -0.11956
179 -> 188 0.39253
179 -> 189 0.16286
179 -> 190 -0.23308

Excited State 23: Singlet-A 4.1564 eV 298.30 nm f=0.0104 <S**2>=0.000

180 -> 185 0.14222
180 -> 186 0.30913
180 -> 188 -0.17435
180 -> 189 0.23097
180 -> 191 -0.12684
181 -> 189 -0.14881
181 -> 190 0.15044
181 -> 191 -0.24209
181 -> 192 -0.30821
181 -> 193 -0.16928

Excited State 24: Singlet-A 4.1906 eV 295.86 nm f=0.0094 <S**2>=0.000

178 -> 183 -0.15466
180 -> 184 0.13593
180 -> 186 0.34932
180 -> 192 0.14026

181 -> 186	-0.12820
181 -> 191	0.25026
181 -> 192	0.33324
181 -> 193	-0.22141

Excited State 25: Singlet-A 4.1967 eV 295.43 nm f=0.0002 <S**2>=0.000

173 -> 182	-0.16282
176 -> 182	0.66272

Excited State 26: Singlet-A 4.2093 eV 294.55 nm f=0.0053 <S**2>=0.000

173 -> 182	-0.11885
180 -> 186	-0.15333
180 -> 191	0.12223
180 -> 193	0.14407
181 -> 188	-0.10459
181 -> 190	-0.24400
181 -> 191	0.33665
181 -> 192	-0.30225
181 -> 193	-0.28143
181 -> 194	-0.10518

Excited State 27: Singlet-A 4.2129 eV 294.29 nm f=0.0017 <S**2>=0.000

169 -> 182	-0.20413
172 -> 182	-0.23200
173 -> 182	0.48458
174 -> 182	-0.22604
176 -> 182	0.22629
179 -> 188	-0.11246
181 -> 191	0.10023

Excited State 28: Singlet-A 4.2339 eV 292.84 nm f=0.0078 <S**2>=0.000

180 -> 186	0.26299
180 -> 189	0.14534
180 -> 192	-0.12983
180 -> 193	-0.12090
181 -> 188	-0.10765

181 -> 190 -0.19919
181 -> 191 0.22777
181 -> 192 -0.14291
181 -> 193 0.44288

Excited State 29: Singlet-A 4.2447 eV 292.09 nm f=0.0339 <S**2>=0.000
180 -> 186 -0.12200
180 -> 194 -0.25958
181 -> 193 -0.10885
181 -> 194 0.59920

Excited State 30: Singlet-A 4.2773 eV 289.87 nm f=0.0004 <S**2>=0.000
174 -> 182 -0.10942
175 -> 182 0.67845
177 -> 182 0.11606

Excited State 31: Singlet-A 4.2944 eV 288.71 nm f=0.0231 <S**2>=0.000
180 -> 186 -0.19931
180 -> 187 0.53865
180 -> 188 -0.11471
180 -> 189 0.23183
181 -> 187 0.15072
181 -> 189 -0.14231
181 -> 193 0.10746

Excited State 32: Singlet-A 4.3106 eV 287.62 nm f=0.0274 <S**2>=0.000
180 -> 186 -0.20756
180 -> 187 -0.34797
180 -> 188 -0.28942
180 -> 189 0.37840
181 -> 187 -0.18823
181 -> 192 0.12484

Excited State 33: Singlet-A 4.3211 eV 286.93 nm f=0.0007 <S**2>=0.000
169 -> 182 0.11425
179 -> 185 0.52264

179 -> 187 0.10301
179 -> 188 -0.17000
179 -> 189 0.37321

Excited State 34: Singlet-A 4.3823 eV 282.92 nm f=0.0002 <S**2>=0.000
179 -> 185 -0.40470
179 -> 186 0.19400
179 -> 187 0.12784
179 -> 188 -0.14029
179 -> 189 0.48621

Excited State 35: Singlet-A 4.3910 eV 282.36 nm f=0.0026 <S**2>=0.000
168 -> 182 -0.10086
169 -> 182 0.18117
179 -> 186 0.13437
179 -> 188 -0.15999
180 -> 188 0.42924
180 -> 189 0.30375
180 -> 190 0.16440
180 -> 191 0.12014
181 -> 188 -0.12907
181 -> 189 -0.11284
181 -> 191 -0.10149

Excited State 36: Singlet-A 4.4009 eV 281.73 nm f=0.0006 <S**2>=0.000
168 -> 182 -0.12159
169 -> 182 0.33686
172 -> 182 -0.11887
179 -> 186 0.15739
179 -> 188 -0.32822
179 -> 189 -0.25400
179 -> 191 -0.13222
180 -> 188 -0.23418
180 -> 189 -0.14335

Excited State 37: Singlet-A 4.4238 eV 280.27 nm f=0.0065 <S**2>=0.000

168 -> 182 0.42043
169 -> 182 0.16690
174 -> 182 -0.26307
179 -> 184 -0.19932
179 -> 186 -0.17484
180 -> 188 0.11612
180 -> 190 -0.20145
180 -> 191 -0.12492

Excited State 38: Singlet-A 4.4468 eV 278.82 nm f=0.0017 <S**2>=0.000
168 -> 182 0.22760
174 -> 182 -0.13521
180 -> 188 -0.17968
180 -> 190 0.43501
180 -> 191 0.26266
180 -> 192 0.13721
181 -> 190 -0.11745

Excited State 39: Singlet-A 4.4513 eV 278.53 nm f=0.0052 <S**2>=0.000
180 -> 196 0.13392
181 -> 195 0.47340
181 -> 196 -0.45374

Excited State 40: Singlet-A 4.5137 eV 274.68 nm f=0.0168 <S**2>=0.000
172 -> 182 -0.16276
179 -> 187 -0.18286
179 -> 190 0.52006
179 -> 191 0.29639
179 -> 192 -0.12640

Excited State 41: Singlet-A 4.5179 eV 274.43 nm f=0.0001 <S**2>=0.000
170 -> 182 0.23995
171 -> 182 0.64757

Excited State 42: Singlet-A 4.5338 eV 273.47 nm f=0.0013 <S**2>=0.000
179 -> 187 0.65411

```
      179 -> 188         0.13933
      179 -> 189        -0.14191
      179 -> 190         0.12588

 Excited State  43:      Singlet-A      4.5427 eV  272.93 nm  f=0.0060  <S**2>=0.000
      180 -> 195         0.20025
      180 -> 196        -0.12493
      181 -> 195         0.36758
      181 -> 196         0.43152
      181 -> 198        -0.19353
      181 -> 199         0.15694

 Excited State  44:      Singlet-A      4.5520 eV  272.37 nm  f=0.0819  <S**2>=0.000
      178 -> 183         0.34274
      180 -> 190         0.31938
      180 -> 191        -0.23242
      180 -> 192        -0.25121
      181 -> 190        -0.11760
      181 -> 192         0.23905
      181 -> 195         0.10892

 Excited State  45:      Singlet-A      4.5619 eV  271.78 nm  f=0.0000  <S**2>=0.000
      170 -> 182         0.64941
      171 -> 182        -0.25866

 Excited State  46:      Singlet-A      4.5971 eV  269.70 nm  f=0.2054  <S**2>=0.000
      178 -> 183         0.43084
      180 -> 190        -0.23387
      180 -> 191         0.39865
      181 -> 192         0.13149
      181 -> 197        -0.17576

 Excited State  47:      Singlet-A      4.6402 eV  267.20 nm  f=0.0319  <S**2>=0.000
      178 -> 183        -0.18551
      180 -> 191         0.21092
      180 -> 192        -0.25163
```

180 -> 193 -0.28919
180 -> 196 -0.15860
180 -> 200 0.10642
181 -> 193 -0.13246
181 -> 199 -0.17481
181 -> 200 0.34017

Excited State 48: Singlet-A 4.6476 eV 266.77 nm f=0.0525 <S**2>=0.000
180 -> 191 -0.20378
180 -> 192 0.41844
180 -> 196 -0.10615
181 -> 191 0.11183
181 -> 197 -0.29979
181 -> 200 0.23885

Excited State 49: Singlet-A 4.6653 eV 265.76 nm f=0.0155 <S**2>=0.000
178 -> 183 -0.10314
180 -> 191 0.14345
180 -> 193 0.53481
180 -> 194 0.10136
181 -> 193 0.15497
181 -> 200 0.23684

Excited State 50: Singlet-A 4.6950 eV 264.08 nm f=0.0345 <S**2>=0.000
172 -> 182 0.11784
179 -> 191 0.37169
179 -> 192 0.53588
179 -> 193 -0.11681

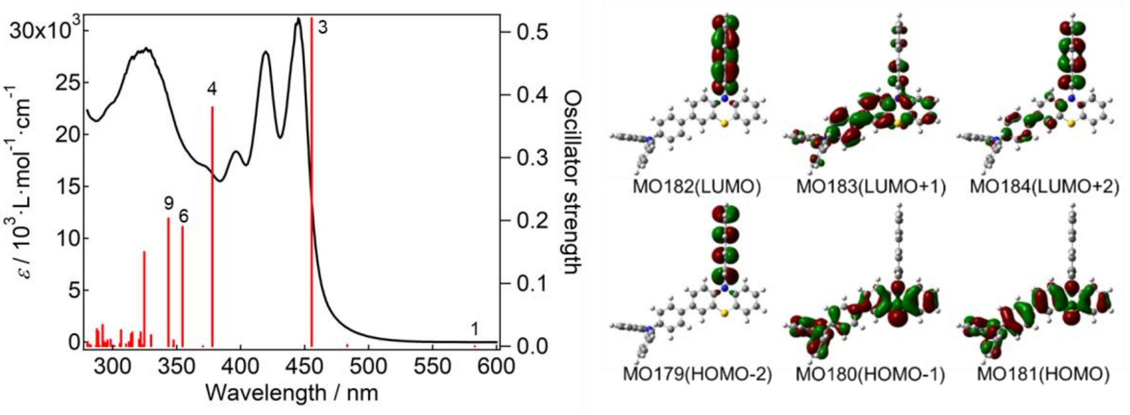

Figure S36. UV–vis absorption spectrum of Pe–PTZ(TPA) in benzene at room temperature. The calculated absorption spectrum B3LYP/6-31+G(d,p)//B3LYP/6-31G(d) level of the theory) is shown by the red vertical lines. The relevant molecular orbitals of Pe–PTZ(TPA) calculated at the B3LYP/6-31+G(d,p) level of the theory.

Table S4. Standard orientation of the optimized geometry for Pe–PTZ$(TPA)_2$.

Tag	Symbol	Coordinates		
1	C	3.5974257	0.7313017	-0.5916095
2	C	3.7078521	-0.5652046	-0.0662565
3	C	2.5529231	-1.1218200	0.5091590
4	C	1.3545029	-0.4119320	0.5783429
5	C	1.2393252	0.8728485	0.0117321
6	C	2.3910171	1.4292139	-0.5684942
7	S	-0.0039305	-1.1061819	1.4970259
8	C	-1.3559079	-0.4067983	0.5715427
9	C	-1.2355331	0.8825837	0.0154748
10	N	0.0041886	1.5734512	0.0495793
11	C	-2.5605690	-1.1056089	0.5092889
12	C	-3.7150289	-0.5369659	-0.0554936
13	C	-3.5985527	0.7620995	-0.5729754
14	C	-2.3876467	1.4522554	-0.5508102
15	C	0.0093410	2.9843904	-0.2272967
16	C	0.0151728	3.9051900	0.8635314
17	C	0.0206641	5.3176270	0.5898144
18	C	0.0207187	5.7789821	-0.7676783
19	C	0.0147622	4.8333033	-1.7895203
20	C	0.0091310	3.4538878	-1.5243899
21	C	0.0157740	3.4497487	2.2072358
22	C	0.0213672	4.3571504	3.2428979
23	C	0.0263732	5.7393926	2.9833048
24	C	0.0259633	6.2436169	1.6859201
25	C	0.0307033	7.6953851	1.4100300
26	C	0.0314389	8.1524082	0.0519354
27	C	0.0270501	7.2298863	-1.0445029
28	C	0.0367412	9.5630419	-0.2168669
29	C	0.0380338	10.0205738	-1.5602502
30	C	0.0342307	9.1193383	-2.6016952
31	C	0.0287890	7.7357518	-2.3420957
32	C	0.0345050	8.6436942	2.4300307
33	C	0.0394756	10.0260196	2.1630638

34	C	0.0407120	10.4827761	0.8638726
35	N	-8.6837063	-3.4205251	-0.2324664
36	N	8.6578112	-3.4810028	-0.2523995
37	C	-8.7919138	-4.6917253	0.3963611
38	C	-9.8046023	-2.8633077	-0.9074824
39	C	9.8341850	-2.8548624	-0.7492115
40	C	8.7053691	-4.8306787	0.1940825
41	C	7.6751925	-5.7280162	-0.1347853
42	C	7.7217541	-7.0487078	0.3125703
43	C	8.7994684	-7.5029341	1.0788054
44	C	9.8299194	-6.6142953	1.4004215
45	C	9.7832259	-5.2875570	0.9713517
46	C	10.1417714	-1.5302619	-0.3947325
47	C	11.2924616	-0.9170850	-0.8911238
48	C	12.1637226	-1.6145492	-1.7333911
49	C	11.8643555	-2.9353320	-2.0809899
50	C	10.7072953	-3.5508101	-1.6025297
51	C	-8.2252431	-4.9114529	1.6633421
52	C	-8.3256368	-6.1624617	2.2727474
53	C	-9.0043175	-7.2091825	1.6413245
54	C	-9.5763435	-6.9907617	0.3843403
55	C	-9.4663046	-5.7477865	-0.2397181
56	C	-11.0901449	-2.9468387	-0.3459918
57	C	-12.1879585	-2.4053102	-1.0153735
58	C	-12.0242356	-1.7581409	-2.2439135
59	C	-10.7450898	-1.6666542	-2.8009935
60	C	-9.6441803	-2.2198852	-2.1464897
61	C	7.4349400	-2.7600271	-0.2052027
62	C	-7.4554625	-2.7083318	-0.1875156
63	C	6.5782266	-2.8674205	0.9028843
64	C	5.3766895	-2.1648548	0.9409217
65	C	4.9844544	-1.3175316	-0.1115335
66	C	5.8546017	-1.2135578	-1.2123192
67	C	7.0517267	-1.9223776	-1.2658409
68	C	-7.4352721	-1.3220167	0.0387235

69	C	-6.2293056	-0.6271090	0.0750448
70	C	-4.9959391	-1.2815708	-0.0985142
71	C	-5.0294087	-2.6711181	-0.3166159
72	C	-6.2310089	-3.3727767	-0.3677553
73	H	4.4706278	1.2180583	-1.0148228
74	H	2.5805892	-2.1303055	0.9104660
75	H	2.3536989	2.4272454	-0.9863384
76	H	-2.6083301	-2.0946782	0.9549476
77	H	-4.4571831	1.2366141	-1.0379440
78	H	-2.3411435	2.4449279	-0.9802383
79	H	0.0142158	5.1450090	-2.8268444
80	H	0.0044113	2.7469883	-2.3487342
81	H	0.0116640	2.3831228	2.3995428
82	H	0.0219258	4.0087405	4.2714731
83	H	0.0307243	6.4137076	3.8311668
84	H	0.0421213	11.0902721	-1.7507173
85	H	0.0353671	9.4680929	-3.6301285
86	H	0.0259319	7.0628664	-3.1912403
87	H	0.0337248	8.3302044	3.4670446
88	H	0.0423758	10.7277045	2.9919203
89	H	0.0446855	11.5476182	0.6481019
90	H	6.8414789	-5.3852854	-0.7388658
91	H	6.9163527	-7.7282834	0.0483126
92	H	8.8356584	-8.5330230	1.4199517
93	H	10.6709745	-6.9496782	2.0008328
94	H	10.5806334	-4.6002800	1.2345567
95	H	9.4761815	-0.9869449	0.2680275
96	H	11.5142647	0.1074580	-0.6056822
97	H	13.0617313	-1.1364879	-2.1126304
98	H	12.5274743	-3.4889572	-2.7398742
99	H	10.4753868	-4.5722870	-1.8858237
100	H	-7.7071483	-4.1001401	2.1641518
101	H	-7.8814339	-6.3137707	3.2526027
102	H	-9.0860584	-8.1796751	2.1211533
103	H	-10.1010847	-7.7956895	-0.1227039

104	H	-9.9025236	-5.5888331	-1.2204964
105	H	-11.2231027	-3.4373447	0.6127452
106	H	-13.1742055	-2.4791320	-0.5656815
107	H	-12.8797789	-1.3321025	-2.7590024
108	H	-10.6018320	-1.1734083	-3.7583658
109	H	-8.6560800	-2.1555477	-2.5902815
110	H	6.8622536	-3.4974867	1.7394805
111	H	4.7508729	-2.2472847	1.8248197
112	H	5.5728390	-0.5974725	-2.0612614
113	H	7.6917776	-1.8368218	-2.1379465
114	H	-8.3691176	-0.7921259	0.1959987
115	H	-6.2457441	0.4390327	0.2817475
116	H	-4.1018766	-3.2091342	-0.4897698
117	H	-6.2249512	-4.4411222	-0.5578940

SCF Done: E(RB3LYP) = -3181.00810902 A.U.

Zero-point correction = 0.926912 (Hartree/Particle)
Thermal correction to Energy = 0.982051
Thermal correction to Enthalpy = 0.982995
Thermal correction to Gibbs Free Energy = 0.826477
Sum of electronic and zero-point Energies = -3180.081438
Sum of electronic and thermal Energies = -3180.026299
Sum of electronic and thermal Enthalpies = -3180.025354
Sum of electronic and thermal Free Energies = -3180.181873

Low frequencies --- -0.7509 -0.0050 -0.0014 0.0032 0.4767 1.0741
Low frequencies --- 6.5775 7.7510 7.9216

The Result for the TDDFT calculation

Excited State 1: Singlet-A 2.0330 eV 609.86 nm f=0.0001 <S**2>=0.000
242 -> 246 0.14205
245 -> 246 0.68599
This state for optimization and/or second-order correction.
Total Energy, E(TD-HF/TD-DFT) = -3180.93363841

Copying the excited state density for this state as the 1-particle RhoCI density.

Excited State 2:	Singlet-A	2.4646 eV	503.05 nm	f=0.0006	<S**2>=0.000
244 -> 246	0.70625				
Excited State 3:	Singlet-A	2.6366 eV	470.24 nm	f=0.0021	<S**2>=0.000
242 -> 246	0.60770				
243 -> 246	-0.32002				
245 -> 246	-0.16118				
Excited State 4:	Singlet-A	2.7176 eV	456.22 nm	f=0.5580	<S**2>=0.000
242 -> 246	0.32610				
243 -> 246	0.62096				
Excited State 5:	Singlet-A	3.1044 eV	399.38 nm	f=0.7157	<S**2>=0.000
242 -> 247	0.11723				
245 -> 247	0.67570				
Excited State 6:	Singlet-A	3.3358 eV	371.68 nm	f=0.0001	<S**2>=0.000
241 -> 246	0.69803				
Excited State 7:	Singlet-A	3.4029 eV	364.35 nm	f=0.1553	<S**2>=0.000
242 -> 248	0.13319				
245 -> 248	0.65285				
Excited State 8:	Singlet-A	3.5090 eV	353.33 nm	f=0.2988	<S**2>=0.000
244 -> 247	0.53919				
245 -> 249	-0.41542				
Excited State 9:	Singlet-A	3.5458 eV	349.66 nm	f=0.0424	<S**2>=0.000
244 -> 247	0.21468				
244 -> 250	0.28632				
244 -> 251	-0.12274				
245 -> 249	0.21090				
245 -> 250	-0.35191				
245 -> 251	0.35457				

Excited State 10: Singlet-A 3.5591 eV 348.36 nm f=0.0167 <S**2>=0.000
244 -> 250 0.14945
244 -> 251 0.35724
245 -> 250 0.41126
245 -> 251 0.33849

Excited State 11: Singlet-A 3.5663 eV 347.66 nm f=0.0035 <S**2>=0.000
235 -> 246 -0.31362
242 -> 248 0.21297
243 -> 247 0.28860
243 -> 248 0.46899

Excited State 12: Singlet-A 3.5794 eV 346.39 nm f=0.1883 <S**2>=0.000
242 -> 247 -0.28646
243 -> 247 0.10270
244 -> 247 0.24925
244 -> 249 -0.17534
244 -> 250 -0.17461
245 -> 249 0.35731
245 -> 250 0.11046
245 -> 251 -0.23464
245 -> 252 0.10347

Excited State 13: Singlet-A 3.6110 eV 343.35 nm f=0.2437 <S**2>=0.000
242 -> 247 0.39543
243 -> 247 -0.18435
244 -> 247 0.22909
244 -> 249 0.21635
245 -> 249 0.30343
245 -> 252 -0.15865
245 -> 256 -0.11265

Excited State 14: Singlet-A 3.6880 eV 336.18 nm f=0.0012 <S**2>=0.000
242 -> 247 0.22439
242 -> 252 0.11221

243 -> 247 -0.11798
245 -> 248 0.10022
245 -> 250 -0.11930
245 -> 252 0.57484
245 -> 255 0.10356

Excited State 15: Singlet-A 3.7396 eV 331.55 nm f=0.0025 <S**2>=0.000
240 -> 246 0.68153

Excited State 16: Singlet-A 3.7762 eV 328.33 nm f=0.0005 <S**2>=0.000
235 -> 246 0.23555
242 -> 247 0.25131
243 -> 247 0.52960
243 -> 248 -0.20327
243 -> 252 -0.13701

Excited State 17: Singlet-A 3.7940 eV 326.79 nm f=0.0650 <S**2>=0.000
242 -> 253 -0.10460
244 -> 253 -0.28351
245 -> 253 0.56324
245 -> 256 -0.14196

Excited State 18: Singlet-A 3.8021 eV 326.10 nm f=0.0507 <S**2>=0.000
244 -> 253 -0.10101
244 -> 254 0.30855
245 -> 252 -0.10431
245 -> 254 0.53323
245 -> 256 0.17149

Excited State 19: Singlet-A 3.8175 eV 324.77 nm f=0.0924 <S**2>=0.000
242 -> 247 0.12684
244 -> 253 0.15436
245 -> 252 -0.20100
245 -> 253 -0.11176
245 -> 254 -0.11011
245 -> 255 0.44806

245 -> 256 -0.12539
245 -> 257 0.19225
245 -> 258 -0.21744

Excited State 20: Singlet-A 3.8258 eV 324.07 nm f=0.1812 <S**2>=0.000
242 -> 249 -0.10415
244 -> 248 0.14681
244 -> 250 0.11782
244 -> 254 -0.18825
245 -> 249 0.11194
245 -> 251 -0.10228
245 -> 253 0.11595
245 -> 254 -0.15033
245 -> 256 0.52274

Excited State 21: Singlet-A 3.8495 eV 322.08 nm f=0.0094 <S**2>=0.000
244 -> 248 0.66802
245 -> 256 -0.15071

Excited State 22: Singlet-A 3.8837 eV 319.24 nm f=0.0505 <S**2>=0.000
242 -> 247 -0.22848
242 -> 248 -0.10033
244 -> 249 0.57322
245 -> 247 0.15434
245 -> 250 -0.10398
245 -> 255 0.17452

Excited State 23: Singlet-A 3.9217 eV 316.15 nm f=0.0014 <S**2>=0.000
226 -> 246 -0.11257
227 -> 246 -0.13323
234 -> 246 -0.21769
235 -> 246 0.35659
242 -> 248 0.10066
242 -> 252 -0.14865
243 -> 247 -0.17378
243 -> 248 0.25132

243 -> 252 -0.33764
243 -> 258 -0.12377

Excited State 24: Singlet-A 3.9352 eV 315.07 nm f=0.0049 <S**2>=0.000
242 -> 250 -0.16614
242 -> 251 -0.15504
244 -> 250 0.18519
244 -> 251 0.39145
245 -> 250 -0.26580
245 -> 251 -0.27691
245 -> 252 -0.12909
245 -> 255 -0.19126
245 -> 256 -0.10433

Excited State 25: Singlet-A 3.9500 eV 313.88 nm f=0.0068 <S**2>=0.000
242 -> 250 0.15109
242 -> 251 -0.17490
244 -> 250 0.35521
244 -> 251 -0.12468
245 -> 250 0.18479
245 -> 251 -0.26672
245 -> 255 0.26384
245 -> 256 -0.13798
245 -> 257 -0.23999
245 -> 258 0.11285

Excited State 26: Singlet-A 3.9577 eV 313.28 nm f=0.0077 <S**2>=0.000
227 -> 246 0.18644
233 -> 246 -0.27569
234 -> 246 0.32006
242 -> 252 -0.10883
242 -> 255 0.10940
243 -> 252 -0.24093
243 -> 255 0.24999
243 -> 257 0.21946
243 -> 258 0.17737

Excited State 27: Singlet-A 3.9747 eV 311.93 nm f=0.0065 <S**2>=0.000
242 -> 250 0.11408
242 -> 257 0.10787
244 -> 249 0.10967
244 -> 250 0.19009
244 -> 251 -0.17993
245 -> 250 0.19368
245 -> 251 -0.10143
245 -> 255 -0.23624
245 -> 257 0.45204
245 -> 258 -0.18688

Excited State 28: Singlet-A 3.9855 eV 311.09 nm f=0.0038 <S**2>=0.000
226 -> 246 0.34237
230 -> 246 -0.18733
233 -> 246 0.35883
235 -> 246 -0.18489
242 -> 252 -0.12844
243 -> 248 -0.15685
243 -> 252 -0.29109

Excited State 29: Singlet-A 4.0168 eV 308.67 nm f=0.0006 <S**2>=0.000
242 -> 248 0.56269
243 -> 248 -0.24317
244 -> 249 0.14240
245 -> 248 -0.18082
245 -> 258 -0.22401

Excited State 30: Singlet-A 4.0692 eV 304.69 nm f=0.0019 <S**2>=0.000
242 -> 249 0.50443
243 -> 249 -0.27899
244 -> 247 -0.12139
244 -> 248 0.11390
244 -> 252 0.26284
245 -> 256 0.13820

Excited State 31: Singlet-A 4.1136 eV 301.40 nm f=0.0004 <S**2>=0.000
226 -> 246 -0.21898
227 -> 246 0.30938
233 -> 246 0.26348
242 -> 255 0.16458
242 -> 257 -0.10530
243 -> 252 0.10928
243 -> 255 0.37152
243 -> 257 -0.23372

Excited State 32: Singlet-A 4.1187 eV 301.03 nm f=0.0101 <S**2>=0.000
242 -> 248 0.19075
242 -> 258 0.10044
244 -> 252 0.22410
245 -> 255 0.13975
245 -> 257 0.33653
245 -> 258 0.44695

Excited State 33: Singlet-A 4.1252 eV 300.55 nm f=0.0106 <S**2>=0.000
242 -> 248 -0.10396
243 -> 249 0.34186
244 -> 250 -0.10969
244 -> 252 0.44748
244 -> 254 0.12906
245 -> 257 -0.13902
245 -> 258 -0.20332

Excited State 34: Singlet-A 4.1300 eV 300.21 nm f=0.0040 <S**2>=0.000
242 -> 249 0.38378
243 -> 249 0.52307
244 -> 252 -0.17438
244 -> 254 -0.10039

Excited State 35: Singlet-A 4.1362 eV 299.75 nm f=0.0221 <S**2>=0.000
242 -> 254 -0.19167

244 -> 252 -0.14248

244 -> 253 -0.14282

244 -> 254 0.45569

245 -> 254 -0.38228

Excited State 36: Singlet-A 4.1503 eV 298.73 nm f=0.0576 <S**2>=0.000

242 -> 253 -0.12790

244 -> 253 -0.32950

244 -> 256 0.35978

244 -> 262 -0.10403

245 -> 253 -0.18698

245 -> 259 0.30661

Excited State 37: Singlet-A 4.1564 eV 298.30 nm f=0.0133 <S**2>=0.000

242 -> 253 0.18079

244 -> 252 -0.11560

244 -> 253 0.37423

244 -> 254 0.13182

244 -> 256 0.21056

244 -> 262 -0.12006

245 -> 253 0.31024

245 -> 259 0.20623

245 -> 262 0.16402

Excited State 38: Singlet-A 4.1902 eV 295.89 nm f=0.0007 <S**2>=0.000

227 -> 246 0.10941

233 -> 246 -0.11883

234 -> 246 -0.22159

238 -> 246 0.33778

239 -> 246 0.53426

Excited State 39: Singlet-A 4.1938 eV 295.64 nm f=0.0442 <S**2>=0.000

244 -> 252 0.22061

244 -> 254 -0.11735

244 -> 255 -0.15279

244 -> 258 0.11974

244 -> 259	-0.23194
245 -> 260	-0.14675
245 -> 261	-0.22233
245 -> 262	0.38786

Excited State 40: Singlet-A 4.1989 eV 295.28 nm f=0.0001 <S**2>=0.000

237 -> 246	-0.36709
238 -> 246	0.49431
239 -> 246	-0.32809

Excited State 41: Singlet-A 4.2165 eV 294.04 nm f=0.0013 <S**2>=0.000

227 -> 246	-0.16448
233 -> 246	0.16617
234 -> 246	0.34803
235 -> 246	0.13970
237 -> 246	0.38157
238 -> 246	0.35009

Excited State 42: Singlet-A 4.2231 eV 293.59 nm f=0.0116 <S**2>=0.000

241 -> 247	-0.11089
244 -> 256	-0.13282
244 -> 260	-0.22270
244 -> 261	0.14550
244 -> 262	-0.17676
245 -> 260	0.30597
245 -> 261	0.27690
245 -> 262	0.25408
245 -> 263	-0.20470

Excited State 43: Singlet-A 4.2297 eV 293.13 nm f=0.0054 <S**2>=0.000

227 -> 246	0.10760
230 -> 246	0.13091
233 -> 246	-0.12395
234 -> 246	-0.29801
235 -> 246	-0.16297
237 -> 246	0.40604

239 -> 246 -0.28528
244 -> 261 -0.11308
245 -> 260 0.11881
245 -> 261 -0.12199

Excited State 44: Singlet-A 4.2318 eV 292.98 nm f=0.0100 <S**2>=0.000
234 -> 246 0.10394
237 -> 246 -0.10861
242 -> 260 -0.12533
244 -> 256 0.15008
244 -> 259 0.14317
244 -> 260 -0.14529
244 -> 261 -0.27998
245 -> 259 -0.10653
245 -> 260 0.38505
245 -> 261 -0.23729
245 -> 263 0.17981

Excited State 45: Singlet-A 4.2371 eV 292.61 nm f=0.0568 <S**2>=0.000
241 -> 247 -0.14236
244 -> 256 -0.25572
244 -> 259 0.11850
244 -> 260 0.24320
244 -> 261 -0.12555
244 -> 262 -0.16087
245 -> 259 0.28527
245 -> 261 -0.21197
245 -> 263 -0.27832

Excited State 46: Singlet-A 4.2604 eV 291.02 nm f=0.0114 <S**2>=0.000
241 -> 247 -0.11562
242 -> 251 0.20192
242 -> 252 -0.11989
242 -> 256 -0.12481
243 -> 251 -0.10473
244 -> 250 0.10419

244 -> 255	0.46007
244 -> 256	0.15211
244 -> 257	0.10710
245 -> 259	-0.13277
245 -> 261	-0.14750
245 -> 263	-0.15307

Excited State 47: Singlet-A 4.2617 eV 290.92 nm f=0.0033 <S**2>=0.000

241 -> 247	-0.10829
242 -> 250	0.41575
242 -> 251	-0.12173
242 -> 252	-0.16484
243 -> 250	-0.21504
244 -> 250	-0.17503
244 -> 251	0.19224
244 -> 255	-0.19397
244 -> 256	0.13927
245 -> 263	-0.11999

Excited State 48: Singlet-A 4.2703 eV 290.34 nm f=0.0116 <S**2>=0.000

241 -> 247	0.11042
242 -> 250	0.17061
242 -> 251	-0.26502
242 -> 252	0.12314
243 -> 251	0.13565
244 -> 250	-0.18410
244 -> 255	0.32443
244 -> 256	-0.16655
244 -> 262	-0.10353
245 -> 259	0.14825
245 -> 262	0.20747
245 -> 263	0.16615

Excited State 49: Singlet-A 4.2820 eV 289.54 nm f=0.0003 <S**2>=0.000

236 -> 246	0.69390

Excited State 50: Singlet-A 4.2878 eV 289.16 nm f=0.0096 <S**2>=0.000

242 -> 250	0.23854
242 -> 251	0.37641
243 -> 250	-0.11954
243 -> 251	-0.20156
244 -> 250	0.14779
244 -> 251	0.17669
244 -> 256	-0.22423
245 -> 259	0.19482
245 -> 263	0.19093

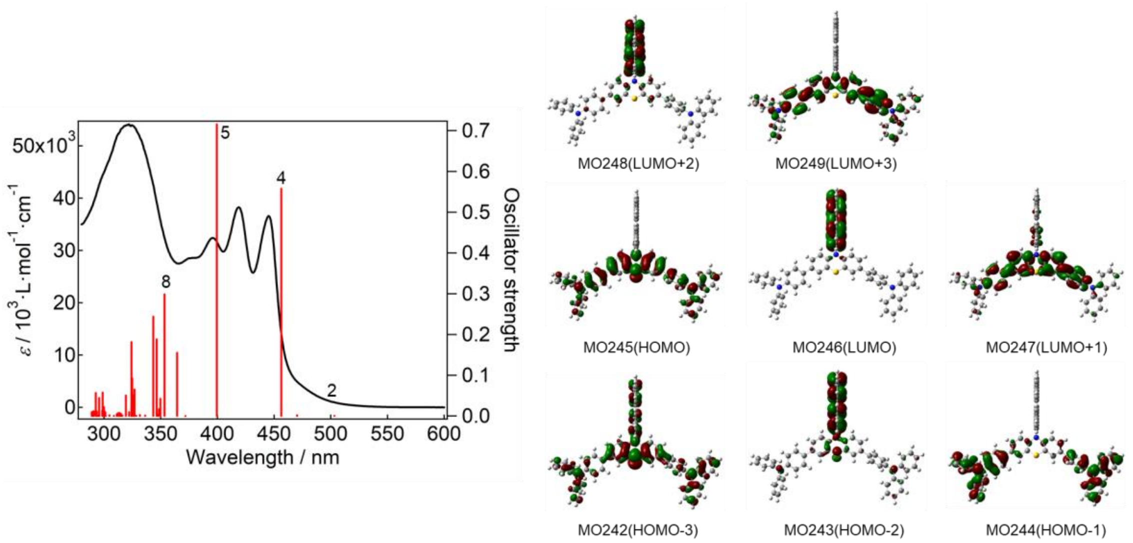

Figure S37. UV–vis absorption spectrum of Pe–PTZ(TPA)$_2$ in benzene at room temperature. The calculated absorption spectrum B3LYP/6-31+G(d,p)//B3LYP/6-31G(d) level of the theory) is shown by the red vertical lines. The relevant molecular orbitals of Pe–PTZ(TPA)$_2$ calculated at the B3LYP/6-31+G(d,p) level of the theory.

Table S5. Standard orientation of the optimized geometry for Pe–Ph–PTZ(TPA)$_2$.

Tag	Symbol	Coordinates		
		X	Y	Z
1	C	-4.8453537	-11.2768628	1.4291636
2	C	-4.2232613	-10.0213815	1.2933801
3	C	-4.8382936	-8.9623025	0.6296635
4	C	-6.1411403	-9.1684525	0.0708245
5	C	-6.7710887	-10.4517458	0.2076022
6	C	-6.0971921	-11.4931384	0.8970420
7	C	-6.8252930	-8.1189688	-0.6240069
8	C	-8.0854194	-8.3854237	-1.1544401
9	C	-8.7001264	-9.6447921	-1.0195117
10	C	-8.0591006	-10.6629317	-0.3493773
11	C	-4.1896327	-7.6440464	0.4826653
12	C	-4.8648051	-6.5911202	-0.2182579
13	C	-6.1790323	-6.7961104	-0.7585698
14	C	-2.9307648	-7.3805599	1.0117124
15	C	-2.3042212	-6.1353945	0.8523103
16	C	-2.9105116	-5.0998790	0.1610080
17	C	-4.2278384	-5.3073314	-0.3690649
18	C	-4.9402186	-4.2591565	-1.0112664
19	C	-6.2065770	-4.4724396	-1.5094470
20	C	-6.8157430	-5.7326906	-1.3917441
21	N	0.0678624	-0.2184331	-0.4733807
22	C	1.4830127	-0.2848365	-0.3857899
23	C	2.2826808	0.7886008	-0.8262434
24	S	1.5388729	2.1788770	-1.6525950
25	C	-0.0235865	2.2166347	-0.8004490
26	C	-0.6205663	1.0179264	-0.3618134
27	C	-0.6810387	3.4383154	-0.6593502
28	C	-1.9825819	3.5211156	-0.1358149
29	C	-2.5861490	2.3193718	0.2642125
30	C	-1.9187653	1.0991261	0.1689439
31	C	2.1427896	-1.4165689	0.1214752
32	C	3.5339386	-1.4706925	0.1940112

33	C	4.3339901	-0.3902023	-0.2071536
34	C	3.6714135	0.7433343	-0.7081450
35	C	-2.6831250	4.8221734	-0.0168631
36	C	5.8126746	-0.4382815	-0.1131186
37	C	6.4563919	-1.0830327	0.9590636
38	C	7.8445750	-1.1266353	1.0570325
39	C	8.6502260	-0.5334216	0.0708508
40	C	8.0198476	0.1080205	-1.0082698
41	C	6.6307895	0.1579882	-1.0901434
42	C	-2.5284262	5.8314929	-0.9847556
43	C	-3.1908132	7.0516436	-0.8789571
44	C	-4.0298456	7.3176229	0.2158455
45	C	-4.1874597	6.3206199	1.1929153
46	C	-3.5313792	5.0986462	1.0711396
47	N	10.0668434	-0.5807632	0.1628291
48	C	10.7134124	-1.7772135	0.5787529
49	C	10.8394610	0.5682789	-0.1630363
50	N	-4.7011742	8.5639319	0.3323490
51	C	-4.0195788	9.7710088	0.0131253
52	C	-6.0535912	8.6058435	0.7701094
53	C	12.0185963	0.4449455	-0.9173546
54	C	12.7773181	1.5737753	-1.2282516
55	C	12.3682204	2.8435399	-0.8096458
56	C	11.1909631	2.9699378	-0.0659986
57	C	10.4350813	1.8445700	0.2637059
58	C	-2.6908792	9.9704411	0.4245838
59	C	-2.0232120	11.1521806	0.1017164
60	C	-2.6696399	12.1603359	-0.6198524
61	C	-3.9944435	11.9678428	-1.0232937
62	C	-4.6642336	10.7823383	-0.7191397
63	C	-6.9866249	7.6677469	0.2964820
64	C	-8.3103994	7.7071730	0.7352436
65	C	-8.7322073	8.6882556	1.6377473
66	C	-7.8079396	9.6280533	2.1040090
67	C	-6.4783446	9.5858031	1.6834284

68	C	10.2808810	-3.0258510	0.1005536
69	C	10.9128083	-4.1976361	0.5177732
70	C	11.9939299	-4.1468484	1.4029605
71	C	12.4316607	-2.9052077	1.8737711
72	C	11.7950613	-1.7296519	1.4746571
73	C	-0.6835270	-1.4331772	-0.2932986
74	C	-0.9779988	-1.9318540	0.9805957
75	C	-1.7124176	-3.1105976	1.1195496
76	C	-2.1721629	-3.8159173	-0.0062096
77	C	-1.8654858	-3.3040183	-1.2790129
78	C	-1.1287616	-2.1276363	-1.4217302
79	H	-4.3274708	-12.0731092	1.9557105
80	H	-3.2365895	-9.8978979	1.7235176
81	H	-6.5841562	-12.4594526	0.9952569
82	H	-8.6259845	-7.6161203	-1.6925052
83	H	-9.6843859	-9.8048526	-1.4497265
84	H	-8.5261229	-11.6378049	-0.2396425
85	H	-2.3918192	-8.1477587	1.5543655
86	H	-1.3069468	-5.9921706	1.2582296
87	H	-4.4845963	-3.2799439	-1.0947138
88	H	-6.7448145	-3.6622402	-1.9926179
89	H	-7.8116564	-5.8553322	-1.7997373
90	H	-0.1617895	4.3425510	-0.9620581
91	H	-3.6029873	2.3265128	0.6444402
92	H	-2.4258993	0.1986590	0.4915059
93	H	1.5677895	-2.2760549	0.4419990
94	H	3.9999718	-2.3819668	0.5559555
95	H	4.2445618	1.6142780	-1.0112177
96	H	5.8615296	-1.5254099	1.7528934
97	H	8.3112191	-1.6139815	1.9069259
98	H	8.6234051	0.5600782	-1.7885834
99	H	6.1749211	0.6362876	-1.9521981
100	H	-1.9098957	5.6467282	-1.8582497
101	H	-3.0664841	7.8024724	-1.6524890
102	H	-4.8189608	6.5111730	2.0545776

103	H	-3.6506078	4.3600251	1.8584835
104	H	12.3356714	-0.5361135	-1.2557071
105	H	13.6859562	1.4593053	-1.8127335
106	H	12.9574248	3.7206946	-1.0590719
107	H	10.8629053	3.9487103	0.2726779
108	H	9.5286018	1.9485491	0.8510291
109	H	-2.1870187	9.1969279	0.9948965
110	H	-0.9961501	11.2889435	0.4284502
111	H	-2.1491686	13.0813564	-0.8639971
112	H	-4.5086338	12.7386131	-1.5906282
113	H	-5.6885353	10.6348017	-1.0455502
114	H	-6.6690338	6.9109390	-0.4133143
115	H	-9.0177207	6.9740192	0.3576634
116	H	-9.7646604	8.7200514	1.9721240
117	H	-8.1179250	10.3927984	2.8107115
118	H	-5.7639911	10.3113138	2.0586192
119	H	9.4501484	-3.0718630	-0.5960144
120	H	10.5650880	-5.1538383	0.1367898
121	H	12.4876256	-5.0601985	1.7205185
122	H	13.2661122	-2.8488518	2.5672209
123	H	12.1329315	-0.7704463	1.8531048
124	H	-0.6362260	-1.3932747	1.8595729
125	H	-1.9459983	-3.4833540	2.1126162
126	H	-2.1918569	-3.8415345	-2.1642157
127	H	-0.8880182	-1.7415644	-2.4074452

SCF Done: E(RB3LYP) = -3412.07521126 A.U.

Zero-point correction = 1.007423 (Hartree/Particle)
Thermal correction to Energy = 1.067368
Thermal correction to Enthalpy = 1.068312
Thermal correction to Gibbs Free Energy = 0.899925
Sum of electronic and zero-point Energies = -3411.068042
Sum of electronic and thermal Energies = -3411.008097
Sum of electronic and thermal Enthalpies = -3411.007152

Sum of electronic and thermal Free Energies = -3411.175540

Low frequencies --- -0.8999 -0.7789 -0.0022 -0.0014 0.0008 0.7789
Low frequencies --- 6.0875 6.5050 6.9274

The Result for the TDDFT calculation

Excited State 1: Singlet-A 2.2540 eV 550.06 nm f=0.0003 <S**2>=0.000
265 -> 266 0.70234
This state for optimization and/or second-order correction.
Total Energy, E(TD-HF/TD-DFT) = -3411.99263052
Copying the excited state density for this state as the 1-particle RhoCI density.

Excited State 2: Singlet-A 2.5991 eV 477.03 nm f=0.0003 <S**2>=0.000
264 -> 266 0.70674

Excited State 3: Singlet-A 2.6904 eV 460.84 nm f=0.6415 <S**2>=0.000
263 -> 266 0.70313

Excited State 4: Singlet-A 2.8101 eV 441.21 nm f=0.0003 <S**2>=0.000
262 -> 266 0.70138

Excited State 5: Singlet-A 3.0791 eV 402.67 nm f=0.5711 <S**2>=0.000
262 -> 267 -0.13294
265 -> 267 0.67542

Excited State 6: Singlet-A 3.2692 eV 379.25 nm f=0.2353 <S**2>=0.000
262 -> 268 -0.14995
265 -> 268 0.65844

Excited State 7: Singlet-A 3.4363 eV 360.81 nm f=0.1427 <S**2>=0.000
262 -> 271 -0.10269
265 -> 269 0.47159
265 -> 271 0.44079
265 -> 273 0.14628

Excited State 8: Singlet-A 3.4993 eV 354.31 nm f=0.3355 <S**2>=0.000
264 -> 267 0.38964
264 -> 268 0.10593
265 -> 270 0.53274

Excited State 9: Singlet-A 3.5399 eV 350.25 nm f=0.0391 <S**2>=0.000
262 -> 272 0.13577
264 -> 267 0.29011
264 -> 271 0.17048
264 -> 273 -0.27455
265 -> 270 -0.11641
265 -> 272 0.50269

Excited State 10: Singlet-A 3.5434 eV 349.91 nm f=0.0036 <S**2>=0.000
255 -> 266 -0.24979
263 -> 267 0.23767
263 -> 268 -0.17272
263 -> 269 0.51932
263 -> 271 -0.16809
263 -> 276 0.10428

Excited State 11: Singlet-A 3.5497 eV 349.28 nm f=0.0036 <S**2>=0.000
262 -> 273 0.12368
264 -> 272 -0.36600
265 -> 271 -0.23403
265 -> 273 0.50286

Excited State 12: Singlet-A 3.5691 eV 347.38 nm f=0.0023 <S**2>=0.000
261 -> 266 0.70036

Excited State 13: Singlet-A 3.5782 eV 346.50 nm f=0.0060 <S**2>=0.000
262 -> 270 0.14454
264 -> 267 0.45073
264 -> 273 0.16073
265 -> 270 -0.37369
265 -> 272 -0.25053

265 -> 277 -0.12835

Excited State 14: Singlet-A 3.6132 eV 343.14 nm f=0.2242 <S**2>=0.000
255 -> 266 0.14096
262 -> 267 0.39872
262 -> 268 0.10571
263 -> 267 0.23835
263 -> 268 -0.30825
263 -> 269 -0.13253
264 -> 270 0.23098
265 -> 269 0.14751
265 -> 278 -0.10627

Excited State 15: Singlet-A 3.6157 eV 342.90 nm f=0.1704 <S**2>=0.000
255 -> 266 -0.18108
262 -> 267 0.33803
263 -> 267 -0.25105
263 -> 268 0.39383
263 -> 269 0.17053
264 -> 270 0.18901

Excited State 16: Singlet-A 3.6865 eV 336.32 nm f=0.0019 <S**2>=0.000
262 -> 267 -0.18460
265 -> 269 0.44897
265 -> 271 -0.41079
265 -> 273 -0.23423

Excited State 17: Singlet-A 3.7813 eV 327.89 nm f=0.0052 <S**2>=0.000
262 -> 270 0.10563
264 -> 267 -0.15546
264 -> 268 0.43434
264 -> 275 -0.15981
265 -> 274 0.34867
265 -> 277 -0.28910

Excited State 18: Singlet-A 3.7838 eV 327.67 nm f=0.0065 <S**2>=0.000

262 -> 267 -0.14318
264 -> 274 -0.27982
265 -> 275 0.51769
265 -> 276 0.14298
265 -> 278 -0.19431
265 -> 279 0.14249

Excited State 19: Singlet-A 3.7864 eV 327.44 nm f=0.0812 <S**2>=0.000
264 -> 268 0.47204
264 -> 275 0.23293
265 -> 274 -0.41903

Excited State 20: Singlet-A 3.8030 eV 326.02 nm f=0.0066 <S**2>=0.000
256 -> 266 0.12320
263 -> 267 0.50770
263 -> 268 0.36326
263 -> 271 0.19781
263 -> 273 0.10589

Excited State 21: Singlet-A 3.8095 eV 325.46 nm f=0.1013 <S**2>=0.000
262 -> 267 0.20211
263 -> 267 -0.10321
264 -> 272 -0.10094
264 -> 274 -0.18819
265 -> 275 0.26824
265 -> 276 -0.27763
265 -> 278 0.32462
265 -> 279 -0.22112

Excited State 22: Singlet-A 3.8135 eV 325.12 nm f=0.1953 <S**2>=0.000
262 -> 272 0.10963
264 -> 268 0.19189
264 -> 273 -0.11567
264 -> 275 -0.19218
265 -> 270 -0.13037
265 -> 272 -0.10512

265 -> 274 0.23143
265 -> 276 0.11916
265 -> 277 0.50330

Excited State 23: Singlet-A 3.8752 eV 319.94 nm f=0.0340 <S**2>=0.000
262 -> 267 -0.26845
262 -> 268 0.21555
264 -> 270 0.52801
265 -> 267 -0.16019
265 -> 276 -0.11718

Excited State 24: Singlet-A 3.8843 eV 319.20 nm f=0.0029 <S**2>=0.000
251 -> 266 -0.16260
253 -> 266 0.15464
255 -> 266 0.25904
256 -> 266 0.38233
260 -> 266 -0.17716
263 -> 267 -0.17213
263 -> 268 -0.11744
263 -> 269 0.24804
263 -> 271 0.14270
263 -> 276 0.14099
263 -> 280 0.10541

Excited State 25: Singlet-A 3.9176 eV 316.48 nm f=0.0038 <S**2>=0.000
262 -> 272 0.12204
264 -> 268 0.10540
264 -> 269 0.55583
264 -> 271 0.28654
265 -> 272 -0.19333
265 -> 277 -0.13111

Excited State 26: Singlet-A 3.9318 eV 315.34 nm f=0.0010 <S**2>=0.000
262 -> 268 0.20574
262 -> 271 0.12842
262 -> 273 -0.23376

264 -> 272 0.43079
265 -> 271 -0.16121
265 -> 273 0.33670
265 -> 276 -0.14935

Excited State 27: Singlet-A 3.9365 eV 314.96 nm f=0.0154 <S**2>=0.000
252 -> 266 0.12809
253 -> 266 0.26706
256 -> 266 -0.15927
260 -> 266 0.40999
262 -> 272 0.12419
263 -> 271 0.19344
263 -> 273 0.10702
263 -> 278 -0.12835
263 -> 279 -0.12978
263 -> 280 0.12602
264 -> 273 -0.18041
265 -> 272 -0.16459

Excited State 28: Singlet-A 3.9402 eV 314.67 nm f=0.0037 <S**2>=0.000
253 -> 266 0.15242
256 -> 266 -0.10188
260 -> 266 0.15263
262 -> 272 -0.22022
263 -> 271 0.10243
264 -> 269 0.31245
264 -> 271 -0.12245
264 -> 273 0.33220
265 -> 272 0.28152
265 -> 277 0.15465

Excited State 29: Singlet-A 3.9559 eV 313.41 nm f=0.0013 <S**2>=0.000
251 -> 266 -0.11051
252 -> 266 -0.11166
253 -> 266 -0.22187
255 -> 266 0.12646

256 -> 266 0.25199
260 -> 266 0.50663
263 -> 271 -0.12900
263 -> 278 0.10726
263 -> 280 -0.10137

Excited State 30: Singlet-A 3.9626 eV 312.89 nm f=0.0010 <S**2>=0.000
262 -> 268 0.54980
264 -> 270 -0.27679
264 -> 272 -0.13604
265 -> 268 0.18353
265 -> 279 0.12705

Excited State 31: Singlet-A 4.0035 eV 309.69 nm f=0.0117 <S**2>=0.000
251 -> 266 0.16721
253 -> 266 -0.25271
255 -> 266 -0.10491
263 -> 269 0.15680
263 -> 271 0.45132
263 -> 273 0.22858
263 -> 279 0.23479
263 -> 280 -0.13945

Excited State 32: Singlet-A 4.0565 eV 305.64 nm f=0.0009 <S**2>=0.000
262 -> 270 -0.35577
264 -> 269 -0.24444
264 -> 271 0.42654
264 -> 273 0.25200

Excited State 33: Singlet-A 4.0571 eV 305.60 nm f=0.0006 <S**2>=0.000
251 -> 266 0.34962
252 -> 266 0.12893
263 -> 268 0.10938
263 -> 276 0.49987
263 -> 278 0.20129

Excited State 34: Singlet-A 4.0798 eV 303.90 nm f=0.0116 <S**2>=0.000

251 -> 266 0.11985

255 -> 266 -0.17744

256 -> 266 0.16205

262 -> 268 0.15152

263 -> 280 0.10764

265 -> 276 0.46930

265 -> 278 0.24879

265 -> 279 -0.18687

Excited State 35: Singlet-A 4.0851 eV 303.51 nm f=0.0315 <S**2>=0.000

254 -> 266 -0.10216

255 -> 266 0.28598

256 -> 266 -0.23819

262 -> 270 -0.17178

263 -> 268 0.12392

263 -> 276 0.18444

263 -> 280 -0.17017

264 -> 271 -0.21380

264 -> 273 -0.12671

265 -> 276 0.26002

265 -> 278 0.13934

265 -> 279 -0.10080

Excited State 36: Singlet-A 4.0969 eV 302.63 nm f=0.0020 <S**2>=0.000

255 -> 266 0.15519

256 -> 266 -0.14417

262 -> 270 0.48072

263 -> 270 0.15977

263 -> 280 -0.10189

264 -> 267 -0.10288

264 -> 271 0.27403

264 -> 273 0.15708

265 -> 277 0.10867

Excited State 37: Singlet-A 4.1081 eV 301.80 nm f=0.0096 <S**2>=0.000

262 -> 269 0.55530
262 -> 271 0.26605
262 -> 273 0.14299
264 -> 277 -0.13185
265 -> 271 0.11538

Excited State 38: Singlet-A 4.1177 eV 301.10 nm f=0.0003 <S**2>=0.000
262 -> 270 -0.12051
263 -> 270 0.68143

Excited State 39: Singlet-A 4.1336 eV 299.95 nm f=0.0226 <S**2>=0.000
262 -> 275 -0.24998
264 -> 274 0.52558
265 -> 275 0.37753

Excited State 40: Singlet-A 4.1348 eV 299.86 nm f=0.0611 <S**2>=0.000
262 -> 274 -0.24602
264 -> 275 0.51482
265 -> 274 0.36864

Excited State 41: Singlet-A 4.1463 eV 299.02 nm f=0.0289 <S**2>=0.000
262 -> 267 0.10290
262 -> 269 0.18112
264 -> 272 -0.10806
264 -> 277 0.37341
264 -> 282 -0.18400
265 -> 281 0.39536
265 -> 284 -0.19487

Excited State 42: Singlet-A 4.1846 eV 296.29 nm f=0.0457 <S**2>=0.000
264 -> 273 -0.14766
264 -> 275 -0.12697
264 -> 278 0.10245
264 -> 281 -0.19877
264 -> 284 0.15490
264 -> 286 0.12375

265 -> 279 -0.10282
265 -> 282 0.47554
265 -> 283 0.19580

Excited State 43: Singlet-A 4.1956 eV 295.51 nm f=0.0039 <S**2>=0.000
261 -> 267 -0.11192
262 -> 268 -0.11514
264 -> 283 -0.10211
265 -> 278 0.31006
265 -> 279 0.38776
265 -> 280 0.25310
265 -> 282 0.12891
265 -> 284 -0.11384
265 -> 286 -0.24261

Excited State 44: Singlet-A 4.2186 eV 293.90 nm f=0.0236 <S**2>=0.000
262 -> 269 -0.13723
262 -> 284 0.12331
264 -> 283 0.31185
265 -> 278 0.17489
265 -> 279 0.12362
265 -> 280 0.16619
265 -> 281 0.21568
265 -> 284 0.41678
265 -> 286 0.15579

Excited State 45: Singlet-A 4.2236 eV 293.55 nm f=0.0122 <S**2>=0.000
262 -> 283 0.15834
264 -> 281 0.24194
264 -> 284 0.28754
265 -> 282 -0.21611
265 -> 283 0.49368

Excited State 46: Singlet-A 4.2316 eV 293.00 nm f=0.0563 <S**2>=0.000
261 -> 267 0.19539
262 -> 281 -0.12112

264 -> 277 0.16044
264 -> 282 0.23166
264 -> 283 -0.15289
265 -> 278 0.13747
265 -> 280 0.15471
265 -> 281 -0.20726
265 -> 284 -0.14143
265 -> 285 0.12087
265 -> 286 0.40001

Excited State 47: Singlet-A 4.2431 eV 292.20 nm f=0.0001 <S**2>=0.000
262 -> 269 -0.24633
262 -> 271 0.42074
264 -> 277 -0.20089
265 -> 271 0.10878
265 -> 279 -0.23445
265 -> 280 0.26781
265 -> 281 0.10379
265 -> 284 -0.14543

Excited State 48: Singlet-A 4.2701 eV 290.35 nm f=0.0040 <S**2>=0.000
262 -> 271 0.24391
262 -> 273 -0.16734
264 -> 272 -0.15368
264 -> 277 0.41015
265 -> 279 -0.13038
265 -> 281 -0.27666
265 -> 284 0.16230
265 -> 286 -0.16107

Excited State 49: Singlet-A 4.2744 eV 290.06 nm f=0.0010 <S**2>=0.000
262 -> 272 0.53358
262 -> 277 -0.15268
263 -> 272 0.21274
264 -> 271 -0.15024
264 -> 273 0.25927

265 -> 280 -0.10707

Excited State 50: Singlet-A 4.2750 eV 290.02 nm f=0.0004 <S**2>=0.000

262 -> 269 0.11173

262 -> 271 -0.27134

262 -> 273 0.18481

264 -> 272 0.16378

264 -> 277 0.12508

265 -> 278 -0.12531

265 -> 279 -0.21596

265 -> 280 0.43448

265 -> 285 0.11388

265 -> 286 -0.10286

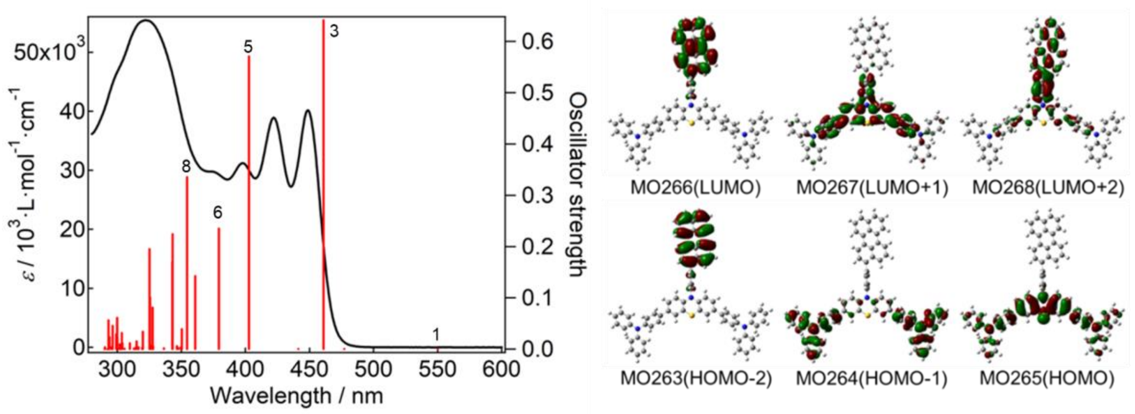

Figure S38. UV–vis absorption spectrum of Pe–Ph–PTZ(TPA)2 in benzene at room temperature. The calculated absorption spectrum B3LYP/6-31+G(d,p)//B3LYP/6-31G(d) level of the theory) is shown by the red vertical lines. The relevant molecular orbitals of Pe–Ph–PTZ(TPA)2 calculated at the B3LYP/6-31+G(d,p) level of the theory.

10. References

[1] Würth, C.; Grabolle, M.; Pauli, J.; Spieles, M.; Resch-Genger, U. *Nat. Protoc.* **2013**, 8 (8), 1535–1550.

[2] Nagarajan, S.; Barthes, C.; Girdhar, N. K.; Dang, T. T.; Gourdon, A. *Tetrahedron*. **2012**, *68*(46), 9371–9375.

[3] Franz, A. W.; Rominger, F.; Müller, T. J. J. *J. Org. Chem*, **2008**, *73*(5), 1795–1802.

[4] Urselmann, D.; Deilhof, K.; Mayer, B.; Müller, T. J. J. *Beilstein J. Org. Chem*. **2016**, *12*, 2055–2064.

[5] Kawanishi, Y.; Mutoh, K.; Abe, J.; Kobayashi, Y. *Asian J. Org. Chem.* **2021**, *10*(4), 891–900.

[6] Zhu, X. Q.; Dai, Z.; Yu, A.; Wu, S.; Cheng, J. P. *J. Phys. Chem. B*, **2008**, *112*(37), 11694–11707.

[7] Avlasevich, Y.; Müllen, K. *J. Org. Chem.* **2007**, *72*(26), 10243–10246.

[8] Fritz, Y.; Wagenknecht, H. A. *Front. Chem.* **2019**, *7*.

[9] M. J. Frisch, G. W. Trucks, H. B. Schlegel, G. E. Scuseria, M. A. Robb, J. R. Cheeseman, G. Scalmani, V. Barone, G. A. Petersson, H. Nakatsuji, X. Li, M. Caricato, A. V. Marenich, J. Bloino, B. G. Janesko, R. Gomperts, B. Mennucci, H. P. Hratchian, J. V. Ortiz, A. F. Izmaylov, J. L. Sonnenberg, D. Williams-Young, F. Ding, F. Lipparini, F. Egidi, J. Goings, B. Peng, A. Petrone, T. Henderson, D. Ranasinghe, V. G. Zakrzewski, J. Gao, N. Rega, G. Zheng, W. Liang, M. Hada, M. Ehara, K. Toyota, R. Fukuda, J. Hasegawa, M. Ishida, T. Nakajima, Y. Honda, O. Kitao, H. Nakai, T. Vreven, K. Throssell, J. A. Montgomery, Jr., J. E. Peralta, F. Ogliaro, M. J. Bearpark, J. J. Heyd, E. N. Brothers, K. N. Kudin, V. N. Staroverov, T. A. Keith, R. Kobayashi, J. Normand, K. Raghavachari, A. P. Rendell, J. C. Burant, S. S. Iyengar, J. Tomasi, M. Cossi, J. M. Millam, M. Klene, C. Adamo, R. Cammi, J. W. Ochterski, R. L. Martin, K. Morokuma, O. Farkas, J. B. Foresman, and D. J. Fox, Gaussian, Inc., Wallingford CT, **2016**.
